# A New EBS2b-IBS2b Base Paring (A_−8_/T_−8_) Improved the Gene-Targeting Efficiency of Thermotargetron in Escherichia coli

**DOI:** 10.1128/spectrum.03159-22

**Published:** 2023-02-21

**Authors:** Guzhen Cui, Dengxiong Hua, Xingxing Zhao, Jia Zhou, Ying Yang, Tingyu Huang, Xinxin Wang, Yan Zhao, Ting Zhang, Jian Liao, Zhizhong Guan, Peng Luo, Zhenghong Chen, Xiaolan Qi, Wei Hong

**Affiliations:** a Key Laboratory of Endemic and Ethnic Diseases, Ministry of Education & Key Laboratory of Medical Molecular Biology of Guizhou Province & Key Laboratory of Microbiology and Parasitology of Education Department of Guizhou, Guizhou Medical University, Guiyang, Guizhou, China; b Collaborative Innovation Center for Prevention and Control of Endemic and Ethnic Regional Diseases Co-constructed by the Province and Ministry; c Joint Laboratory of Helicobacter Pylori and Intestinal Microecology of Affiliated Hospital of Guizhou Medical University; d School/Hospital of Stomatology, Guizhou Medical University, Guiyang, Guizhou, China; Ocean University of China

**Keywords:** Thermotargetron (TMT), ClosTron, Intron RNA, EBS2b-IBS2b, gene-targeting efficiency, group II intron

## Abstract

Thermophilic group II intron is one type of retrotransposon composed of intron RNA and intron-encoded protein (IEP), which can be utilized in gene targeting by harnessing their novel ribozyme-based DNA integration mechanism termed “retrohoming.” It is mediated by a ribonucleoprotein (RNP) complex that contains the excised intron lariat RNA and an IEP with reverse transcriptase (RT) activity. The RNP recognizes targeting sites by exon-binding sequences 2 (EBS2)/intron-binding sequences 2 (IBS2), EBS1/IBS1, and EBS3/IBS3 bases pairing. Previously, we developed the TeI3c/4c intron as a thermophilic gene targeting system—Thermotargetron (TMT). However, we found that the targeting efficiency of TMT varies significantly at different targeting sites, which leads to a relatively low success rate. To further improve the success rate and gene-targeting efficiency of TMT, we constructed a Random Gene-targeting Plasmids Pool (RGPP) to analyze the sequence recognition preference of TMT. A new base pairing, located at the −8 site between EBS2/IBS2 and EBS1/IBS1 (named EBS2b-IBS2b), increased the success rate (2.45- to 5.07-fold) and significantly improved gene-targeting efficiency of TMT. A computer algorithm (TMT 1.0), based on the newly discovered sequence recognition roles, was also developed to facilitate the design of TMT gene-targeting primers. The present work could essentially expand the practicalities of TMT in the genome engineering of heat-tolerance mesophilic and thermophilic bacteria.

**IMPORTANCE** The randomized base pairing in the interval of IBS2 and IBS1 of Tel3c/4c intron (−8 and −7 sites) in Thermotargetron (TMT) results in a low success rate and gene-targeting efficiency in bacteria. In the present work, we constructed a randomized gene-targeting plasmids pool (RGPP) to study whether there is a base preference in target sequences. Among all the successful “retrohoming” targets, we found that a new EBS2b-IBS2b base paring (A_−8_/T_−8_) significantly increased TMT's gene-targeting efficiency, and the concept is also applicable to other gene targets in redesigned gene-targeting plasmids pool in E. coli. The improved TMT is a promising tool for the genetic engineering of bacteria and could promote metabolic engineering and synthetic biology research in valuable microbes that recalcitrance for genetic manipulation.

## INTRODUCTION

Group II introns are a large type of retrotransposons composed of intron RNA and intron-encoded protein (IEP) and are widely distributed in bacterial and mitochondria and chloroplast organellar genomes of eukaryotes ([Bibr B1][Bibr B2] 6). Group II introns are also valuable genome editing tools (e.g., Targetron) and the source of reverse transcriptase (RTs) that have broad applications in biotechnology and sequencing of RNAs (1, [Bibr B7][Bibr B8] 10). The Targetron has been successfully applied in a broad host range, especially in some intractable bacteria that are difficult to modify with conventional genetic methods, such as in the Gram-positive anaerobic genus *Clostridium*, which is also specifically referred to as “ClosTron” ([Bibr B11][Bibr B12] 15).

**FIG 1:**
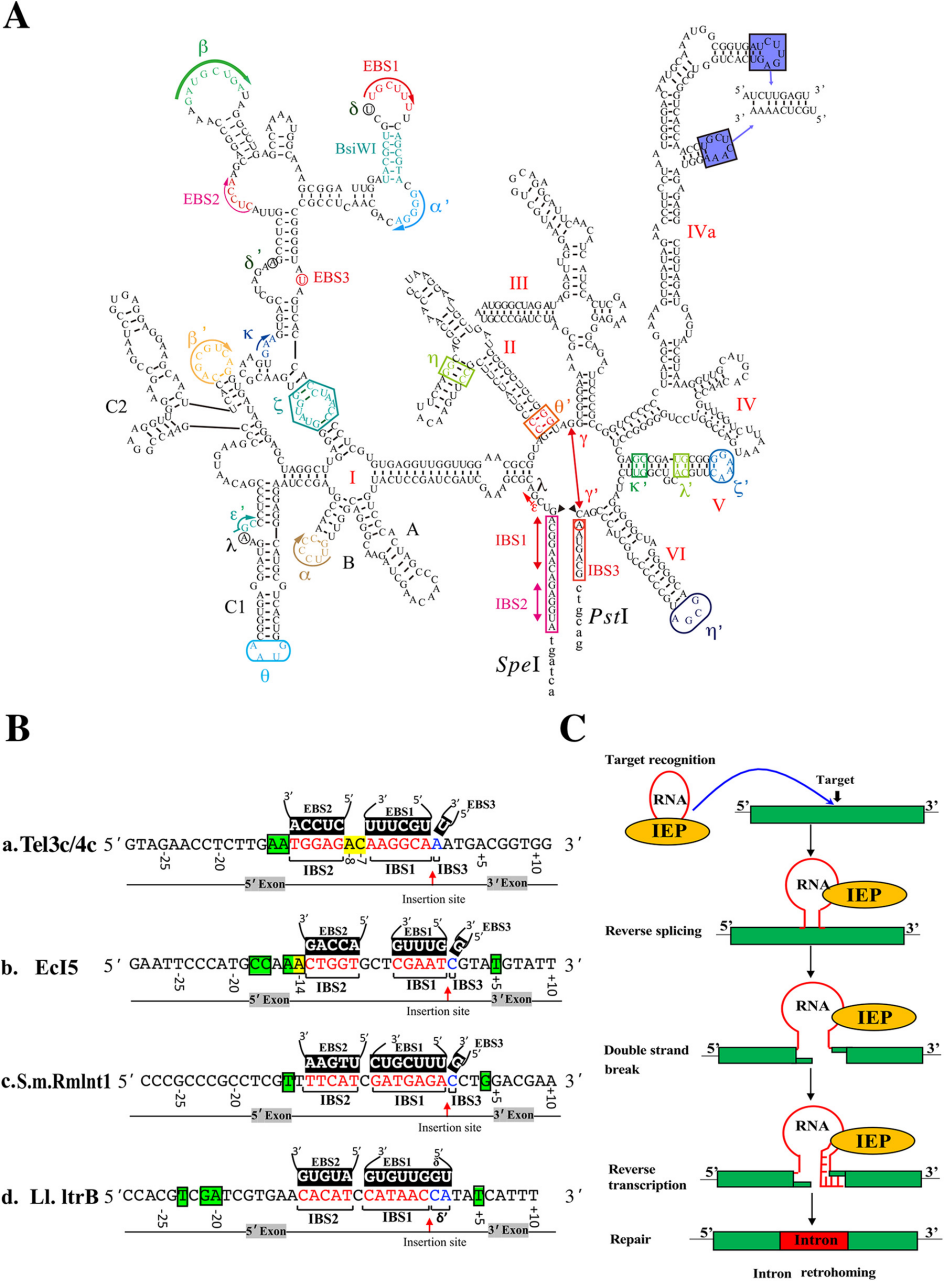
Group II intron and its “retrohoming” mechanism. (A) 2D structure model of the Tel3c group II intron RNA. The recognition sequences are denoted in EBS and IBS. Greek letters denote essential sequence elements involved in predicted interactions at the tertiary structural level. Restriction sites used in plasmid constructions are *BisW*I, SpeI, and PstI. The I to VI represent six different stem-loop structures of Tel3c group II intron RNA. (B) Recognition sequences of different group II intron. (a) Recognition sequences of Tel3c group II intron derived from Thermosynechococcus elongatus. (b) Recognition sequences of EcI5 group II intron derived from Escherichia coli. (c) Recognition sequences of S.m.Rmlnt1 group II intron derived from Sinorhizobium meliloti. (d) Recognition sequences of Ll.ltrB group II intron derived from Lactococcus lactis. The EBS1, EBS2, and EBS3 (δ) involved in intron RNA are denoted in black background. The IBS1, IBS2, and IBS3 (δ′) in the targeting site are denoted in red or blue letters. Red arrows mark insertion sites of intron RNA. IEP recognition sites are marked with green background. Positive numbers represent downstream of the insertion site; negative numbers represent upstream of the insertion site. (C) Schematic representing group II intron “retrohoming” mechanism. (i) The group II intron ribonucleoproteins (RNPs) contain the excised intron lariat RNA and an intron-encoded protein (IEP) with reverse transcriptase (RT) activity, recognize DNA target sequences for intron insertion by using both the IEP and base pairing of the intron RNA (target recognition). (ii) The RNPs recognize DNA target sites primarily by base-pairing sequence motifs in the intron RNA to the DNA target sequence, with assistance from the intron-encoded RT. The intron RNA then uses its catalytic (“ribozyme”) activity to insert into the top strand of the DNA target site (reverse splicing), while the DNA endonuclease activity of the RT is used to cleave the bottom strand, and the nicked DNA is used as a primer for reverse transcription of the inserted intron RNA (double-strand break and reverse transcription). (iii) The resulting intron cDNA is integrated into the genome by host DNA repairing enzymes (repair). Because the DNA target site is recognized mainly by base pairing of the intron RNA, the TMT can be programmed to insert into desired sites simply by modifying the base-pairing motifs in the intron RNA.

Gene targeting on the chromosome by Targetron is realized by their ribozyme-based DNA integration mechanism, which is also named “retrohoming.” It is mediated by a ribonucleoprotein (RNP) complex that is composed of a highly structured catalytic intron RNA and a multifunctional intron-encoded protein (IEP), including reverse transcriptase (RT) activity, maturase activity, and DNA endonuclease activity (EN) ([Bibr B16][Bibr B17] 18). The intron RNA has a typical secondary structure (Fig. 1A) and carries specific exon-binding sequences (EBS) that recognize intron-binding sequences (IBS) on DNA target sites (typically 11 ~ 14 nt) based on base pairing mechanisms. IEP protein also recognizes a small number of DNA bases (typically 2 ~ 5 nt) to assist in the recognition of intron RNA and target sites (Fig. 1A and B). Then, the ribozyme activity of intron RNA cleaves the DNA target sites and inserts intron RNA into them. The nuclease activity of the IEP protein cleaves the DNA reverse chain and synthesizes the complementary chain by its RT activity using the cleaved 3′ end as a primer and the inserted intron RNA as a template. Finally, Targetron utilizes the host’s DNA repair machinery to achieve retrohoming of intron RNA on the chromosome (Fig. 1C) (2, [Bibr B19][Bibr B20] 22). Due to the targeting recognition being mainly determined by the base pairing of intron RNA and target DNA, the target sites can be redirected by simply modifying the EBS sequence of the intron RNA ([Bibr B13][Bibr B14] 15). Typically, the gene targeting using Targetron is precise and efficient, and the targeting frequencies of the widely used Ll.LtrB Group II intron ranging from 1% ~ 100% (varies greatly depending on different species and genes) (9, 10).

Four types of group II introns have been developed as gene inactivation tools (e.g., Targetron), including Ll.LtrB (derived from Lactococcus lactis [10, 23]), EcI5 (derived from Escherichia coli [24]), RmInt1 (derived from Sinorhizobium meliloti [25, 26]), and Tel3c/4c (derived from Thermosynechococcus elongatus, and were constructed by a hybrid Tel3c intron RNA and Tel4c IEP protein [13, 27]). The Tel3c/4c Targetron is also specifically called “Thermotargetron (TMT)” due to its thermophilic and high-temperature stability characteristics. The TMT can be applied in thermophilic microorganisms (e.g., Clostridium thermocellum) and mesophilic microorganisms that could be temporarily tolerant at high temperatures. For example, the TMT system has been demonstrated as a powerful gene-targeting tool in mesophilic E. coli (13). It has also been used to elucidate the contribution of CipA (cellulosome-integrating protein A) and four secondary scaffoldins (OlpB, 7CohII, Orf2p, and SdbA) to the cellulose hydrolysis rate of *C. thermocellum*, which provided new insights into cellulosome function and impact of genetic tools to enhance bioconventions of cellulose substrates (28). Thus, the TMT system has the potential to be more widely used than mesophilic Targetron.

These four kinds of group II intron have a similar tertiary structure and retrohoming mechanisms (5, 19). Their intron RNA comprises six highly conserved domains (from domain I to domain VI). The most important domains for targets recognition are EBS1, EBS2, and EBS3, which recognize IBS1, IBS2, and IBS3 by the Watson-Crick base pairing rule on the chromosome DNA (Fig. 1A). These three bases paring EBS1/IBS1, EBS2/IBS2, and EBS3/IBS3 (referred to as δ/δ’ paring in subgroup IIA introns) determined the targeting specificity to a large extent. The IEP protein also recognizes a small number of DNA bases (usually 2 to 5 bases) in addition to its RT activity and DNA endonuclease activity to assist in the recognition between intron RNA and DNA target sequences (Fig. 1A) (2, 19). Moreover, the IEP protein recognition of the distal 5′-exon sequence could promote DNA melting to enable the intron RNA to base pair to the adjacent DNA target sequences. The IEP recognition of the 3′ exon is also necessary for IEP cleavage of the opposite strand to produce the primer for RT (Fig. 1C) (2, 5, 28).

The EBS and IBS sequences are essential for targeting site recognition and retrohoming efficiency. Except for the paired EBS2/IBS2, EBS1/IBS1, and EBS3/IBS3, we also found another two interval bases located between IBS2 and IBS1, denoted -8 and -7 sites (Fig. 1Ba) (13, 27). The length of the interval sequence between IBS2 and IBS1 is different in different group II introns. For example, to EcI5, the length of the interval sequence is 3 bp. In Tel3c and Ll.ltrB introns, the length is 2 bp and 1 bp, respectively (Fig. 1B) (10, 13, 24). Recently, a new EBS2a-IBS2a base pair (adjacent to EBS2/IBS2) was found in the EcI5 intron, and this paring could greatly affect intron migration on the chromosome, suggesting these adjacent key sequences could play a vital role in target DNA recognition and splicing (29). In our previous experience, the randomized base pairing of -8 and -7 sites in the interval of IBS2 and IBS1 of Tel3c/4c intron of TMT results in an undesirable success rate of gene inactivation. Whether -8 and -7 sites impact TMT retrohoming and gene targeting efficiency and their underlying mechanisms were largely unknown.

**FIG 2:**
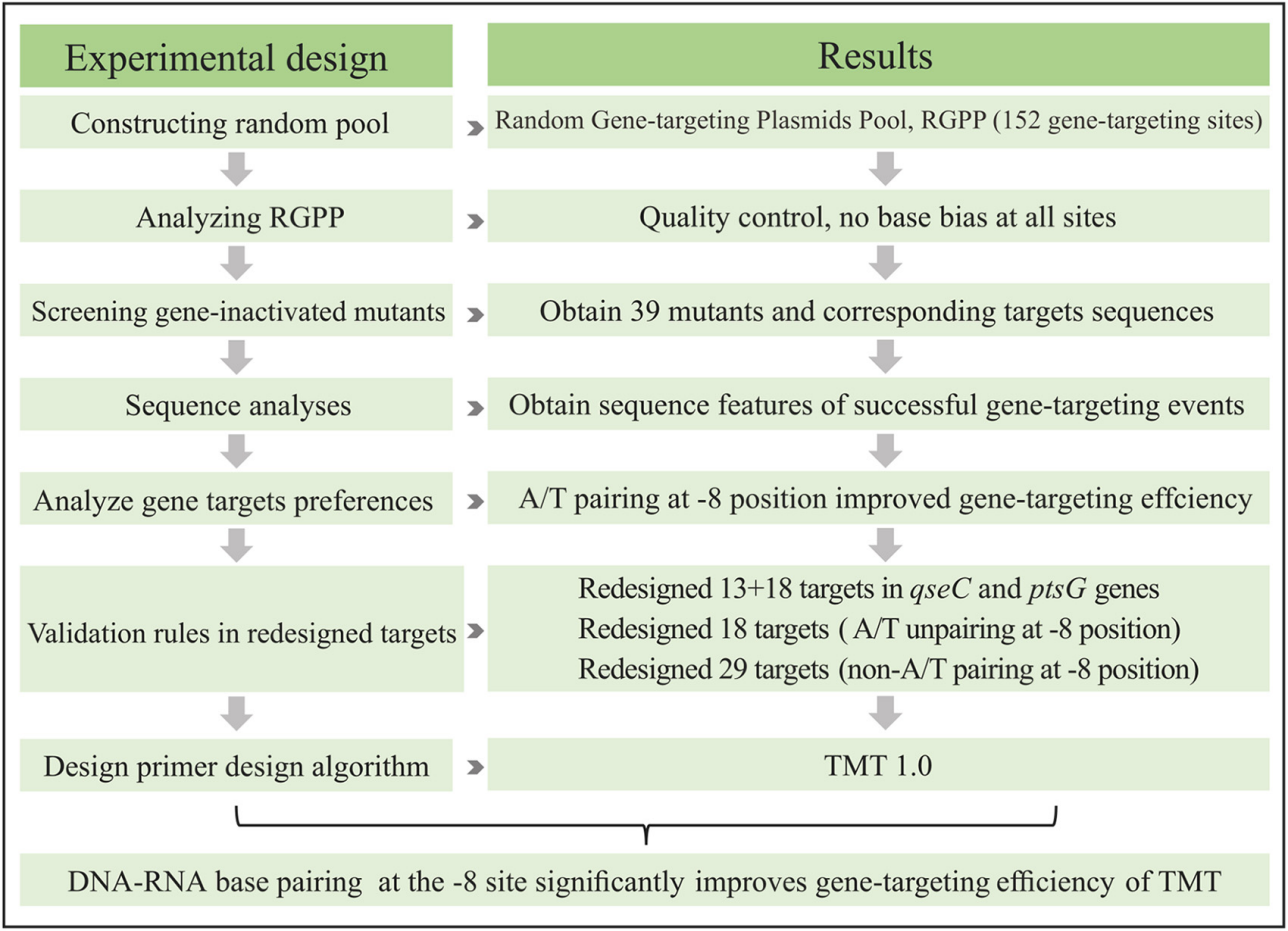
The flow chart of experimental design.

To determine the effect of these interval bases (between IBS2 and IBS, -7 and -8 sites, Fig. 1Ba) on TMT gene-targeting efficiency, we designed 152 random target sites in *fliC* (flagellin C), *lacZ* (β-galactosidase), *dctA* (C4 dicarboxylate), and *glcD* (glycolate oxidase) genes. We analyzed the rules for the successful “retrohoming” of TMT. The results showed that A/T pairing at the -8 position (EBS2b-IBS2b) could significantly improve TMT’s targeting success rate and efficiency. Furthermore, a computer algorithm (TMT 1.0), based on the newly discovered sequence recognition roles, was also developed to facilitate the design of TMT gene targeting primers (Fig. 2; Fig. S1 in the suppplemental material). In conclusion, the present study discovered that a new EBS2b-IBS2b base paring (A_−8_/T_−8_) could significantly improve the gene targeting performance of TMT, which could largely improve the usability and the efficiency of TMT in mesophilic and thermophilic bacteria.

## RESULTS

### Construction of random gene-targeting plasmids pool (RGPP).

**FIG 3:**
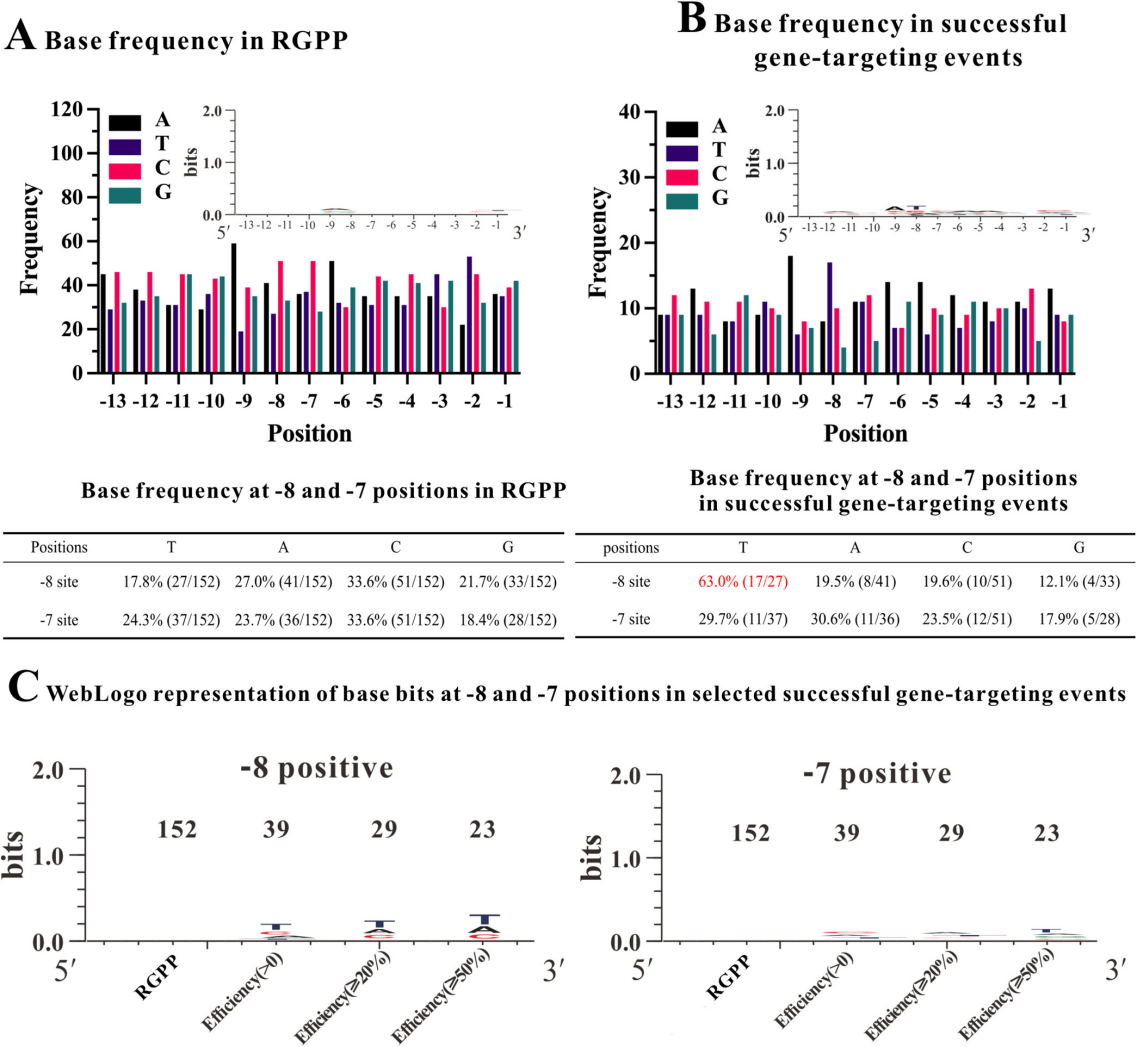
Gene-targeting analyses of Random Gene-targeting Plasmids Pool (RGPP). (A) Base frequency (bar chart) and the table of base frequency at -8 and -7 positions in RGPP. (B) Base frequency (bar chart) and the table of base frequency at -8 and -7 positions in successful gene-targeting events. (C) WebLogo representation of base bits at -8 and -7 positions in successful gene-targeting events.

We designed 152 targeting sites in *fliC* (flagellin C, ECHMS174_01916), *lacZ* (βeta-galactosidase, ECHMS174_00351), *dctA* (C4-dicarboxylate, ECHMS174_03796), *glcD* (glycolate oxidase, ECHMS174_03014), and constructed 152 gene-targeting plasmids (random gene-targeting plasmids pool, RGPP). All plasmids met the following criteria: A_(−15)_A_(−14)_nnnnnnnnnnnnnA_(+1)_ (“n” represents 13 arbitrary nucleotides) (Tables S1, S2, and S3). In the RGPP, 71 and 66 plasmids were designed to inactivate *fliC* and *lacZ* genes, covering almost all the potential target sites of these two genes. In addition, 7 and 8 plasmids were randomly designed to inactivate *dctA* and *glcD* genes. All in all, 152 random plasmids were constructed in RGPP to study the influence of base-pairing on the gene-targeting efficiency of TMT (Table S2). The WebLogo (https://weblogo.berkeley.edu) analysis showed no notable base bias in RGPP (Fig. 3A).

### Gene targeting assay of TMT.

**FIG 4:**
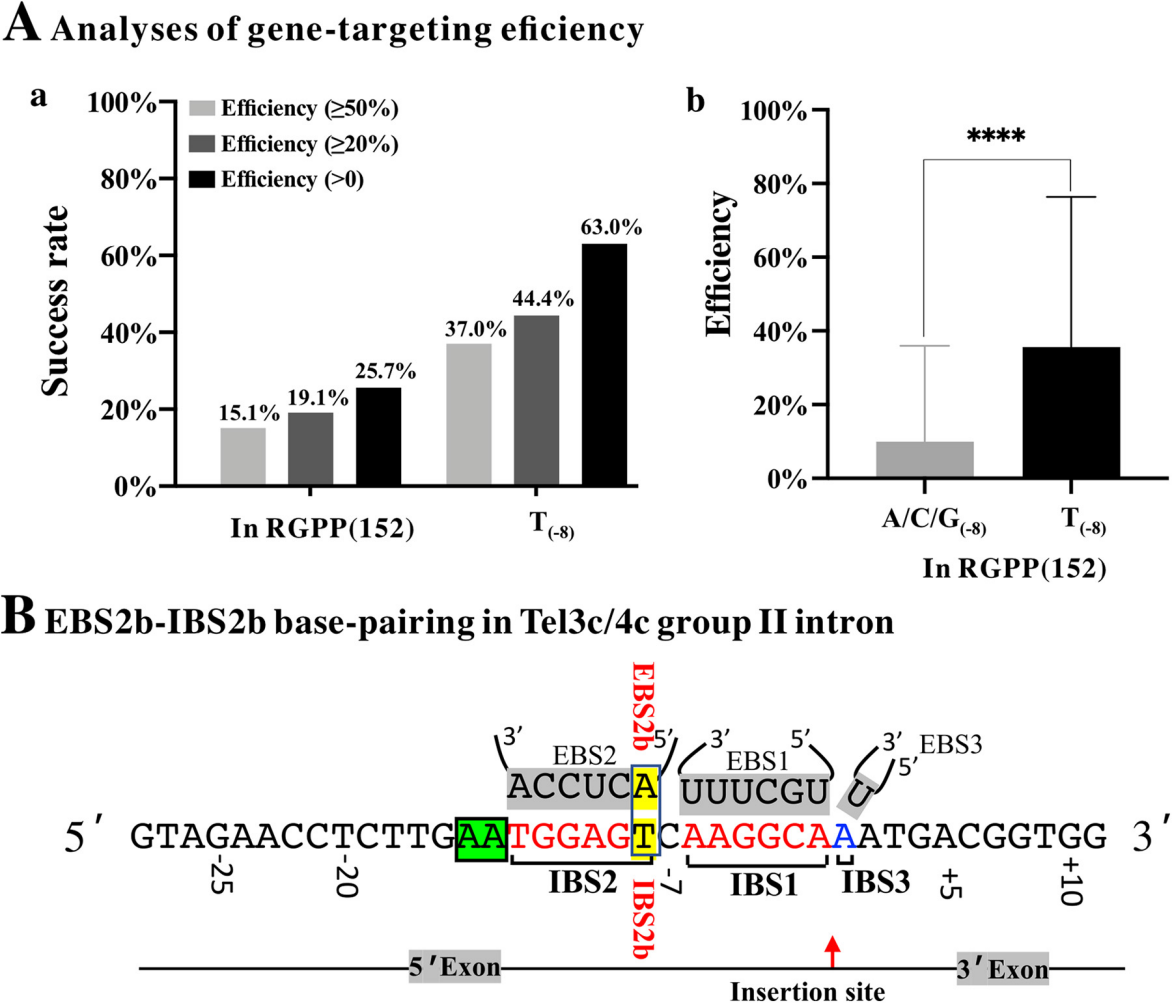
Gene-targeting efficiency analyses. (A) Gene-targeting success rate (a) and efficiency (b) in RGPP and selected successful gene-targeting events (“T” at -8 site, T-8). The different efficiencies (0, 20%, and 50%) refer to the ratio of gene-inactivated colonies to the total analyzed colonies at each targeting site. (B) EBS-IBS base-pairing for Tel3c/4c group II intron. The Mann-Whitney test was used to compare the differences between groups. The results were expressed as mean ± standard deviation, with a test level of a = 0.05, and *P* < 0.05 was statistically significant. ns, *P* > 0.1; *, *P* < 0.05; **, *P < *0.01; ***, *P < *0.001; ****, *P < *0.0001.

All 152 random plasmids were transformed into E. coli HMS174 (DE3) to analyze the target sites’ preference for TMT, and colony PCR was used to detect the successful insertion events. In the gene-targeting assay, we mainly focused on (i) the success rate of gene targeting, defined as the success of screening mutant strains among 24 random clones regardless of efficiency, which can meet the requirements of most gene inactivation experiments; (ii) gene-targeting efficiency = [(gene-inactivated colonies)/(total detected colonies)] × 100%, with higher efficiency indicating it is easier to obtain mutant strains, which also indicates that TMT is more effective. The results showed that most gene-targeting plasmids failed to generate gene inactivation mutants at the randomly selected gene-targeting sites (74.3%, 113/152, gene-targeting efficiency = 0). Only 39 plasmids successfully generated gene inactivation mutants at the expected position (25.7%, 39/152, targeting efficiency >0, Fig. 4A and Table S3). Among them, 29 plasmids had a ≥20% gene-targeting efficiency, and 23 plasmids had a ≥50% gene-targeting efficiency, accounting for 19.1% (29/152) and 15.1% (23/152) overall gene-targeting efficiencies in RGPP, respectively (Fig. 4Aa and Table S3).

Afterward, we analyzed the base composition of targeting sequences in 39 successful targeting events (Fig. 3B). We found that T at the -8 position (T_−8_) had a notably higher frequency of 63.0%, while the frequency of the other three bases (A, C, and G) were 19.5%, 19.6%, and 12.1%, respectively (Fig. 3B). In comparison, there was no distinguishable base preference at the -7 position. To better characterize these differences, online WebLogo (https://weblogo.berkeley.edu) was employed to analyze the base composition in the successful retrohoming events (Fig. 3C). As shown in Fig. 3C, the frequencies of T_(−8)_ were 43.6% (17/39), 41.4% (12/29), and 43.5% (10/23) in >0%, ≥20%, and ≥50% gene-targeting efficiency groups, respectively. In other words, the T_(−8)_ base had a higher frequency than the A, C, and G bases in successfully retrohoming groups of TMT. In contrast, no significant frequency difference was found in the -7 position in the same group (Fig. 3C). These results indicated that the TMT might have a recognition bias to T at the -8 position.

### The A_(−8)_/T_(−8)_ pairing improved the TMT targeting efficiency.

Base composition and WebLogo analysis showed that T_(−8)_ was the dominant base in the successful targeting events. The success rate of selected targets with T_(−8)_ was 63.0%, compared with 25.7% in the RGPP. In other words, the success rate increased 2.45-fold in the T_(−8)_ group. Taking gene-targeting efficiency into account, the success rate increased 2.32- and 2.45-fold at gene-targeting efficiency ≥20% and ≥50%, respectively (Fig. 4Aa). Then, we further compared the gene-targeting efficiency between the selected T_(−8)_ group and A/C/G_(−8)_ group. The result showed that the selected T_(−8)_ group had significantly higher gene-targeting efficiency than the A/C/G_(−8)_ group (Fig. 4Ab).

We further analyzed the EBS1 sequence (complementary to IBS2) of successful retrohoming targets and found an “A” base forming the Watson-Crick pairing with “T_(−8)_” in IBS2. Thereafter, we named these two bases EBS2b and IBS2b (Fig. 4B). The higher rate of successful retrohoming events seemed to be caused by the newly discovered EBS2b-IBS2b (A/T) pairing, which was previously considered inessential (13).

### Verification of EBS2b-IBS2b paring improved the gene-targeting efficiency of TMT.

**FIG 5:**
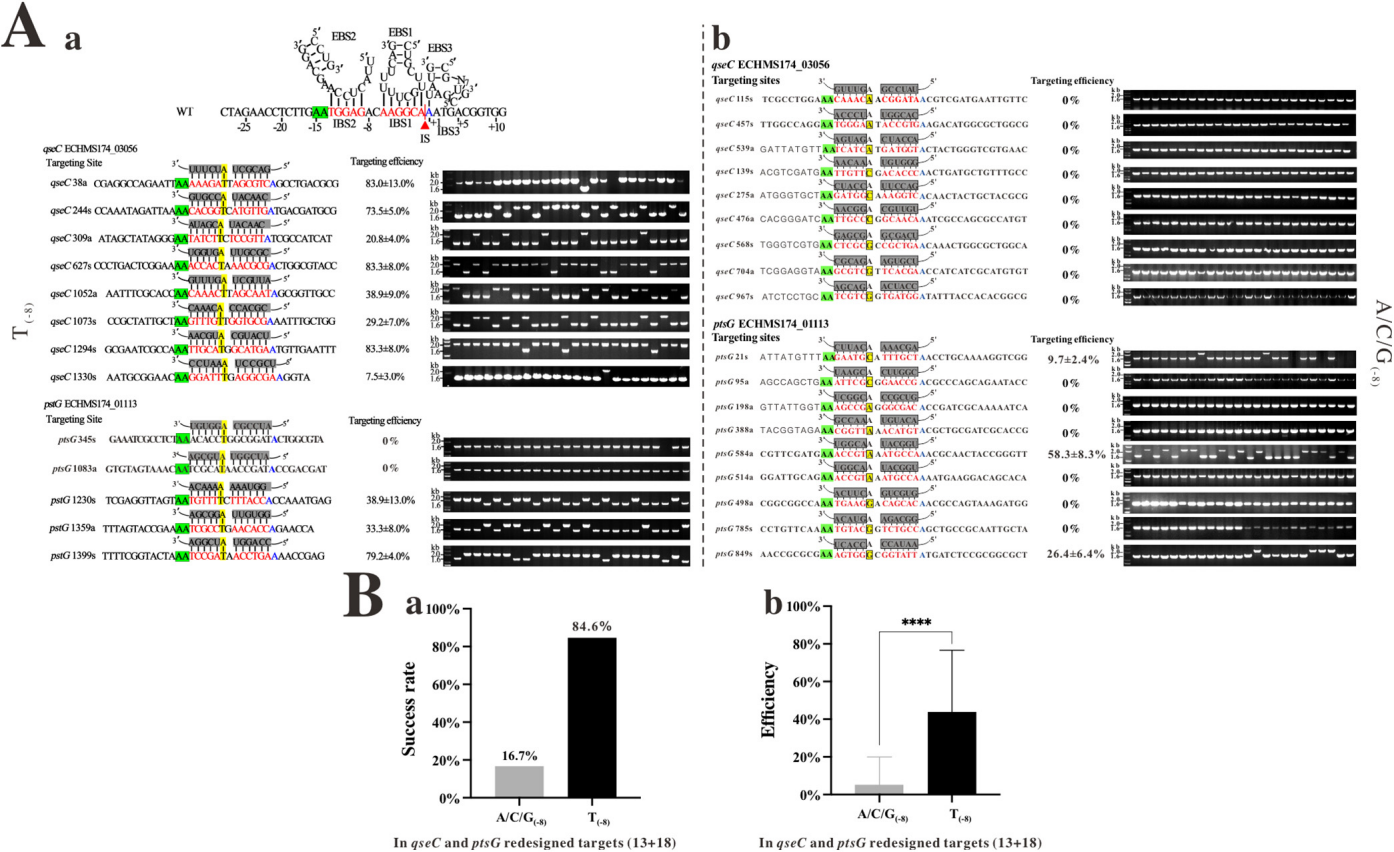
Gene targeting success rate and efficiency analyses of the redesigned targets. (A) Gene targeting efficiency in 13 + 18 redesigned targets, in which the “-8” site is forcibly set to “T” 13 plasmids T_(-8)_ group (a) and 18 plasmids A/C/G_(-8)_ group (b) are split by the dashed line. The left panel represents the targets' information (targeting sites and sequences) for both groups. The middle panel indicates the gene targeting efficiency. The right panel shows the diagnostic PCR results for each targeting site. (B) Comparing TMT targeting success rate (a) and efficiency (b) of EBS2b T_(-8)_ and A/C/G_(-8)_ groups in *qseC* and *ptsG* genes. The Kruskal-Wallis test was used to compare the differences between the two groups. The results were expressed as mean ± standard deviation, with a test level of a = 0.05, and *P* < 0.05 was statistically significant. ns, *P* > 0.1; *, *P* < 0.05; **, *P < *0.01; ***, *P < *0.001; ****, *P < *0.0001.

To further verify that the A/T pairing at EBS2b-IBS2b indeed improved the success rate and gene-targeting efficiency of TMT, we randomly redesigned 13 gene-targeting plasmids with T_(−8)_ and 18 gene-targeting plasmids with A/C/G_(−8)_ in IBS2b to inactive *qseC* and *ptsG* genes in *E.coli* HMS174 (DE3) genome (Tables S4 and S5). The results showed that 11 of the 13 plasmids (84.6%) were successfully inserted into the expected location (Fig. 5Aa and Table S6). Only 16.7% (3/18) of the A/C/G_(−8)_ group was able to get successful inactivation results in *qseC* and *ptsG* genes (Fig. 5Ab and Table S6). Compared with the A/C/G_(−8)_ group, the gene-targeting success rate of the T_(−8)_ group increased more than 5-fold (from 16.7% to 84.6%) (Fig. 5Ba). The gene-targeting efficiency of the T_(−8)_ group in *qseC* and *ptsG* genes was significantly higher than the A/C/G_(−8)_ group (*P* < 0.0001, Fig. 5Bb, Table S6). In summary, the success rate and gene-targeting efficiency with T_(−8)_ at IBS2b were significantly improved in both the RGPP (*P* < 0.0001, Fig. 4Ab) and redesigned gene targets (*P* < 0.0001, Fig. 5Bb).

### Effect of the unpaired and non-A/T paired of EBS2b-IBS2b on gene-targeting efficiency of TMT.

**FIG 6:**
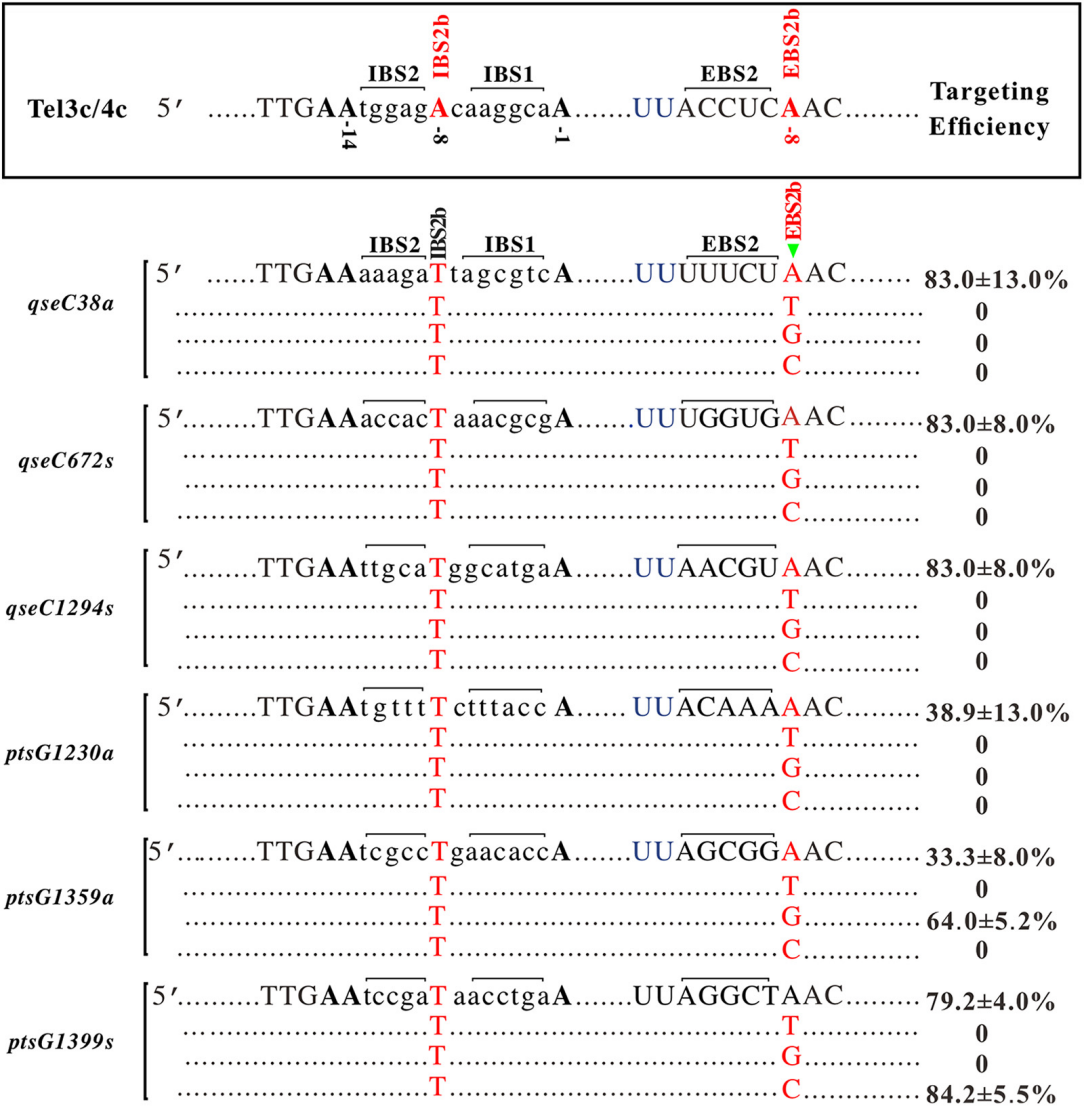
Comparison of gene-targeting efficiency with unpaired bases at the EBS2b site (T, G, C). Six targeting sites were randomly selected in *qseC* and *ptsG* genes, and three types of EBS2b-IBS2b unpaired conditions (T/T, G/T, and C/T) were constructed by point mutation method. The left panel indicates gene target sites, and the center panel indicates the recognition sequence of each target. The gene-targeting efficiency is listed in the right panel. The EBS2b-IBS2b pairing base was marked in red, and the solid green triangle indicated point mutation on the plasmids.

To further verify that the A/T pairing at the EBS2b-IBS2b site indeed improved the TMT’s success rate and gene-targeting efficiency, we randomly selected six confirmed targetable sites (*qseC38a*, *qseC627s*, *qseC1294s*, *ptsG1230a*, *ptsG1359a*, and *ptsG1399s*) on *qseC* and *ptsG* genes and designed three types of EBS2b-IBS2b unpaired conditions (T/T, G/T, and C/T, Table S7), by point mutation method, and 18 gene-targeting plasmids were constructed (Table S8). The results showed that only 2 of the 18 plasmids could obtain gene-inactivation mutants, and the targeting success rate decreased from 100% to 11.1% (2/18, Fig. 6, Table S9). These data further supported that T_(−8)_−A_(−8)_ pairing at EBS2b-IBS2b plays a vital role in the gene-targeting of TMT.

**FIG 7:**
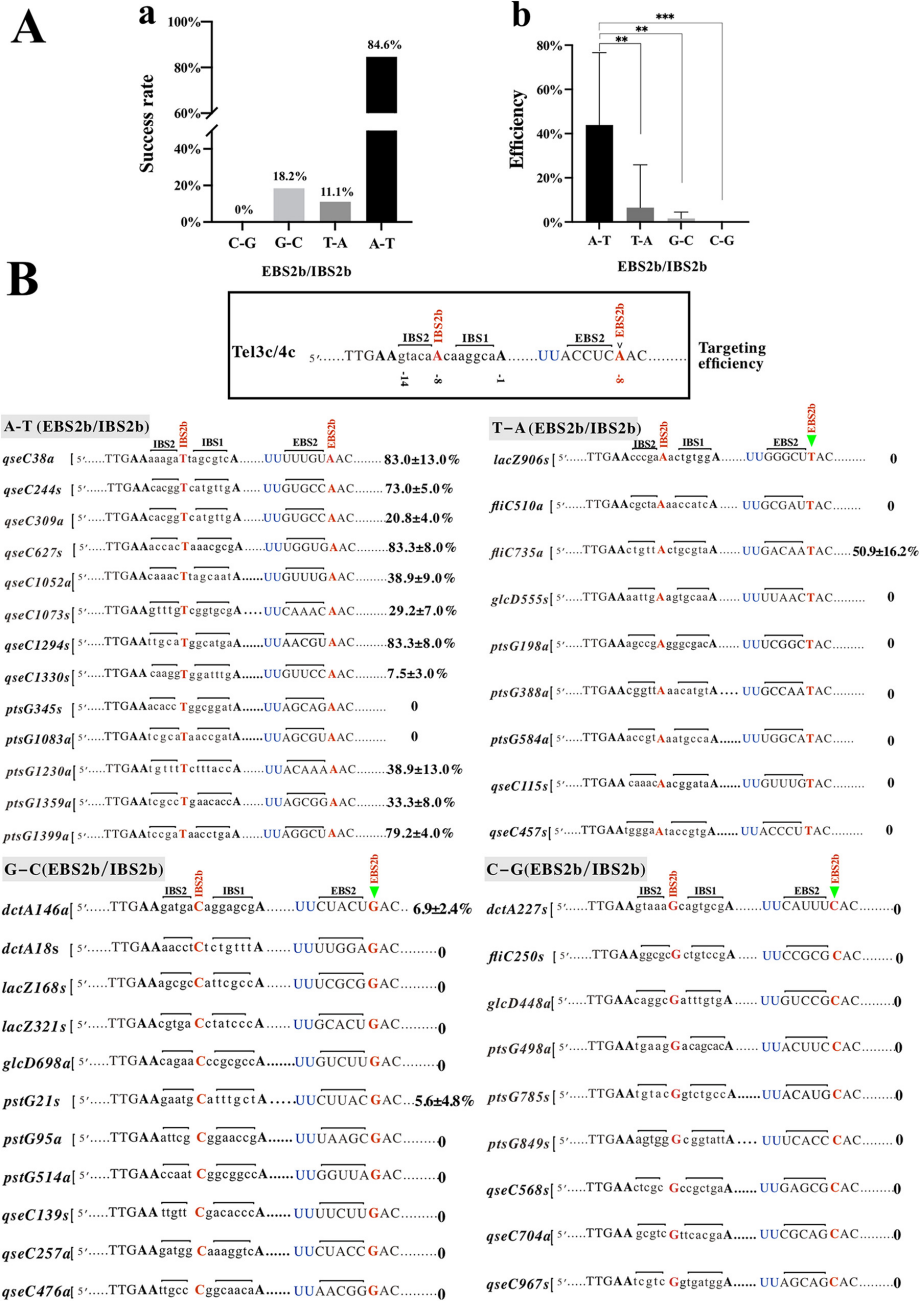
Comparison of gene-targeting success rate and efficiency with the non-A/T paired (T/A, G/C, and C/G) at the EBS2b-IBS2b site. (A) Comparison of targeting success rate (a) and efficiency (b) of C/G, G/C, T/A, and A/T pairing groups. (B) Targeting efficiency of four kinds of pairing at EBS2b/IBS2b site. For each base pairing pattern, the left panel indicates gene target sites, and the center panel indicates the recognition sequence of each target. The gene-targeting efficiency is listed in the right panel. The EBS2b-IBS2b pairing base is marked in red, and the solid green triangle indicates point mutation on the plasmids. The Kruskal-Wallis test was used to compare the differences between the two groups. The results are expressed as mean ± standard deviation, with a test level of a = 0.05, and *P* < 0.05 was statistically significant. ns, *P* > 0.1; *, *P* < 0.05; **, *P < *0.01; ***, *P < *0.001; ****, *P < *0.0001.

In addition, to analyze whether the non-A/T paired (T/A, G/C, and C/G) at EBS2b-IBS2b can also improve the TMT’s gene-targeting efficiency, we selected 29 sites (IBS2b was A/C/G on the genome) from the RGPP and *qseC* & *ptsG* redesigned targets, then point mutated its corresponding EBS2b to T/G/C on the plasmids to form T/A, G/C, and C/G pairing to generate 29 new gene-targeting plasmids targets (Table S10, Table S11). The new 29 targets are composed of 9 (T/A), 11 (G/C), and 9 (C/G) pairing plasmids (Fig. 7B). The gene-targeting results showed that the success rates were 11.1% (1/9), 18.2% (2/11), and 0% (0/9), in the case of EBS2b-IBS2b as T/A, G/C, and C/G, respectively (Fig. 7Aa). In the A/T pairing group, the gene-targeting efficiency was significantly higher than in the non-A/T paired groups (Fig. 7Ab, Table S12). Interestingly, these results indicated that the gene-targeting efficiency of TMT can only be enhanced when EBS2b-IBS2b were A/T pairing; any non-A/T paired (T/A, G/C, or C/G) significantly reduced the retrohoming efficiency of TMT, even when the EBS2b-IBS2b was T/A pairing.

### Computer-assisted TMT targets selection and primer design.

A Python-based algorithm, denoted TMT 1.0 (“TMT 1.0.py” file in Supplemental File 1), was developed to search potential target sites in any inputted gene sequences and design corresponding primers that abide by newly discovered high-efficiency rules. The TMT 1.0 is run with Python (Version 2.7.18; https://www.python.org/). Detailed procedures for using TMT 1.0 to design primers are illustrated in Fig. S1. All potential targets in either the sense (S) or antisense (A) strand of the target gene will be identified and listed. Strand A or S indicates the target sites were on the S or A strand. “Sites” indicates the position of insertion sites. “Targetron” showed the full-length sequence of target sites. The “(G+C) %” showed the GC contents of target sites. “Primers” were used as two pairs, X-IBS12 [X = gene name + insertion site + A or S strand]/TeI3c-UNV (5′-TAACGAGGCTTCTAGCG-3′); X-IBS 2s/IBS1a and the wild-type pHK-TT1A plasmid were used as the template (13). The PCR amplicons are assembled with the *BsiW*I-HF, and SpeI-HF linearized pHK-TT1A plasmid backbone to construct target-gene-specific targeting plasmid. The TMT 1.0 algorithm greatly facilitates the primer design of TMT, especially for beginners unfamiliar with TMT target searching and primer designing.

## DISCUSSION

In our previous study, we used a dual plasmid targeting system to evaluate the “retrohoming” efficiency of Tel3c/4c intron in E. coli. The intron-donor plasmid pACD2X-Tel3c/4c was employed to express the intron RNA and IEP protein. The recipient plasmid pBRR-3c contained a tetracycline resistance gene (Tet^R^) that could be activated when a retrohoming event occurred. The retrohoming efficiency was calculated as the ratio of (Tet^R^ + Amp^R^)/Amp^R^ colonies (13). The dual plasmid system is ideal for constructing a large target pool to elucidate the targeting rules and targeting efficiency of Tel3c/4c intron. However, when we applied this system to inactive genes in the genome of E. coli, we found that most gene-targeting plasmids failed to generate mutants. In other words, the success rate of TMT is low when used to inactivate genes in the genome of E. coli. Thus, in the present study, we constructed 152 gene-targeting sites in 4 genes to validate the influence of targeting sequence on the success rate (gene-targeting efficiency >0) and gene-targeting efficiency of TMT. Our results showed that the success rate of TMT increased 2.45- to 5.07-fold when EBS2b-IBS2b base paring (A_−8_/T_−8_) was presented (Fig. 4Aa and 5Ba). Furthermore, we designed a Python-based algorithm to predict gene targets bearing EBS2b-IBS2b base paring and to generate corresponding primers, which could greatly improve the feasibility of TMT in genome engineering. In summary, we have demonstrated that an additional Watson-Crick base pair (EBS2b-IBS2b) in TMT improves the gene-targeting efficiency (and success rate) of the E. coli genome. Our finding improved the success rate and gene-targeting efficiency of the existing TMT system, which will largely improve TMT’s feasibility and practicalities for genome editing in bacteria.

The gene-targeting efficiency of T_(−8)_-harboring targeting sequences compared to A/C/G_(−8)_ targeting sequences increased significantly in RGPP and the redesigned targets (Fig. 4Ab and 5Bb). However, two gene-targeting plasmids, *pstG*345s (5′-AAACACCTGGCGGATA-3′) and *pstG*1083a (5′-AATCGCATAACCGATA-3′), failed to generate any inactivation mutants (even after prolonging the induction duration at 48°C to 3 h and 48 colonies were screened). In addition, unpaired (T/T, G/T, and C/T, Fig. 6) and non-A/T paired conditions (T/A, G/C, and C/G, Fig. 7) of EBS2b-IBS2b also generated successful gene-targeting events, which were 11.1% (2/18) and 10.3% (3/29), even though less efficient. We speculated that these exceptions might be related to gene structure, gene context of target sites, or unknown reasons (30).

Base composition and targeting location may affect the TMT efficiency. To analyze the influence of GC content in TMT targeting sites on the efficiency of TMT targeting, 183 sites in RGPP and resigned targets were selected, and GC content in 53 successful targeting sites was analyzed. The results showed no significant correlation between GC content and gene-targeting efficiency. Only a nonsignificant negative correlation was exhibited, suggesting that higher GC content at the target site is associated with decreased efficiency (Fig. S2A). TMT targeting requires double-stranded DNA unwinding before identifying targeting sites via base pairing. DNA targeting sites with low GC content are more likely to be unwound than those with high GC content, which may be one of the reasons for the improved success rate. These results suggested that the higher GC content should be avoided when selecting the targeting sites (35% to 60% GC content is recommended).

To analyze the influence of targeting location on the efficiency of TMT, *fliC* (27 success sites, 44 failed sites) and *lacZ* (8 success sites, 58 failed sites) genes were selected to calculate the success rate in different regions (<1/3, 1/3 to 2/3, 2/3 to 3/3) of these two genes, and found that there was no significant correlation between the location and success rate (Fig. S2B). In addition, we also analyzed the influence of the DNA sense chain or antisense chain on the gene-targeting efficiency and found it had no significant influence on targeting efficiency either (Table S13).

Group II intron is a complex composed of intron RNA and a multifunctional intron-encoded protein (IEP), and the interaction between RNA and IEP, such as conformational change of IEP, or recognition and cleavage of nucleotide sequence, makes it difficult to obtain the spatial structure of group II intron (31, 32). In recent years, several high-resolution spatial structures of group II intron have been resolved due to the development of cryo-electron microscopy, such as Ll.ltrB (33), P.li.LSUI2 (34), Tel4h(3), and GsI-IIC (16, 35). The Tel4h and GsI-IIC belong to the thermophilic group II intron and are derived from Thermosynechococcus elongatus and Geobacillus stearothermophilus, respectively (3, 16, 35). These structures, especially the Tel4h and GsI-IIC, provide a good reference for the function research of Tel3c/4c intron. However, it should be emphasized that although Tel3c and Tel4h are derived from the same *T. elongatus*, they have different targeting rules (27). Additionally, a higher temperature is necessary for the thermophilic group II intron, which could promote double-strand DNA separation and help to increase the accessibility of DNA target sites, which is different from other mesophilic group II introns (27, 36). A variety of factors contribute to the different mechanisms of these amplified introns. Thus, to reveal the mechanism of how EBS2b-IBS2b paring improved the targeting efficiency, the three-dimensional structure of Tel3c/4c intron should be obtained in subsequent studies.

The Tel3c/4c is derived from thermophilic cyanobacterium *T. elongatus* and belongs to a thermophilic Group II introns class, which is active at 42°C to 48°C (13, 27). The optimal growth temperature of the E. coli HMS174 (DE3) strain is 37°C, and it can endure higher temperatures shortly. We performed the gene-targeting experiments at 48°C, in which the E. coli HMS174 (DE3) strain was under a heat shock environment. Thus, the TMT’s success rate and targeting efficiency might differ in thermophilic bacteria. Therefore, in future studies, the improved TMT system should also be tested in thermophilic microorganisms, such as Clostridium thermocellum or Bacillus stearothermophilus.

Group II intron plays a vital role in gene targeting; moreover, the reverse transcriptase, especially the heat-stabilized reverse transcriptase derived from thermophilic group II intron, has been found to have substantial potential in application in many fields, such as in gene editing, bacteria defene, biotechnology, next-generation sequencing, and others (1, 16, [Bibr B37][Bibr B38] 42). In conclusion, our research proved that the A_(−8)_/T_(−8)_ pairing at EBS2b-IBS2b sites significantly improved the gene-targeting efficiency (and success rate) of TMT, and the tailored TMT 1.0 algorithm greatly facilitated the targeting sites searching and its corresponding primers designing. Furthermore, an in-depth study of the structure, function and catalytic mechanism of thermophilic group II introns is suggested and will provide new clues and perspectives for the further application of group II introns.

## MATERIALS AND METHODS

### Strains, cultures, and growth conditions.

E. coli NEBExpress (NEB) was used for plasmid construction. Strains were grown in Luria-Bertani (LB) medium and cultured under aerobic conditions in a 37°C shaker, and 10 μg/mL chloramphenicol was supplemented when needed. The E. coli HMS174(DE3) (Invitrogen) was used for gene targeting analyses. It was grown in LB medium and cultured under aerobic conditions in a 37°C shaker, and 10 μg/mL chloramphenicol was supplemented when needed. The E. coli HMS174 (DE3) containing Thermotargetron (TMT) plasmid was cultured at 48°C to induce intron RNA expression, assemble, and fold into functional conformation when analyzing intron mobility and targeting efficiency. The targeting analysis and calculation section described the detailed induction, culture, and screening procedures.

### Construction of gene-targeting plasmids.

The genomic sequence of E. coli was used as the template to design targeting sequences and primers based on the previously discovered principles of Tel3c/4c (13, 27). The recognition sequences with the following characteristics A_(−15)_A_(−14)_nnnnnnnnnnnnnA_(+1)_ (“n” represents 13 arbitrary nucleotides, downstream of intron insertion site was determined as +1, and upstream was determined as −1) were selected as the targeting sites (Fig. 1Ba). Random gene targeting pool primers and redesigned gene targets primers were designed based on the targeting principle determined in our previous research and synthesized by Sangon Biotech (Shanghai, China) (Tables S1 and S4) (13). The SOEing PCR was used to amplify the gene-targeting fragments, and the T5 exonuclease-dependent assembly method (TEDA) was used to assemble the gene-targeting fragments with *Bsi*WI-HF, and SpeI-HF linearized pHK-TT1A plasmid backbone (Table S2 and S5) (43). In addition, to analyze the targeting efficiency of EBS2b-IBS2b unpaired conditions (T/T, G/T, C/T) and non-A/T paired (T/A, G/C, C/G), specific mutation primers were also designed according to the TMT recognition rules to construct the point mutation targeting plasmids (Table S7 and S10). All targeting primers and plasmids are listed in supplemental tables.

### Gene-targeting analyses.

The E. coli HMS174(DE3) was transformed with targeting plasmid, spread onto LB plates containing 10 μg/mL chloramphenicol, and incubated in a 37°C incubator overnight. The single colony was picked and inoculated into the LB broth medium containing 10 μg/mL chloramphenicol and cultured in a 37°C shaker at 180 rpm until it reached the logarithmic phase. Then, the 1-mL cultures were transferred to a 1.5-mL centrifuge. The transferred cultures were shocked at 48°C for 1 h to activate the Tel3c/4c intron, then serial diluted (10^−1^ to 10^−8^) and spread onto LB plates containing 10 μg/mL chloramphenicol and incubated at 37°C overnight. Finally, colony PCR was used to determine the gene-targeting efficiency. For each plasmid, 24 colonies were randomly selected for PCR verification, and the proportion of positive colonies was calculated. If all 24 colonies were negative, the target efficiency was denoted as zero. The targeting efficiency is calculated as follows: gene-targeting efficiency = [(gene-inactivated colonies)/(total detected colonies)] × 100%.

### A Python-based algorithm to determine potential targeting sites and design TMT primers.

A Python-based algorithm named “TMT 1.0.py” (Supplemental File 1), was designed to find potential TMT gene-targeting sites in any given gene sequence and design its corresponding targeting primers. TMT 1.0 is run with Python (Version 2.7.18; https://www.python.org/). Detailed procedures to design primers with TMT 1.0 are noted in Fig. S1. Briefly, all the targets on the sense (S) and antisense (A) chains of the input gene and its corresponding primers are screened and listed by the algorithm when the DNA sequence of the target gene was input into the appropriate position indicated by the algorithm. The target sites’ CG% contents (GC%) and the Tm value (Tm) are also listed.

### Statistical methods.

Prism 8 (Version 8.2.1) was used for statistical analysis. Mann-Whitney, Kruskal-Wallis, chi-square tests, or Spearman correlation were used to compare differences between groups when appropriate (indicated in the figure legends), with a test level of a = 0.05, and *P* < 0.05 was statistically significant. ns, *P* > 0.1; *, *P* < 0.05; **, *P* < 0.01; ***, *P < *0.001; ****, *P < *0.0001.

### Data availability.

All original data and biological resources (such as plasmids and strains) are available upon email request sent to Wei Hong (hongwei@gmc.edu.cn). No high-throughput sequencing or other large data sets were generated in the present study.
