# Supplementary material for: A New EBS2b-IBS2b Base Paring (A−8/T−8) Improved the Gene-Targeting Efficiency of Thermotargetron in Escherichia coli
Source: Microbiol Spectr. 2023 Feb 21;11(2):e03159-22. doi: 10.1128/spectrum.03159-22 (PMC10100991; doi:10.1128/spectrum.03159-22)
Supplement: Supplemental file 1 — Supplemental material. Download spectrum.03159-22-s0001.pdf, PDF file, 3.2 MB [file spectrum.03159-22-s0001.pdf]

Supplementary figures and legends

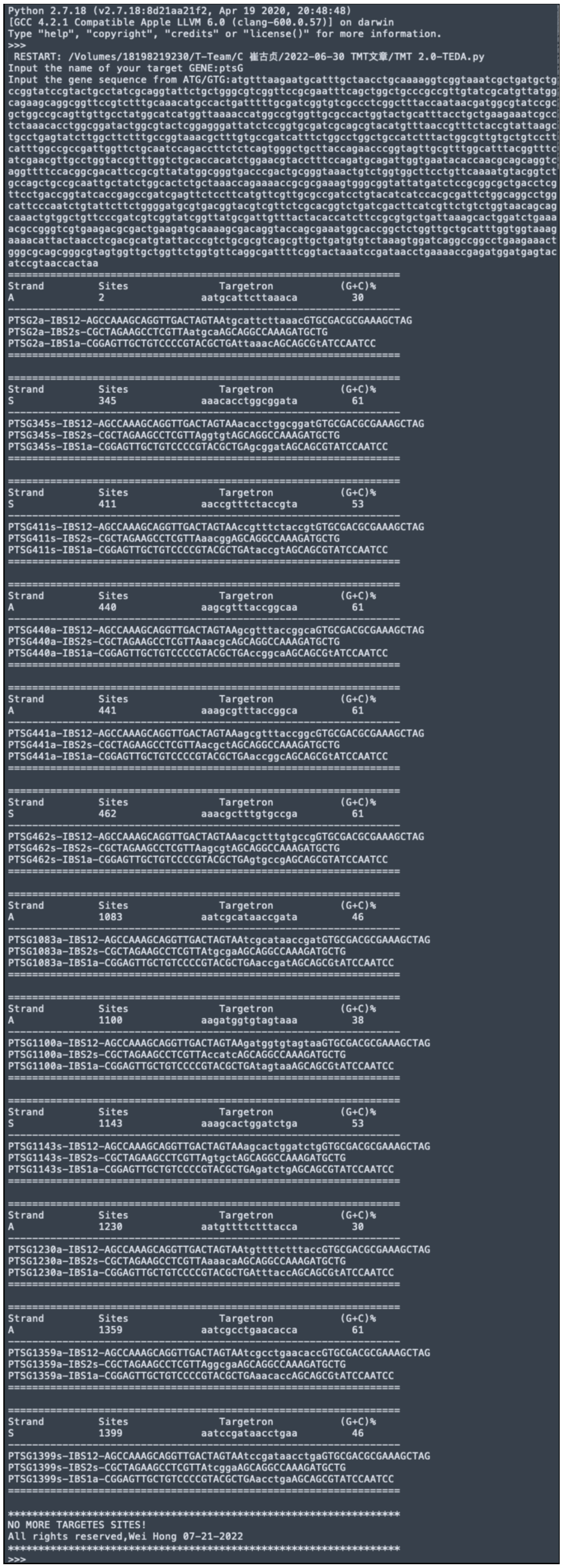

```
Python 2.7.18 (v2.7.18:8d21aa21f2, Apr 19 2020, 20:48:48)
[GCC 4.2.1 Compatible Apple LLVM 6.0 (clang-600.0.57)] on darwin
Type "help", "copyright", "credits" or "license()" for more information.
>>>
 RESTART: /Volumes/18198219230/T-Team/C 崔古贞/2022-06-30 TMT文章/TMT 2.0-TEDA.py
Input the name of your target GENE:ptsG
Input the gene sequence from ATG/GTG:atgtttaagaatgcatttgctaacctgcaaaaggtcggtaaatcgctgatgctg
ccggtatccgtactgcctatcgcaggtattctgctgggcgtcggttccgcgaatttcagctggctgcccgccgttgtatcgcatgttatgg
cagaagcaggcggttccgtctttgcaaacatgccactgattttgcgatcggtgtcgccctcggctttaccaataacgatggcgtatccgc
gctggccgcagttgttgcctatggcatcatggttaaaaccatggccgtggttgcgccactggtactgcatttacctgctgaagaaatcgcc
tctaaacacctggcggatactggcgtactcggagggattatctccggtgcgatcgcagcgtacatgtttaaccgtttctaccgtattaagc
tgcctgagtatcttggcttctttgccggtaaacgctttgtgccgatcatttctggcctggctgccatctttactggcgttgtgctgtcctt
catttggccgccgattggttctgcaatccagaccttctctcagtgggctgcttaccagaacccggtagttgcgtttggcatttacggtttc
atcgaacgttgcctggtaccgtttggtctgcaccacatctggaacgtacctttccagatgcagattggtgaatacaccaacgcagcaggtc
aggttttccacggcgacattccgcgttatatggcgggtgacccgactgcgggtaaactgtctggtggcttcctgttcaaaatgtacggtct
gccagctgccgcaattgctatctggcactctgctaaaccagaaaaccgcgcgaaagtgggcggtattatgatctccgcggcgctgacctcg
ttcctgaccggtatcaccgagccgatcgagttctccttcatgttcgttgcgccgatcctgtacatcatccacgcgattctggcaggcctgg
cattcccaatctgtattcttctggggatgcgtgacggtacgtcgttctcgcacggtctgatcgacttcatcgttctgtctggtaacagcag
caaactgtggctgttcccgatcgtcggtatcggttatgcgattgtttactacaccatcttccgcgtgctgattaaagcactggatctgaaa
acgccgggtcgtgaagacgcgactgaagatgcaaaagcgacaggtaccagcgaaatggcaccggctctggttgctgcatttggtggtaaag
aaaacattactaacctcgacgcatgtattacccgtctgcgcgtcagcgttgctgatgtgtctaaagtggatcaggccggcctgaagaaact
gggcgcagcgggcgtagtggttgctggttctggtgttcaggcgattttcggtactaaatccgataacctgaaaaccgagatggatgagtac
atccgtaaccactaa
===================================================================
Strand          Sites               Targetron           (G+C)%
A               2                   aatgcattcttaaaca      30
-------------------------------------------------------------------
PTSG2a-IBS12-AGCCAAAGCAGGTTGACTAGTAAtgcattcttaaacGTGCGACGCGAAAGCTAG
PTSG2a-IBS2s-CGCTAGAAGCCTCGTTAatgcaAGCAGGCCAAAGATGCTG
PTSG2a-IBS1a-CGGAGTTGCTGTCCCCGTACGCTGAttaaacAGCAGCGtATCCAATCC
===================================================================

===================================================================
Strand          Sites               Targetron           (G+C)%
S               345                 aaacacctggcggata      61
-------------------------------------------------------------------
PTSG345s-IBS12-AGCCAAAGCAGGTTGACTAGTAAacacctggcggatGTGCGACGCGAAAGCTAG
PTSG345s-IBS2s-CGCTAGAAGCCTCGTTAggtgtAGCAGGCCAAAGATGCTG
PTSG345s-IBS1a-CGGAGTTGCTGTCCCCGTACGCTGAgcggatAGCAGCGTATCCAATCC
===================================================================

===================================================================
Strand          Sites               Targetron           (G+C)%
S               411                 aaccgtttctaccgta      53
-------------------------------------------------------------------
PTSG411s-IBS12-AGCCAAAGCAGGTTGACTAGTAAccgtttctaccgtGTGCGACGCGAAAGCTAG
PTSG411s-IBS2s-CGCTAGAAGCCTCGTTAaacggAGCAGGCCAAAGATGCTG
PTSG411s-IBS1a-CGGAGTTGCTGTCCCCGTACGCTGAtaccgtAGCAGCGTATCCAATCC
===================================================================

===================================================================
Strand          Sites               Targetron           (G+C)%
A               440                 aagcgtttaccggcaa      61
-------------------------------------------------------------------
PTSG440a-IBS12-AGCCAAAGCAGGTTGACTAGTAAgcgtttaccggcaGTGCGACGCGAAAGCTAG
PTSG440a-IBS2s-CGCTAGAAGCCTCGTTAaacgcAGCAGGCCAAAGATGCTG
PTSG440a-IBS1a-CGGAGTTGCTGTCCCCGTACGCTGAccggcaAGCAGCGtATCCAATCC
===================================================================

===================================================================
Strand          Sites               Targetron           (G+C)%
A               441                 aaagcgtttaccggca      61
-------------------------------------------------------------------
PTSG441a-IBS12-AGCCAAAGCAGGTTGACTAGTAAagcgtttaccggcGTGCGACGCGAAAGCTAG
PTSG441a-IBS2s-CGCTAGAAGCCTCGTTAacgctAGCAGGCCAAAGATGCTG
PTSG441a-IBS1a-CGGAGTTGCTGTCCCCGTACGCTGAaccggcAGCAGCGtATCCAATCC
===================================================================

===================================================================
Strand          Sites               Targetron           (G+C)%
S               462                 aaacgctttgtgccga      61
-------------------------------------------------------------------
PTSG462s-IBS12-AGCCAAAGCAGGTTGACTAGTAAacgctttgtgccgGTGCGACGCGAAAGCTAG
PTSG462s-IBS2s-CGCTAGAAGCCTCGTTAagcgtAGCAGGCCAAAGATGCTG
PTSG462s-IBS1a-CGGAGTTGCTGTCCCCGTACGCTGAgtgccgAGCAGCGTATCCAATCC
===================================================================

===================================================================
Strand          Sites               Targetron           (G+C)%
A               1083                aatcgcataaccgata      46
-------------------------------------------------------------------
PTSG1083a-IBS12-AGCCAAAGCAGGTTGACTAGTAAtcgcataaccgatGTGCGACGCGAAAGCTAG
PTSG1083a-IBS2s-CGCTAGAAGCCTCGTTAtgcgaAGCAGGCCAAAGATGCTG
PTSG1083a-IBS1a-CGGAGTTGCTGTCCCCGTACGCTGAaccgatAGCAGCGtATCCAATCC
===================================================================

===================================================================
Strand          Sites               Targetron           (G+C)%
A               1100                aagatggtgtagtaaa      38
-------------------------------------------------------------------
PTSG1100a-IBS12-AGCCAAAGCAGGTTGACTAGTAAgatggtgtagtaaGTGCGACGCGAAAGCTAG
PTSG1100a-IBS2s-CGCTAGAAGCCTCGTTAccatcAGCAGGCCAAAGATGCTG
PTSG1100a-IBS1a-CGGAGTTGCTGTCCCCGTACGCTGAtagtaaAGCAGCGtATCCAATCC
===================================================================

===================================================================
Strand          Sites               Targetron           (G+C)%
S               1143                aaagcactggatctga      53
-------------------------------------------------------------------
PTSG1143s-IBS12-AGCCAAAGCAGGTTGACTAGTAAagcactggatctgGTGCGACGCGAAAGCTAG
PTSG1143s-IBS2s-CGCTAGAAGCCTCGTTAgtgctAGCAGGCCAAAGATGCTG
PTSG1143s-IBS1a-CGGAGTTGCTGTCCCCGTACGCTGAgatctgAGCAGCGTATCCAATCC
===================================================================

===================================================================
Strand          Sites               Targetron           (G+C)%
A               1230                aatgttttctttacca      30
-------------------------------------------------------------------
PTSG1230a-IBS12-AGCCAAAGCAGGTTGACTAGTAAtgttttctttaccGTGCGACGCGAAAGCTAG
PTSG1230a-IBS2s-CGCTAGAAGCCTCGTTAaaacaAGCAGGCCAAAGATGCTG
PTSG1230a-IBS1a-CGGAGTTGCTGTCCCCGTACGCTGAtttaccAGCAGCGtATCCAATCC
===================================================================

===================================================================
Strand          Sites               Targetron           (G+C)%
A               1359                aatcgcctgaacacca      61
-------------------------------------------------------------------
PTSG1359a-IBS12-AGCCAAAGCAGGTTGACTAGTAAtcgcctgaacaccGTGCGACGCGAAAGCTAG
PTSG1359a-IBS2s-CGCTAGAAGCCTCGTTAggcgaAGCAGGCCAAAGATGCTG
PTSG1359a-IBS1a-CGGAGTTGCTGTCCCCGTACGCTGAaacaccAGCAGCGtATCCAATCC
===================================================================

===================================================================
Strand          Sites               Targetron           (G+C)%
S               1399                aatccgataacctgaa      46
-------------------------------------------------------------------
PTSG1399s-IBS12-AGCCAAAGCAGGTTGACTAGTAAtccgataacctgaGTGCGACGCGAAAGCTAG
PTSG1399s-IBS2s-CGCTAGAAGCCTCGTTAtcggaAGCAGGCCAAAGATGCTG
PTSG1399s-IBS1a-CGGAGTTGCTGTCCCCGTACGCTGAacctgaAGCAGCGTATCCAATCC
===================================================================

*******************************************************************
NO MORE TARGETES SITES!
All rights reserved,Wei Hong 07-21-2022
*******************************************************************
>>>
```

Figure S1 Design primers using the TMT 1.0 for constructing the gene-targeting plasmid (taking the *ptsG* gene as an example).

Python 2.7.18 (https://www.python.org/) software should be pre-installed. The *ptsG* gene sequence (Gene ID: 945651) is downloaded from NCBI. The Thermotargetron 1.0 program can be launched by double-clicking the "TMT 1.0.py" file (Supplementary file 1). The *ptsG* gene sequence can be input into the appropriate blank as indicated by the algorithm. The TMT 1.0 program will list all the suitable insertion sites, and targeting primers fit TMT's newly discovered gene-targeting roles. To each set of primers, the strand 'A' and 'S' represents the 'antisense' and 'sense' chain of the genome, respectively; 'Sites' represents the insertion site of intron RNA in the *ptsG* gene; 'Targetron' sequence represents the recognition site of group II intron, including IBS2, IBS2b, IBS1 sites; '(G+C)%' represents GC percentage; the three primers given for each insertion site are specific primers and together with universal primer to construct specific targeting plasmids. The method of constructing targeting plasmid is described in the "Materials and Methods" section.

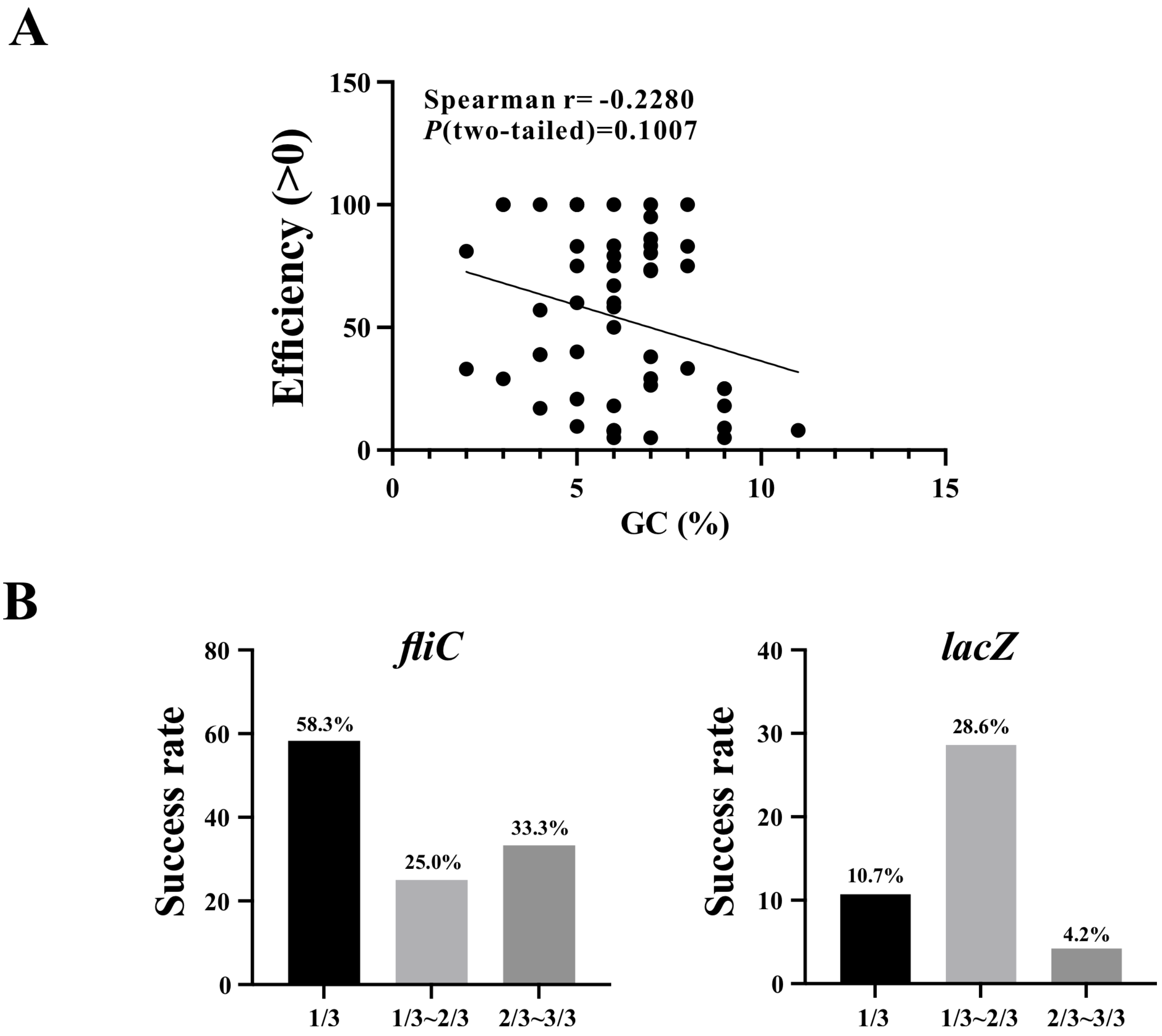

Figure S2 The effect of GC content and targeting location on TMT targeting efficiency.

(A) The correlation of GC content and TMT gene-targeting efficiency. (B) The effect of targeting on TMT success rate in *fliC* and *lacZ* genes. The correlation of GC content and TMT gene-targeting efficiency. The Spearman correlation was used to compute r between GC content and gene-targeting efficiency, and the results were expressed as the P value, with a test level of a =0.05. *P* = 0.1007 showed no significant correlation. *, *P*<0.05; **, *P*<0.01; ***, *P*<0.001; ****, *P*<0.0001.

Supplemental Tables

Table S1 Primers used in RGPP construction.

Primers	Sequences (5′-3′) *	Note
TeI3c-UNV	TAACGAGGCTTCTAGCG	Universal primer
*fliC*36sIBS12	AGCCAAAGCAGGTTGACTAGTAAcagcctctcgctgGTGCGACGCGAAAGCTAG	
*fliC*36sEBS2s	CGCTAGAAGCCTCGTTAggctgAGCAGGCCAAAGATGCTG	Targeting *fliC*36s
*fliC*36sEBS1a	CGGAGTTGCTGTCCCCGTACGCTGAtcgctgAGCAGCGTATCCAATCC	
*fliC*60sIBS12	AGCCAAAGCAGGTTGACTAGTAAtaatatcaacaagGTGCGACGCGAAAGCTAG	
*fliC*60sEBS2s	CGCTAGAAGCCTCGTTAtattaAGCAGGCCAAAGATGCTG	Targeting *fliC*60s
*fliC*60sEBS1a	CGGAGTTGCTGTCCCCGTACGCTGAaacaagAGCAGCGTATCCAATCC	
*fliC*67aIBS12	AGCCAAAGCAGGTTGACTAGTAActcgacagcgcagGTGCGACGCGAAAGCTAG	
*fliC*67aEBS2s	CGCTAGAAGCCTCGTTAtcgagAGCAGGCCAAAGATGCTG	Targeting *fliC*67a
*fliC*67aEBS1a	CGGAGTTGCTGTCCCCGTACGCTGAgcgcagAGCAGCGTATCCAATCC	
*fliC*250sIBS12	AGCCAAAGCAGGTTGACTAGTAAggcgcgctgtccgGTGCGACGCGAAAGCTAG	
*fliC*250sEBS2s	CGCTAGAAGCCTCGTTAgcgccAGCAGGCCAAAGATGCTG	Targeting *fliC*250s
*fliC*250sEBS1a	CGGAGTTGCTGTCCCCGTACGCTGAtgtccgAGCAGCGTATCCAATCC	
*fliC*266sIBS12	AGCCAAAGCAGGTTGACTAGTAAtcaacaacaacttGTGCGACGCGAAAGCTAG	
*fliC*266sEBS2s	CGCTAGAAGCCTCGTTAgttgaAGCAGGCCAAAGATGCTG	Targeting *fliC*266s
*fliC*266sEBS1a	CGGAGTTGCTGTCCCCGTACGCTGAcaacttAGCAGCGTATCCAATCC	
*fliC*420sIBS12	AGCCAAAGCAGGTTGACTAGTAAcgtgctggcaaaaGTGCGACGCGAAAGCTAG	
*fliC*420sEBS2s	CGCTAGAAGCCTCGTTAgcacgAGCAGGCCAAAGATGCTG	Targeting *fliC*420s
*fliC*420sEBS1a	CGGAGTTGCTGTCCCCGTACGCTGAgcaaaaAGCAGCGTATCCAATCC	
*fliC*432sIBS12	AGCCAAAGCAGGTTGACTAGTAAaaatggctccatgGTGCGACGCGAAAGCTAG	
*fliC*432sEBS2s	CGCTAGAAGCCTCGTTAcatttAGCAGGCCAAAGATGCTG	Targeting *fliC*432s
*fliC*432sEBS1a	CGGAGTTGCTGTCCCCGTACGCTGAtccatgAGCAGCGTATCCAATCC	
*fliC*434sIBS12	AGCCAAAGCAGGTTGACTAGTAAatggctccatgaaGTGCGACGCGAAAGCTAG	
*fliC*434sEBS2s	CGCTAGAAGCCTCGTTAgccatAGCAGGCCAAAGATGCTG	Targeting *fliC*434s
*fliC*434sEBS1a	CGGAGTTGCTGTCCCCGTACGCTGAcatgaaAGCAGCGTATCCAATCC	
*fliC*435sIBS12	AGCCAAAGCAGGTTGACTAGTAAtggctccatgaaaGTGCGACGCGAAAGCTAG	
*fliC*435sEBS2s	CGCTAGAAGCCTCGTTAagccaAGCAGGCCAAAGATGCTG	Targeting *fliC*435s
*fliC*435sEBS1a	CGGAGTTGCTGTCCCCGTACGCTGAatgaaaAGCAGCGTATCCAATCC	
*fliC*449sIBS12	AGCCAAAGCAGGTTGACTAGTAAtccaggttggcgcGTGCGACGCGAAAGCTAG	
*fliC*449sEBS2s	CGCTAGAAGCCTCGTTActggaAGCAGGCCAAAGATGCTG	Targeting *fliC*449s
*fliC*449sEBS1a	CGGAGTTGCTGTCCCCGTACGCTGAtggcgcAGCAGCGTATCCAATCC	
*fliC*465sIBS12	AGCCAAAGCAGGTTGACTAGTAAtgataaccagactGTGCGACGCGAAAGCTAG	
*fliC*465sEBS2s	CGCTAGAAGCCTCGTTAtatcaAGCAGGCCAAAGATGCTG	Targeting *fliC*465s
*fliC*465sEBS1a	CGGAGTTGCTGTCCCCGTACGCTGAcagactAGCAGCGTATCCAATCC	
*fliC*489aIBS12	AGCCAAAGCAGGTTGACTAGTAAgagttttagcatcGTGCGACGCGAAAGCTAG	
*fliC*489aEBS2s	CGCTAGAAGCCTCGTTAaactcAGCAGGCCAAAGATGCTG	Targeting *fliC*489a
*fliC*489aEBS1a	CGGAGTTGCTGTCCCCGTACGCTGAagcatcAGCAGCGtATCCAATCC	
*fliC*495aIBS12	AGCCAAAGCAGGTTGACTAGTAAggccaagagttttGTGCGACGCGAAAGCTAG	
*fliC*495aEBS2s	CGCTAGAAGCCTCGTTAtggccAGCAGGCCAAAGATGCTG	Targeting *fliC*495a
*fliC*495aEBS1a	CGGAGTTGCTGTCCCCGTACGCTGAagttttAGCAGCGtATCCAATCC	
*fliC*495sIBS12	AGCCAAAGCAGGTTGACTAGTAAgcagattgatgctGTGCGACGCGAAAGCTAG	Targeting *fliC*495s

*fliC*495sEBS2s	CGCTAGAAGCCTCGTTAtctgcAGCAGGCCAAAGATGCTG	
*fliC*495sEBS1a	CGGAGTTGCTGTCCCCGTACGCTGAgatgctAGCAGCGTATCCAATCC	
*fliC*504aIBS12	AGCCAAAGCAGGTTGACTAGTAAaaccatcaaggccGTGCGACGCGAAAGCTAG	
*fliC*504aEBS2s	CGCTAGAAGCCTCGTTAtggttAGCAGGCCAAAGATGCTG	Targeting *fliC*504a
*fliC*504aEBS1a	CGGAGTTGCTGTCCCCGTACGCTGAaaggccAGCAGCGTATCCAATCC	
*fliC*510aIBS12	AGCCAAAGCAGGTTGACTAGTAAcgctaaaaccatcGTGCGACGCGAAAGCTAG	
*fliC*510aEBS2s	CGCTAGAAGCCTCGTTAtagcgAGCAGGCCAAAGATGCTG	Targeting *fliC*510a
*fliC*510aEBS1a	CGGAGTTGCTGTCCCCGTACGCTGAaccatcAGCAGCGTATCCAATCC	
*fliC*511sIBS12	AGCCAAAGCAGGTTGACTAGTAAactcttggccttgGTGCGACGCGAAAGCTAG	
*fliC*511sEBS2s	CGCTAGAAGCCTCGTTAagagtAGCAGGCCAAAGATGCTG	Targeting *fliC*511s
*fliC*511sEBS1a	CGGAGTTGCTGTCCCCGTACGCTGAgccttgAGCAGCGTATCCAATCC	
*fliC*543sIBS12	AGCCAAAGCAGGTTGACTAGTAAtaacgatacagttGTGCGACGCGAAAGCTAG	
*fliC*543sEBS2s	CGCTAGAAGCCTCGTTAcgttaAGCAGGCCAAAGATGCTG	Targeting *fliC*543s
*fliC*543sEBS1a	CGGAGTTGCTGTCCCCGTACGCTGAacagttAGCAGCGTATCCAATCC	
*fliC*600aIBS12	AGCCAAAGCAGGTTGACTAGTAAgggtaattccagtGTGCGACGCGAAAGCTAG	
*fliC*600aEBS2s	CGCTAGAAGCCTCGTTAtacccAGCAGGCCAAAGATGCTG	Targeting *fliC*600a
*fliC*600aEBS1a	CGGAGTTGCTGTCCCCGTACGCTGAtccagtAGCAGCGTATCCAATCC	
*fliC*600sIBS12	AGCCAAAGCAGGTTGACTAGTAAcaatattaaacttGTGCGACGCGAAAGCTAG	
*fliC*600sEBS2s	CGCTAGAAGCCTCGTTAtattgAGCAGGCCAAAGATGCTG	Targeting *fliC*600s
*fliC*600sEBS1a	CGGAGTTGCTGTCCCCGTACGCTGAaaacttAGCAGCGTATCCAATCC	
*fliC*609sIBS12	AGCCAAAGCAGGTTGACTAGTAAacttactggaattGTGCGACGCGAAAGCTAG	
*fliC*609sEBS2s	CGCTAGAAGCCTCGTTAtaagtAGCAGGCCAAAGATGCTG	Targeting *fliC*609s
*fliC*609sEBS1a	CGGAGTTGCTGTCCCCGTACGCTGAggaattAGCAGCGTATCCAATCC	
*fliC*648aIBS12	AGCCAAAGCAGGTTGACTAGTAAttgaagctgggttGTGCGACGCGAAAGCTAG	
*fliC*648aEBS2s	CGCTAGAAGCCTCGTTAttcaaAGCAGGCCAAAGATGCTG	Targeting *fliC*648a
*fliC*648aEBS1a	CGGAGTTGCTGTCCCCGTACGCTGAtgggttAGCAGCGTATCCAATCC	
*fliC*657aIBS12	AGCCAAAGCAGGTTGACTAGTAAcaccctcaattgaGTGCGACGCGAAAGCTAG	
*fliC*657aEBS2s	CGCTAGAAGCCTCGTTAgggtgAGCAGGCCAAAGATGCTG	Targeting *fliC*657a
*fliC*657aEBS1a	CGGAGTTGCTGTCCCCGTACGCTGAaattgaAGCAGCGTATCCAATCC	
*fliC*658aIBS12	AGCCAAAGCAGGTTGACTAGTAAacaccctcaattgGTGCGACGCGAAAGCTAG	
*fliC*658aEBS2s	CGCTAGAAGCCTCGTTAggtgtAGCAGGCCAAAGATGCTG	Targeting *fliC*658a
*fliC*658aEBS1a	CGGAGTTGCTGTCCCCGTACGCTGAcaattgAGCAGCGTATCCAATCC	
*fliC*659sIBS12	AGCCAAAGCAGGTTGACTAGTAActaacccagcttcGTGCGACGCGAAAGCTAG	
*fliC*659sEBS2s	CGCTAGAAGCCTCGTTAgttagAGCAGGCCAAAGATGCTG	Targeting *fliC*659s
*fliC*659sEBS1a	CGGAGTTGCTGTCCCCGTACGCTGAagcttcAGCAGCGTATCCAATCC	
*fliC*702sIBS12	AGCCAAAGCAGGTTGACTAGTAAtgattactatgcgGTGCGACGCGAAAGCTAG	
*fliC*702sEBS2s	CGCTAGAAGCCTCGTTAaatcaAGCAGGCCAAAGATGCTG	Targeting *fliC*702s
*fliC*702sEBS1a	CGGAGTTGCTGTCCCCGTACGCTGAtatgcgAGCAGCGTATCCAATCC	
*fliC*718sIBS12	AGCCAAAGCAGGTTGACTAGTAAatcaccggtggtgGTGCGACGCGAAAGCTAG	
*fliC*718sEBS2s	CGCTAGAAGCCTCGTTAgtgatAGCAGGCCAAAGATGCTG	Targeting *fliC*718s
*fliC*718sEBS1a	CGGAGTTGCTGTCCCCGTACGCTGAgtggtgAGCAGCGTATCCAATCC	
*fliC*735aIBS12	AGCCAAAGCAGGTTGACTAGTAActgttactgcgtaGTGCGACGCGAAAGCTAG	
*fliC*735aEBS2s	CGCTAGAAGCCTCGTTAaacagAGCAGGCCAAAGATGCTG	Targeting *fliC*735a
*fliC*735aEBS1a	CGGAGTTGCTGTCCCCGTACGCTGAtgcgtaAGCAGCGTATCCAATCC	
*fliC*744sIBS12	AGCCAAAGCAGGTTGACTAGTAAgtattacgcagtaGTGCGACGCGAAAGCTAG	Targeting *fliC*744s
*fliC*744sEBS2s	CGCTAGAAGCCTCGTTAaatacAGCAGGCCAAAGATGCTG	

*fliC*744sEBS1a	CGGAGTTGCTGTCCCCGTACGCTGAgcagtaAGCAGCGTATCCAATCC	
*fliC*768sIBS12	AGCCAAAGCAGGTTGACTAGTAAtgatggtacagtgGTGCGACGCGAAAGCTAG	
*fliC*768sEBS2s	CGCTAGAAGCCTCGTTAcatcaAGCAGGCCAAAGATGCTG	Targeting *fliC*768s
*fliC*768sEBS1a	CGGAGTTGCTGTCCCCGTACGCTGAacagtgAGCAGCGTATCCAATCC	
*fliC*785sIBS12	AGCCAAAGCAGGTTGACTAGTAAtggcgactggagcGTGCGACGCGAAAGCTAG	
*fliC*785sEBS2s	CGCTAGAAGCCTCGTTAcgccaAGCAGGCCAAAGATGCTG	Targeting *fliC*785s
*fliC*785sEBS1a	CGGAGTTGCTGTCCCCGTACGCTGAtggagcAGCAGCGTATCCAATCC	
*fliC*812sIBS12	AGCCAAAGCAGGTTGACTAGTAActgtaactgatgcGTGCGACGCGAAAGCTAG	
*fliC*812sEBS2s	CGCTAGAAGCCTCGTTAtacagAGCAGGCCAAAGATGCTG	Targeting *fliC*812s
*fliC*812sEBS1a	CGGAGTTGCTGTCCCCGTACGCTGAtgatgcAGCAGCGTATCCAATCC	
*fliC*828sIBS12	AGCCAAAGCAGGTTGACTAGTAAtactactaaagctGTGCGACGCGAAAGCTAG	
*fliC*828sEBS2s	CGCTAGAAGCCTCGTTAtagtaAGCAGGCCAAAGATGCTG	Targeting *fliC*828s
*fliC*828sEBS1a	CGGAGTTGCTGTCCCCGTACGCTGAaaagctAGCAGCGTATCCAATCC	
*fliC*837sIBS12	AGCCAAAGCAGGTTGACTAGTAAagctacaactatcGTGCGACGCGAAAGCTAG	
*fliC*837sEBS2s	CGCTAGAAGCCTCGTTAtagctAGCAGGCCAAAGATGCTG	Targeting *fliC*837s
*fliC*837sEBS1a	CGGAGTTGCTGTCCCCGTACGCTGAactatcAGCAGCGTATCCAATCC	
*fliC*849aIBS12	AGCCAAAGCAGGTTGACTAGTAAtctgaacaggtgtGTGCGACGCGAAAGCTAG	
*fliC*849aEBS2s	CGCTAGAAGCCTCGTTAtcagaAGCAGGCCAAAGATGCTG	Targeting *fliC*849a
*fliC*849aEBS1a	CGGAGTTGCTGTCCCCGTACGCTGAaggtgtAGCAGCGTATCCAATCC	
*fliC*896aIBS12	AGCCAAAGCAGGTTGACTAGTAAgctaacagcaccaGTGCGACGCGAAAGCTAG	
*fliC*896aEBS2s	CGCTAGAAGCCTCGTTAttagcAGCAGGCCAAAGATGCTG	Targeting *fliC*896a
*fliC*896aEBS1a	CGGAGTTGCTGTCCCCGTACGCTGAgcaccaAGCAGCGtATCCAATCC	
*fliC*906sIBS12	AGCCAAAGCAGGTTGACTAGTAAccttggtgctgttGTGCGACGCGAAAGCTAG	
*fliC*906sEBS2s	CGCTAGAAGCCTCGTTAcaaggAGCAGGCCAAAGATGCTG	Targeting *fliC*906s
*fliC*906sEBS1a	CGGAGTTGCTGTCCCCGTACGCTGAgctgttAGCAGCGTATCCAATCC	
*fliC*930sIBS12	AGCCAAAGCAGGTTGACTAGTAAactgcaggattccGTGCGACGCGAAAGCTAG	
*fliC*930sEBS2s	CGCTAGAAGCCTCGTTAgcagtAGCAGGCCAAAGATGCTG	Targeting *fliC*930s
*fliC*930sEBS1a	CGGAGTTGCTGTCCCCGTACGCTGAgattccAGCAGCGTATCCAATCC	
*fliC*931sIBS12	AGCCAAAGCAGGTTGACTAGTAActgcaggattccaGTGCGACGCGAAAGCTAG	
*fliC*931sEBS2s	CGCTAGAAGCCTCGTTAtgcagAGCAGGCCAAAGATGCTG	Targeting *fliC*931s
*fliC*931sEBS1a	CGGAGTTGCTGTCCCCGTACGCTGAattccaAGCAGCGTATCCAATCC	
*fliC*966aIBS12	AGCCAAAGCAGGTTGACTAGTAAgattgccatttgtGTGCGACGCGAAAGCTAG	
*fliC*966aEBS2s	CGCTAGAAGCCTCGTTAcaatcAGCAGGCCAAAGATGCTG	Targeting *fliC*966a
*fliC*966aEBS1a	CGGAGTTGCTGTCCCCGTACGCTGAatttgtAGCAGCGtATCCAATCC	
*fliC*975sIBS12	AGCCAAAGCAGGTTGACTAGTAAagatacaaatggcGTGCGACGCGAAAGCTAG	
*fliC*975sEBS2s	CGCTAGAAGCCTCGTTAtatctAGCAGGCCAAAGATGCTG	Targeting *fliC*975s
*fliC*975sEBS1a	CGGAGTTGCTGTCCCCGTACGCTGAaatggcAGCAGCGTATCCAATCC	
*fliC*1008aIBS12	AGCCAAAGCAGGTTGACTAGTAAcagaaacagcaccGTGCGACGCGAAAGCTAG	
*fliC*1008aEBS2s	CGCTAGAAGCCTCGTTAttctgAGCAGGCCAAAGATGCTG	Targeting *fliC*1008s
*fliC*1008aEBS1a	CGGAGTTGCTGTCCCCGTACGCTGAagcaccAGCAGCGtATCCAATCC	
*fliC*1017aIBS12	AGCCAAAGCAGGTTGACTAGTAAtagttttaacagaGTGCGACGCGAAAGCTAG	
*fliC*1017aEBS2s	CGCTAGAAGCCTCGTTAaactaAGCAGGCCAAAGATGCTG	Targeting *fliC*1017a
*fliC*1017aEBS1a	CGGAGTTGCTGTCCCCGTACGCTGAaacagaAGCAGCGTATCCAATCC	
*fliC*1038sIBS12	AGCCAAAGCAGGTTGACTAGTAAaactattacctatGTGCGACGCGAAAGCTAG	
*fliC*1038sEBS2s	CGCTAGAAGCCTCGTTAtagttAGCAGGCCAAAGATGCTG	Targeting *fliC*1038s
*fliC*1038sEBS1a	CGGAGTTGCTGTCCCCGTACGCTGAacctatAGCAGCGTATCCAATCC	

*fliC*1093sIBS12	AGCCAAAGCAGGTTGACTAGTAActgggcggagatgGTGCGACGCGAAAGCTAG	
*fliC*1093sEBS2s	CGCTAGAAGCCTCGTTAcccagAGCAGGCCAAAGATGCTG	Targeting *fliC*1093s
*fliC*1093sEBS1a	CGGAGTTGCTGTCCCCGTACGCTGAgagatgAGCAGCGTATCCAATCC	
*fliC*1114sIBS12	AGCCAAAGCAGGTTGACTAGTAAacagaagtggtcgGTGCGACGCGAAAGCTAG	
*fliC*1114sEBS2s	CGCTAGAAGCCTCGTTAtctgtAGCAGGCCAAAGATGCTG	Targeting *fliC*1114s
*fliC*1114sEBS1a	CGGAGTTGCTGTCCCCGTACGCTGAtggtcgAGCAGCGTATCCAATCC	
*fliC*1120sIBS12	AGCCAAAGCAGGTTGACTAGTAAgtggtcgatattgGTGCGACGCGAAAGCTAG	
*fliC*1120sEBS2s	CGCTAGAAGCCTCGTTAaccacAGCAGGCCAAAGATGCTG	Targeting *fliC*1120s
*fliC*1120sEBS1a	CGGAGTTGCTGTCCCCGTACGCTGAatattgAGCAGCGTATCCAATCC	
*fliC*22sIBS12	AGCCAAAGCAGGTTGACTAGTAAgtcattaataccaGTGCGACGCGAAAGCTAG	
*fliC*22sEBS2s	CGCTAGAAGCCTCGTTAatgacAGCAGGCCAAAGATGCTG	Targeting *fliC*22s
*fliC*22sEBS1a	CGGAGTTGCTGTCCCCGTACGCTGAataccaAGCAGCGTATCCAATCC	
*fliC*58sIBS12	AGCCAAAGCAGGTTGACTAGTAAaataatatcaacaGTGCGACGCGAAAGCTAG	
*fliC*58sEBS2s	CGCTAGAAGCCTCGTTAttattAGCAGGCCAAAGATGCTG	Targeting *fliC*58s
*fliC*58sEBS1a	CGGAGTTGCTGTCCCCGTACGCTGAtcaacaAGCAGCGTATCCAATCC	
*fliC*99aIBS12	AGCCAAAGCAGGTTGACTAGTAAtacgcaagccagaGTGCGACGCGAAAGCTAG	
*fliC*99aEBS2s	CGCTAGAAGCCTCGTTAgcgtaAGCAGGCCAAAGATGCTG	Targeting *fliC*99s
*fliC*99aEBS1a	CGGAGTTGCTGTCCCCGTACGCTGAgccagaAGCAGCGtATCCAATCC	
*fliC*168sIBS12	AGCCAAAGCAGGTTGACTAGTAAccgtttcacctctGTGCGACGCGAAAGCTAG	
*fliC*168sEBS2s	CGCTAGAAGCCTCGTTAaacggAGCAGGCCAAAGATGCTG	Targeting *fliC*168s
*fliC*168sEBS1a	CGGAGTTGCTGTCCCCGTACGCTGAacctctAGCAGCGTATCCAATCC	
*fliC*159aIBS12	AGCCAAAGCAGGTTGACTAGTAAtgttagaggtgaaGTGCGACGCGAAAGCTAG	
*fliC*159aEBS2s	CGCTAGAAGCCTCGTTAtaacaAGCAGGCCAAAGATGCTG	Targeting *fliC*159a
*fliC*159aEBS1a	CGGAGTTGCTGTCCCCGTACGCTGAggtgaaAGCAGCGtATCCAATCC	
*fliC*183sIBS12	AGCCAAAGCAGGTTGACTAGTAAcattaaaggcctgGTGCGACGCGAAAGCTAG	
*fliC*183sEBS2s	CGCTAGAAGCCTCGTTAtaatgAGCAGGCCAAAGATGCTG	Targeting *fliC*183s
*fliC*183sEBS1a	CGGAGTTGCTGTCCCCGTACGCTGAggcctgAGCAGCGTATCCAATCC	
*fliC*213sIBS12	AGCCAAAGCAGGTTGACTAGTAAcgccaacgacggtGTGCGACGCGAAAGCTAG	
*fliC*213sEBS2s	CGCTAGAAGCCTCGTTAtggcgAGCAGGCCAAAGATGCTG	Targeting *fliC*213s
*fliC*213sEBS1a	CGGAGTTGCTGTCCCCGTACGCTGAgacggtAGCAGCGTATCCAATCC	
*fliC*333aIBS12	AGCCAAAGCAGGTTGACTAGTAAtttcgtcctggatGTGCGACGCGAAAGCTAG	
*fliC*333aEBS2s	CGCTAGAAGCCTCGTTAcgaaaAGCAGGCCAAAGATGCTG	Targeting *fliC*333a
*fliC*333aEBS1a	CGGAGTTGCTGTCCCCGTACGCTGActggatAGCAGCGtATCCAATCC	
*fliC*364sIBS12	AGCCAAAGCAGGTTGACTAGTAAtcccgtctggatgGTGCGACGCGAAAGCTAG	
*fliC*364sEBS2s	CGCTAGAAGCCTCGTTAcgggaAGCAGGCCAAAGATGCTG	Targeting *fliC*364s
*fliC*364sEBS1a	CGGAGTTGCTGTCCCCGTACGCTGAtggatgAGCAGCGTATCCAATCC	
*fliC*433sIBS12	AGCCAAAGCAGGTTGACTAGTAAaatggctccatgaGTGCGACGCGAAAGCTAG	
*fliC*433sEBS2s	CGCTAGAAGCCTCGTTAccattAGCAGGCCAAAGATGCTG	Targeting *fliC*433s
*fliC*433sEBS1a	CGGAGTTGCTGTCCCCGTACGCTGAccatgaAGCAGCGTATCCAATCC	
*fliC*471sIBS12	AGCCAAAGCAGGTTGACTAGTAAccagactatcactGTGCGACGCGAAAGCTAG	
*fliC*471sEBS2s	CGCTAGAAGCCTCGTTAtctggAGCAGGCCAAAGATGCTG	Targeting *fliC*471s
*fliC*471sEBS1a	CGGAGTTGCTGTCCCCGTACGCTGAatcactAGCAGCGTATCCAATCC	
*fliC*503aIBS12	AGCCAAAGCAGGTTGACTAGTAAaccatcaaggccaGTGCGACGCGAAAGCTAG	
*fliC*503aEBS2s	CGCTAGAAGCCTCGTTAatggtAGCAGGCCAAAGATGCTG	Targeting *fliC*503a
*fliC*503aEBS1a	CGGAGTTGCTGTCCCCGTACGCTGAaggccaAGCAGCGtATCCAATCC	
*fliC*546sIBS12	AGCCAAAGCAGGTTGACTAGTAAcgatacagttaccGTGCGACGCGAAAGCTAG	Targeting *fliC*546s

*fliC*546sEBS2s	CGCTAGAAGCCTCGTTAtatcgAGCAGGCCAAAGATGCTG	
*fliC*546sEBS1a	CGGAGTTGCTGTCCCCGTACGCTGAgttaccAGCAGCGTATCCAATCC	
*fliC*555aIBS12	AGCCAAAGCAGGTTGACTAGTAAaagcagttactggGTGCGACGCGAAAGCTAG	
*fliC*555aEBS2s	CGCTAGAAGCCTCGTTAtgcttAGCAGGCCAAAGATGCTG	Targeting *fliC*555a
*fliC*555aEBS1a	CGGAGTTGCTGTCCCCGTACGCTGAtactggAGCAGCGtATCCAATCC	
*fliC*594aIBS12	AGCCAAAGCAGGTTGACTAGTAAttccagtaagtttGTGCGACGCGAAAGCTAG	
*fliC*594aEBS2s	CGCTAGAAGCCTCGTTAtggaaAGCAGGCCAAAGATGCTG	Targeting *fliC*594a
*fliC*594aEBS1a	CGGAGTTGCTGTCCCCGTACGCTGAaagtttAGCAGCGtATCCAATCC	
*fliC*976sIBS12	AGCCAAAGCAGGTTGACTAGTAAgatacaaatggcaGTGCGACGCGAAAGCTAG	
*fliC*976sEBS2s	CGCTAGAAGCCTCGTTAgtatcAGCAGGCCAAAGATGCTG	Targeting *fliC*976s
*fliC*976sEBS1a	CGGAGTTGCTGTCCCCGTACGCTGAatggcaAGCAGCGTATCCAATCC	
*fliC*1048aIBS12	AGCCAAAGCAGGTTGACTAGTAActggcggcaccggGTGCGACGCGAAAGCTAG	
*fliC*1048aEBS2s	CGCTAGAAGCCTCGTTAgccagAGCAGGCCAAAGATGCTG	Targeting *fliC*1048a
*fliC*1048aEBS1a	CGGAGTTGCTGTCCCCGTACGCTGAcaccggAGCAGCGtATCCAATCC	
*fliC*1132aIBS12	AGCCAAAGCAGGTTGACTAGTAAtcggcagaatcgtGTGCGACGCGAAAGCTAG	
*fliC*1132aEBS2s	CGCTAGAAGCCTCGTTAgccgaAGCAGGCCAAAGATGCTG	Targeting *fliC1132a*
*fliC*1132aEBS1a	CGGAGTTGCTGTCCCCGTACGCTGAaatcgtAGCAGCGtATCCAATCC	
*fliC*1302aIBS12	AGCCAAAGCAGGTTGACTAGTAAccgcggaatccagGTGCGACGCGAAAGCTAG	
*fliC*1302aEBS2s	CGCTAGAAGCCTCGTTAcgcggAGCAGGCCAAAGATGCTG	Targeting *fliC*1302a
*fliC*1302aEBS1a	CGGAGTTGCTGTCCCCGTACGCTGAatccagAGCAGCGtATCCAATCC	
*fliC*1335sIBS12	AGCCAAAGCAGGTTGACTAGTAAcctgaacaacaccGTGCGACGCGAAAGCTAG	
*fliC*1335sEBS2s	CGCTAGAAGCCTCGTTAtcaggAGCAGGCCAAAGATGCTG	Targeting *fliC*1335s
*fliC*1335sEBS1a	CGGAGTTGCTGTCCCCGTACGCTGAaacaccAGCAGCGTATCCAATCC	
*fliC*1341sIBS12	AGCCAAAGCAGGTTGACTAGTAAcaacaccactaccGTGCGACGCGAAAGCTAG	
*fliC*1341sEBS2s	CGCTAGAAGCCTCGTTAtgttgAGCAGGCCAAAGATGCTG	Targeting *fliC*1341s
*fliC*1341sEBS1a	CGGAGTTGCTGTCCCCGTACGCTGAactaccAGCAGCGTATCCAATCC	
*fliC*1423sIBS12	AGCCAAAGCAGGTTGACTAGTAAgcgcagatcatccGTGCGACGCGAAAGCTAG	
*fliC*1423sEBS2s	CGCTAGAAGCCTCGTTAtgcgcAGCAGGCCAAAGATGCTG	Targeting *fliC*1423s
*fliC*1423sEBS1a	CGGAGTTGCTGTCCCCGTACGCTGAtcatccAGCAGCGTATCCAATCC	
*fliC*1449sIBS12	AGCCAAAGCAGGTTGACTAGTAActccgtgttggcaGTGCGACGCGAAAGCTAG	
*fliC*1449sEBS2s	CGCTAGAAGCCTCGTTAcggagAGCAGGCCAAAGATGCTG	Targeting *fliC*1449s
*fliC*1449sEBS1a	CGGAGTTGCTGTCCCCGTACGCTGAttggcaAGCAGCGTATCCAATCC	
*fliC*1463sIBS12	AGCCAAAGCAGGTTGACTAGTAAaagctaaccaggtGTGCGACGCGAAAGCTAG	
*fliC*1463sEBS2s	CGCTAGAAGCCTCGTTAagcttAGCAGGCCAAAGATGCTG	Targeting *fliC*1463s
*fliC*1463sEBS1a	CGGAGTTGCTGTCCCCGTACGCTGAccaggtAGCAGCGTATCCAATCC	
*fliC*1480aIBS12	AGCCAAAGCAGGTTGACTAGTAAccctgcagcagagGTGCGACGCGAAAGCTAG	
*fliC*1480aEBS2s	CGCTAGAAGCCTCGTTAcagggAGCAGGCCAAAGATGCTG	Targeting *fliC*1480a
*fliC*1480aEBS1a	CGGAGTTGCTGTCCCCGTACGCTGAgcagagAGCAGCGtATCCAATCC	
*lacZ*18aIBS12	AGCCAAAGCAGGTTGACTAGTAAcgacggccagtgaGTGCGACGCGAAAGCTAG	
*lacZ*18aEBS2s	CGCTAGAAGCCTCGTTAcgtcgAGCAGGCCAAAGATGCTG	Targeting *lacZ*18a
*lacZ*18aEBS1a	CGGAGTTGCTGTCCCCGTACGCTGAcagtgaAGCAGCGtATCCAATCC	
*lacZ*19aIBS12	AGCCAAAGCAGGTTGACTAGTAAacgacggccagtgGTGCGACGCGAAAGCTAG	
*lacZ*19aEBS2s	CGCTAGAAGCCTCGTTAgtcgtAGCAGGCCAAAGATGCTG	Targeting *lacZ*19a
*lacZ*19aEBS1a	CGGAGTTGCTGTCCCCGTACGCTGAccagtgAGCAGCGtATCCAATCC	
*lacZ*60aIBS12	AGCCAAAGCAGGTTGACTAGTAAgttgggtaacgccGTGCGACGCGAAAGCTAG	Targeting *lacZ*60a
*lacZ*60aEBS2s	CGCTAGAAGCCTCGTTAccaacAGCAGGCCAAAGATGCTG	

*lacZ*60aEBS1a	CGGAGTTGCTGTCCCCGTACGCTGAaacgccAGCAGCGtATCCAATCC	
*lacZ*136sIBS12	AGCCAAAGCAGGTTGACTAGTAAgaggcccgcaccgGTGCGACGCGAAAGCTAG	
*lacZ*136sEBS2s	CGCTAGAAGCCTCGTTAgcctcAGCAGGCCAAAGATGCTG	Targeting *lacZ*136s
*lacZ*136sEBS1a	CGGAGTTGCTGTCCCCGTACGCTGAgcaccgAGCAGCGTATCCAATCC	
*lacZ*271aIBS12	AGCCAAAGCAGGTTGACTAGTAAccgtgcatctgccGTGCGACGCGAAAGCTAG	
*lacZ*271aEBS2s	CGCTAGAAGCCTCGTTAcacggAGCAGGCCAAAGATGCTG	Targeting *lacZ*271a
*lacZ*271aEBS1a	CGGAGTTGCTGTCCCCGTACGCTGAtctgccAGCAGCGtATCCAATCC	
*lacZ*410sIBS12	AGCCAAAGCAGGTTGACTAGTAAgctggctacaggaGTGCGACGCGAAAGCTAG	
*lacZ*410sEBS2s	CGCTAGAAGCCTCGTTAccagcAGCAGGCCAAAGATGCTG	Targeting *lacZ*410s
*lacZ*410sEBS1a	CGGAGTTGCTGTCCCCGTACGCTGAacaggaAGCAGCGTATCCAATCC	
*lacZ*516aIBS12	AGCCAAAGCAGGTTGACTAGTAAatgcgctcaggtcGTGCGACGCGAAAGCTAG	
*lacZ*516aEBS2s	CGCTAGAAGCCTCGTTAcgcatAGCAGGCCAAAGATGCTG	Targeting *lacZ*516a
*lacZ*516aEBS1a	CGGAGTTGCTGTCCCCGTACGCTGAcaggtcAGCAGCGtATCCAATCC	
*lacZ*574aIBS12	AGCCAAAGCAGGTTGACTAGTAActgccgtcactccGTGCGACGCGAAAGCTAG	
*lacZ*574aEBS2s	CGCTAGAAGCCTCGTTAggcagAGCAGGCCAAAGATGCTG	Targeting *lacZ*574a
*lacZ*574aEBS1a	CGGAGTTGCTGTCCCCGTACGCTGAcactccAGCAGCGtATCCAATCC	
*lacZ*679sIBS12	AGCCAAAGCAGGTTGACTAGTAAatcagcgatttccGTGCGACGCGAAAGCTAG	
*lacZ*679sEBS2s	CGCTAGAAGCCTCGTTActgatAGCAGGCCAAAGATGCTG	Targeting *lacZ*679s
*lacZ*679sEBS1a	CGGAGTTGCTGTCCCCGTACGCTGAatttccAGCAGCGTATCCAATCC	
*lacZ*681aIBS12	AGCCAAAGCAGGTTGACTAGTAAagcgagtggcaacGTGCGACGCGAAAGCTAG	
*lacZ*681aEBS2s	CGCTAGAAGCCTCGTTAtcgctAGCAGGCCAAAGATGCTG	Targeting *lacZ*681a
*lacZ*681aEBS1a	CGGAGTTGCTGTCCCCGTACGCTGAggcaacAGCAGCGtATCCAATCC	
*lacZ*822aIBS12	AGCCAAAGCAGGTTGACTAGTAAtttcaccgccgaaGTGCGACGCGAAAGCTAG	
*lacZ*822aEBS2s	CGCTAGAAGCCTCGTTAtgaaaAGCAGGCCAAAGATGCTG	Targeting *lacZ*822a
*lacZ*822aEBS1a	CGGAGTTGCTGTCCCCGTACGCTGAgccgaaAGCAGCGtATCCAATCC	
*lacZ*897sIBS12	AGCCAAAGCAGGTTGACTAGTAAcgtcgaaaacccgGTGCGACGCGAAAGCTAG	
*lacZ*897sEBS2s	CGCTAGAAGCCTCGTTAcgacgAGCAGGCCAAAGATGCTG	Targeting *lacZ*897s
*lacZ*897sEBS1a	CGGAGTTGCTGTCCCCGTACGCTGAaacccgAGCAGCGTATCCAATCC	
*lacZ*913sIBS12	AGCCAAAGCAGGTTGACTAGTAActgtggagcgccgGTGCGACGCGAAAGCTAG	
*lacZ*913sEBS2s	CGCTAGAAGCCTCGTTAcacagAGCAGGCCAAAGATGCTG	Targeting *lacZ*913s
*lacZ*913sEBS1a	CGGAGTTGCTGTCCCCGTACGCTGAgcgccgAGCAGCGTATCCAATCC	
*lacZ*988sIBS12	AGCCAAAGCAGGTTGACTAGTAAgcagaagcctgcgGTGCGACGCGAAAGCTAG	
*lacZ*988sEBS2s	CGCTAGAAGCCTCGTTAtctgcAGCAGGCCAAAGATGCTG	Targeting *lacZ*988s
*lacZ*988sEBS1a	CGGAGTTGCTGTCCCCGTACGCTGAcctgcgAGCAGCGTATCCAATCC	
*lacZ*1139aIBS12	AGCCAAAGCAGGTTGACTAGTAAgttgttctgcttcGTGCGACGCGAAAGCTAG	
*lacZ*1139aEBS2s	CGCTAGAAGCCTCGTTAacaacAGCAGGCCAAAGATGCTG	Targeting *lacZ*1139a
*lacZ*1139aEBS1a	CGGAGTTGCTGTCCCCGTACGCTGAtgcttcAGCAGCGtATCCAATCC	
*lacZ*1362sIBS12	AGCCAAAGCAGGTTGACTAGTAAtcacccgagtgtgGTGCGACGCGAAAGCTAG	
*lacZ*1362sEBS2s	CGCTAGAAGCCTCGTTAggtgaAGCAGGCCAAAGATGCTG	Targeting *lacZ*1362s
*lacZ*1362sEBS1a	CGGAGTTGCTGTCCCCGTACGCTGAagtgtgAGCAGCGTATCCAATCC	
*lacZ*1566sIBS12	AGCCAAAGCAGGTTGACTAGTAAatggtccatcaaaGTGCGACGCGAAAGCTAG	
*lacZ*1566sEBS2s	CGCTAGAAGCCTCGTTAaccatAGCAGGCCAAAGATGCTG	Targeting *lacZ*1566a
*lacZ*1566sEBS1a	CGGAGTTGCTGTCCCCGTACGCTGAatcaaaAGCAGCGTATCCAATCC	
*lacZ*1567sIBS12	AGCCAAAGCAGGTTGACTAGTAAtggtccatcaaaaGTGCGACGCGAAAGCTAG	
*lacZ*1567sEBS2s	CGCTAGAAGCCTCGTTAgaccaAGCAGGCCAAAGATGCTG	Targeting *lacZ*1567s
*lacZ*1567sEBS1a	CGGAGTTGCTGTCCCCGTACGCTGAtcaaaaAGCAGCGTATCCAATCC	

*lacZ*1580sIBS12	AGCCAAAGCAGGTTGACTAGTAAaatggctttcgctGTGCGACGCGAAAGCTAG	
*lacZ*1580sEBS2s	CGCTAGAAGCCTCGTTAccattAGCAGGCCAAAGATGCTG	Targeting *lacZ*1580s
*lacZ*1580sEBS1a	CGGAGTTGCTGTCCCCGTACGCTGAttcgctAGCAGCGTATCCAATCC	
*lacZ*1873sIBS12	AGCCAAAGCAGGTTGACTAGTAAgcaaaacaccagcGTGCGACGCGAAAGCTAG	
*lacZ*1873sEBS2s	CGCTAGAAGCCTCGTTAtttgcAGCAGGCCAAAGATGCTG	Targeting *lacZ*1873s
*lacZ*1873sEBS1a	CGGAGTTGCTGTCCCCGTACGCTGAaccagcAGCAGCGTATCCAATCC	
*lacZ*1939sIBS12	AGCCAAAGCAGGTTGACTAGTAAtacctgttccgtcGTGCGACGCGAAAGCTAG	
*lacZ*1939sEBS2s	CGCTAGAAGCCTCGTTAaggtaAGCAGGCCAAAGATGCTG	Targeting *lacZ*1939s
*lacZ*1939sEBS1a	CGGAGTTGCTGTCCCCGTACGCTGAtccgtcAGCAGCGTATCCAATCC	
*lacZ*2586aIBS12	AGCCAAAGCAGGTTGACTAGTAAtcgccatttgaccGTGCGACGCGAAAGCTAG	
*lacZ*2586aEBS2s	CGCTAGAAGCCTCGTTAggcgaAGCAGGCCAAAGATGCTG	Targeting *lacZ*2586a
*lacZ*2586aEBS1a	CGGAGTTGCTGTCCCCGTACGCTGAttgaccAGCAGCGtATCCAATCC	
*lacZ*2725sIBS12	AGCCAAAGCAGGTTGACTAGTAAgaaaactatcccgGTGCGACGCGAAAGCTAG	
*lacZ*2725sEBS2s	CGCTAGAAGCCTCGTTAttttcAGCAGGCCAAAGATGCTG	Targeting *lacZ*2725s
*lacZ*2725sEBS1a	CGGAGTTGCTGTCCCCGTACGCTGAatcccgAGCAGCGTATCCAATCC	
DP-*fliC*-F	AGTCTCAGTTAATCAGGTTACAACG	Detecting *fliC* gene
DP-*fliC*-R	CGTAATCAACGACTTGCAATATAGG	
*lacZ*52sIBS12	AGCCAAAGCAGGTTGACTAGTAAcgtcgtgactgggGTGCGACGCGAAAGCTAG	
*lacZ*52sEBS2s	CGCTAGAAGCCTCGTTAcgacgAGCAGGCCAAAGATGCTG	Targeting *lacZ*52s
*lacZ*52sEBS1a	CGGAGTTGCTGTCCCCGTACGCTGAactgggAGCAGCGTATCCAATCC	
*LacZ*168aIBS12	AGCCAAAGCAGGTTGACTAGTAAagcgccattcgccGTGCGACGCGAAAGCTAG	
*lacZ*168aEBS2s	CGCTAGAAGCCTCGTTAgcgctAGCAGGCCAAAGATGCTG	Targeting *lacZ*168a
*lacZ*168aEBS1a	CGGAGTTGCTGTCCCCGTACGCTGAttcgccAGCAGCGtATCCAATCC	
*lacZ*321sIBS12	AGCCAAAGCAGGTTGACTAGTAAcgtgacctatcccGTGCGACGCGAAAGCTAG	
*lacZ*321sEBS2s	CGCTAGAAGCCTCGTTAtcacgAGCAGGCCAAAGATGCTG	Targeting *lacZ*321s
*lacZ*321sEBS1a	CGGAGTTGCTGTCCCCGTACGCTGAtatcccAGCAGCGTATCCAATCC	
*lacZ*369aIBS12	AGCCAAAGCAGGTTGACTAGTAAatgtgagcgagtaGTGCGACGCGAAAGCTAG	
*lacZ*369aEBS2s	CGCTAGAAGCCTCGTTAcacatAGCAGGCCAAAGATGCTG	Targeting *lacZ*369a
*lacZ*369aEBS1a	CGGAGTTGCTGTCCCCGTACGCTGAcgagtaAGCAGCGtATCCAATCC	
*lacZ*409sIBS12	AGCCAAAGCAGGTTGACTAGTAAagctggctacaggGTGCGACGCGAAAGCTAG	
*lacZ*409sEBS2s	CGCTAGAAGCCTCGTTAcagctAGCAGGCCAAAGATGCTG	Targeting *lacZ*409s
*lacZ*409sEBS1a	CGGAGTTGCTGTCCCCGTACGCTGAtacaggAGCAGCGTATCCAATCC	
*lacZ*426aIBS12	AGCCAAAGCAGGTTGACTAGTAAcgccatcaaaaatGTGCGACGCGAAAGCTAG	
*lacZ*426aEBS2s	CGCTAGAAGCCTCGTTAtggcgAGCAGGCCAAAGATGCTG	Targeting *lacZ*426a
*lacZ*426aEBS1a	CGGAGTTGCTGTCCCCGTACGCTGAaaaaatAGCAGCGtATCCAATCC	
*lacZ*501aIBS12	AGCCAAAGCAGGTTGACTAGTAAattcagacggcaaGTGCGACGCGAAAGCTAG	
*lacZ*501aEBS2s	CGCTAGAAGCCTCGTTAtgaatAGCAGGCCAAAGATGCTG	Targeting *lacZ*501a
*lacZ*501aEBS1a	CGGAGTTGCTGTCCCCGTACGCTGAcggcaaAGCAGCGtATCCAATCC	
*lacZ*515aIBS12	AGCCAAAGCAGGTTGACTAGTAAtgcgctcaggtcaGTGCGACGCGAAAGCTAG	
*lacZ*515aEBS2s	CGCTAGAAGCCTCGTTAgcgcaAGCAGGCCAAAGATGCTG	Targeting *lacZ*515a
*lacZ*515aEBS1a	CGGAGTTGCTGTCCCCGTACGCTGAaggtcaAGCAGCGtATCCAATCC	
*lacZ*561sIBS12	AGCCAAAGCAGGTTGACTAGTAAccgcctcgcggtgGTGCGACGCGAAAGCTAG	
*lacZ*561sEBS2s	CGCTAGAAGCCTCGTTAggcggAGCAGGCCAAAGATGCTG	Targeting *lacZ*561s
*lacZ*561sEBS1a	CGGAGTTGCTGTCCCCGTACGCTGAgcggtgAGCAGCGTATCCAATCC	
*lacZ*629aIBS12	AGCCAAAGCAGGTTGACTAGTAAcgagacgtcacggGTGCGACGCGAAAGCTAG	Targeting *lacZ*629a
*lacZ*629aEBS2s	CGCTAGAAGCCTCGTTAtctcgAGCAGGCCAAAGATGCTG	

*lacZ*629aEBS1a	CGGAGTTGCTGTCCCCGTACGCTGAtcacggAGCAGCGtATCCAATCC	
*lacZ*666sIBS12	AGCCAAAGCAGGTTGACTAGTAAaccgactacacaaGTGCGACGCGAAAGCTAG	
*lacZ*666sEBS2s	CGCTAGAAGCCTCGTTAtcggtAGCAGGCCAAAGATGCTG	Targeting *lacZ*666s
*lacZ*666sEBS1a	CGGAGTTGCTGTCCCCGTACGCTGAacacaaAGCAGCGTATCCAATCC	
*lacZ*906sIBS12	AGCCAAAGCAGGTTGACTAGTAAcccgaaactgtggGTGCGACGCGAAAGCTAG	
*lacZ*906sEBS2s	CGCTAGAAGCCTCGTTAtcgggAGCAGGCCAAAGATGCTG	Targeting *lacZ*906s
*lacZ*906sEBS1a	CGGAGTTGCTGTCCCCGTACGCTGActgtggAGCAGCGTATCCAATCC	
*lacZ*928sIBS12	AGCCAAAGCAGGTTGACTAGTAAatcccgaatctctGTGCGACGCGAAAGCTAG	
*lacZ*928sEBS2s	CGCTAGAAGCCTCGTTAgggatAGCAGGCCAAAGATGCTG	Targeting *lacZ*928s
*lacZ*928sEBS1a	CGGAGTTGCTGTCCCCGTACGCTGAatctctAGCAGCGTATCCAATCC	
*lacZ*958sIBS12	AGCCAAAGCAGGTTGACTAGTAActgcacaccgccgGTGCGACGCGAAAGCTAG	
*lacZ*958sEBS2s	CGCTAGAAGCCTCGTTAtgcagAGCAGGCCAAAGATGCTG	Targeting *lacZ*958s
*lacZ*958sEBS1a	CGGAGTTGCTGTCCCCGTACGCTGAccgccgAGCAGCGTATCCAATCC	
*lacZ*1034aIBS12	AGCCAAAGCAGGTTGACTAGTAAcggcttgccgttcGTGCGACGCGAAAGCTAG	
*lacZ*1034aEBS2s	CGCTAGAAGCCTCGTTAagccgAGCAGGCCAAAGATGCTG	Targeting *lacZ*1034a
*lacZ*1034aEBS1a	CGGAGTTGCTGTCCCCGTACGCTGAccgttcAGCAGCGTATCCAATCC	
*lacZ*1155sIBS12	AGCCAAAGCAGGTTGACTAGTAAgcagaacaactttGTGCGACGCGAAAGCTAG	
*lacZ*1155sEBS2s	CGCTAGAAGCCTCGTTAtctgcAGCAGGCCAAAGATGCTG	Targeting *lacZ*1155s
*lacZ*1155sEBS1a	CGGAGTTGCTGTCCCCGTACGCTGAaactttAGCAGCGTATCCAATCC	
*lacZ*1477sIBS12	AGCCAAAGCAGGTTGACTAGTAAggcggcggagccgGTGCGACGCGAAAGCTAG	
*lacZ*1477sEBS2s	CGCTAGAAGCCTCGTTAccgccAGCAGGCCAAAGATGCTG	Targeting *lacZ*1477s
*lacZ*1477sEBS1a	CGGAGTTGCTGTCCCCGTACGCTGAgagccgAGCAGCGTATCCAATCC	
*lacZ*1764aIBS12	AGCCAAAGCAGGTTGACTAGTAAaatcaccgccgtaGTGCGACGCGAAAGCTAG	
*lacZ*1764aEBS2s	CGCTAGAAGCCTCGTTAtgattAGCAGGCCAAAGATGCTG	Targeting *lacZ*1764a
*lacZ*1764aEBS1a	CGGAGTTGCTGTCCCCGTACGCTGAgccgtaAGCAGCGTATCCAATCC	
*lacZ*1811aIBS12	AGCCAAAGCAGGTTGACTAGTAAgaccagaccgttcGTGCGACGCGAAAGCTAG	
*lacZ*1811aEBS2s	CGCTAGAAGCCTCGTTAtggtcAGCAGGCCAAAGATGCTG	Targeting *lacZ*1811a
*lacZ*1811aEBS1a	CGGAGTTGCTGTCCCCGTACGCTGAccgttcAGCAGCGTATCCAATCC	
*lacZ*1879aIBS12	AGCCAAAGCAGGTTGACTAGTAAcggaactggaaaaGTGCGACGCGAAAGCTAG	
*lacZ*1879aEBS2s	CGCTAGAAGCCTCGTTAttccgAGCAGGCCAAAGATGCTG	Targeting *lacZ*1879a
*lacZ*1879aEBS1a	CGGAGTTGCTGTCCCCGTACGCTGAggaaaaAGCAGCGTATCCAATCC	
*lacZ*1880aIBS12	AGCCAAAGCAGGTTGACTAGTAAacggaactggaaaGTGCGACGCGAAAGCTAG	
*lacZ*1880aEBS2s	CGCTAGAAGCCTCGTTAtccgtAGCAGGCCAAAGATGCTG	Targeting *lacZ*1880a
*lacZ*1880aEBS1a	CGGAGTTGCTGTCCCCGTACGCTGAtggaaaAGCAGCGTATCCAATCC	
*lacZ*1927sIBS12	AGCCAAAGCAGGTTGACTAGTAAgtgaccagcgaatGTGCGACGCGAAAGCTAG	
*lacZ*1927sEBS2s	CGCTAGAAGCCTCGTTAgtcacAGCAGGCCAAAGATGCTG	Targeting *lacZ*1927s
*lacZ*1927sEBS1a	CGGAGTTGCTGTCCCCGTACGCTGAgcgaatAGCAGCGTATCCAATCC	
*lacZ*2068sIBS12	AGCCAAAGCAGGTTGACTAGTAActaccgcagccggGTGCGACGCGAAAGCTAG	
*lacZ*2068sEBS2s	CGCTAGAAGCCTCGTTAggtagAGCAGGCCAAAGATGCTG	Targeting *lacZ*2068s
*lacZ*2068sEBS1a	CGGAGTTGCTGTCCCCGTACGCTGAagccggAGCAGCGTATCCAATCC	
*lacZ*2262aIBS12	AGCCAAAGCAGGTTGACTAGTAAattgccaacgcttGTGCGACGCGAAAGCTAG	
*lacZ*2262aEBS2s	CGCTAGAAGCCTCGTTAgcaatAGCAGGCCAAAGATGCTG	Targeting *lacZ*2262a
*lacZ*2262aEBS1a	CGGAGTTGCTGTCCCCGTACGCTGAacgcttAGCAGCGTATCCAATCC	
*lacZ*2277sIBS12	AGCCAAAGCAGGTTGACTAGTAAgcgttggcaatttGTGCGACGCGAAAGCTAG	
*lacZ*2277sEBS2s	CGCTAGAAGCCTCGTTAaacgcAGCAGGCCAAAGATGCTG	Targeting *lacZ*2277s
*lacZ*2277sEBS1a	CGGAGTTGCTGTCCCCGTACGCTGAcaatttAGCAGCGTATCCAATCC	

*lacZ*2298aIBS12	AGCCAAAGCAGGTTGACTAGTAAtccacatctgtgaGTGCGACGCGAAAGCTAG	
*lacZ*2298aEBS2s	CGCTAGAAGCCTCGTTAgtggaAGCAGGCCAAAGATGCTG	Targeting *lacZ*2298a
*lacZ*2298aEBS1a	CGGAGTTGCTGTCCCCGTACGCTGActgtgaAGCAGCGTATCCAATCC	
*lacZ*2334sIBS12	AGCCAAAGCAGGTTGACTAGTAAaaaacaactgctgGTGCGACGCGAAAGCTAG	
*lacZ*2334sEBS2s	CGCTAGAAGCCTCGTTAgttttAGCAGGCCAAAGATGCTG	Targeting *lacZ*2334s
*lacZ*2334sEBS1a	CGGAGTTGCTGTCCCCGTACGCTGActgctgAGCAGCGTATCCAATCC	
*lacZ*2388sIBS12	AGCCAAAGCAGGTTGACTAGTAAcgacattggcgtaGTGCGACGCGAAAGCTAG	
*lacZ*2388sEBS2s	CGCTAGAAGCCTCGTTAtgtcgAGCAGGCCAAAGATGCTG	Targeting *lacZ*2388s
*lacZ*2388sEBS1a	CGGAGTTGCTGTCCCCGTACGCTGAggcgtaAGCAGCGTATCCAATCC	
*lacZ*2391aIBS12	AGCCAAAGCAGGTTGACTAGTAAtgcgggtcgcttcGTGCGACGCGAAAGCTAG	
*lacZ*2391aEBS2s	CGCTAGAAGCCTCGTTAccgcaAGCAGGCCAAAGATGCTG	Targeting *lacZ*2391a
*lacZ*2391aEBS1a	CGGAGTTGCTGTCCCCGTACGCTGAcgcttcAGCAGCGTATCCAATCC	
*lacZ*2407sIBS12	AGCCAAAGCAGGTTGACTAGTAAgcgacccgcattgGTGCGACGCGAAAGCTAG	
*lacZ*2407sEBS2s	CGCTAGAAGCCTCGTTAgtcgcAGCAGGCCAAAGATGCTG	Targeting *lacZ*2407s
*lacZ*2407sEBS1a	CGGAGTTGCTGTCCCCGTACGCTGAgcattgAGCAGCGTATCCAATCC	
*lacZ*2473sIBS12	AGCCAAAGCAGGTTGACTAGTAAgcagcgttgttgcGTGCGACGCGAAAGCTAG	
*lacZ*2473sEBS2s	CGCTAGAAGCCTCGTTAgctgcAGCAGGCCAAAGATGCTG	Targeting *lacZ*2473s
*lacZ*2473sEBS1a	CGGAGTTGCTGTCCCCGTACGCTGAtgttgcAGCAGCGTATCCAATCC	
*lacZ*2496aIBS12	AGCCAAAGCAGGTTGACTAGTAAtcagcaccgcatcGTGCGACGCGAAAGCTAG	
*lacZ*2496aEBS2s	CGCTAGAAGCCTCGTTAgctgaAGCAGGCCAAAGATGCTG	Targeting *lacZ*2496a
*lacZ*2496aEBS1a	CGGAGTTGCTGTCCCCGTACGCTGAcgcatcAGCAGCGTATCCAATCC	
*lacZ*2556sIBS12	AGCCAAAGCAGGTTGACTAGTAAaaccttatttatcGTGCGACGCGAAAGCTAG	
*lacZ*2556sEBS2s	CGCTAGAAGCCTCGTTAaggttAGCAGGCCAAAGATGCTG	Targeting *lacZ*2556s
*lacZ*2556sEBS1a	CGGAGTTGCTGTCCCCGTACGCTGAtttatcAGCAGCGTATCCAATCC	
*lacZ*2578sIBS12	AGCCAAAGCAGGTTGACTAGTAAacctaccggattgGTGCGACGCGAAAGCTAG	
*lacZ*2578sEBS2s	CGCTAGAAGCCTCGTTAtaggtAGCAGGCCAAAGATGCTG	Targeting *lacZ*2578s
*lacZ*2578sEBS1a	CGGAGTTGCTGTCCCCGTACGCTGAggattgAGCAGCGTATCCAATCC	
*lacZ*2687aIBS12	AGCCAAAGCAGGTTGACTAGTAAtccgagccagtttGTGCGACGCGAAAGCTAG	
*lacZ*2687aEBS2s	CGCTAGAAGCCTCGTTAtcggaAGCAGGCCAAAGATGCTG	Targeting *lacZ*2687a
*lacZ*2687aEBS1a	CGGAGTTGCTGTCCCCGTACGCTGAcagtttAGCAGCGTATCCAATCC	
*lacZ*2702sIBS12	AGCCAAAGCAGGTTGACTAGTAAactggctcggattGTGCGACGCGAAAGCTAG	
*lacZ*2702sEBS2s	CGCTAGAAGCCTCGTTAccagtAGCAGGCCAAAGATGCTG	Targeting *lacZ*2702s
*lacZ*2702sEBS1a	CGGAGTTGCTGTCCCCGTACGCTGAcggattAGCAGCGTATCCAATCC	
*lacZ*2732aIBS12	AGCCAAAGCAGGTTGACTAGTAAacaggcggcagtaGTGCGACGCGAAAGCTAG	
*lacZ*2732aEBS2s	CGCTAGAAGCCTCGTTAcctgtAGCAGGCCAAAGATGCTG	Targeting *lacZ*2732a
*lacZ*2732aEBS1a	CGGAGTTGCTGTCCCCGTACGCTGAgcagtaAGCAGCGTATCCAATCC	
*lacZ*2733aIBS12	AGCCAAAGCAGGTTGACTAGTAAaacaggcggcagtGTGCGACGCGAAAGCTAG	
*lacZ*2733aEBS2s	CGCTAGAAGCCTCGTTActgttAGCAGGCCAAAGATGCTG	Targeting *lacZ*2733a
*lacZ*2733aEBS1a	CGGAGTTGCTGTCCCCGTACGCTGAggcagtAGCAGCGTATCCAATCC	
*lacZ*2889sIBS12	AGCCAAAGCAGGTTGACTAGTAAcatcagccgctacGTGCGACGCGAAAGCTAG	
*lacZ*2889sEBS2s	CGCTAGAAGCCTCGTTAtgatgAGCAGGCCAAAGATGCTG	Targeting *lacZ*2889s
*lacZ*2889sEBS1a	CGGAGTTGCTGTCCCCGTACGCTGAcgctacAGCAGCGTATCCAATCC	
*lacZ*2908sIBS12	AGCCAAAGCAGGTTGACTAGTAAcagcaactgatggGTGCGACGCGAAAGCTAG	
*lacZ*2908sEBS2s	CGCTAGAAGCCTCGTTAtgctgAGCAGGCCAAAGATGCTG	Targeting *lacZ*2908s
*lacZ*2908sEBS1a	CGGAGTTGCTGTCCCCGTACGCTGAtgatggAGCAGCGTATCCAATCC	
*lacZ*2923sIBS12	AGCCAAAGCAGGTTGACTAGTAAaccagccatcgccGTGCGACGCGAAAGCTAG	Targeting *lacZ*2923s

*lacZ*2923sEBS2s	CGCTAGAAGCCTCGTTActggtAGCAGGCCAAAGATGCTG	
*lacZ*2923sEBS1a	CGGAGTTGCTGTCCCCGTACGCTGAatcgccAGCAGCGTATCCAATCC	
*lacZ*2954aIBS12	AGCCAAAGCAGGTTGACTAGTAAaccgtcgatattcGTGCGACGCGAAAGCTAG	
*lacZ*2954aEBS2s	CGCTAGAAGCCTCGTTAacggtAGCAGGCCAAAGATGCTG	Targeting *lacZ*2954a
*lacZ*2954aEBS1a	CGGAGTTGCTGTCCCCGTACGCTGAatattcAGCAGCGTATCCAATCC	
*lacZ*2956sIBS12	AGCCAAAGCAGGTTGACTAGTAAggcacatggctgaGTGCGACGCGAAAGCTAG	
*lacZ*2956sEBS2s	CGCTAGAAGCCTCGTTAgtgccAGCAGGCCAAAGATGCTG	Targeting *lacZ*2956s
*lacZ*2956sEBS1a	CGGAGTTGCTGTCCCCGTACGCTGAggctgaAGCAGCGTATCCAATCC	
*lacZ*2967aIBS12	AGCCAAAGCAGGTTGACTAGTAAtccccatatggaaGTGCGACGCGAAAGCTAG	
*lacZ*2967aEBS2s	CGCTAGAAGCCTCGTTAggggaAGCAGGCCAAAGATGCTG	Targeting *lacZ*2967a
*lacZ*2967aEBS1a	CGGAGTTGCTGTCCCCGTACGCTGAatggaaAGCAGCGTATCCAATCC	
DP-*lacZ*-F	TTACGCGAAATACGGGCAG	Detecting *lacZ* gene
DP-*lacZ*-R	CTTCCGGCTCGTATGTTGT	
*dctA*2aIBS12	AGCCAAAGCAGGTTGACTAGTAAcagagaggttttcGTGCGACGCGAAAGCTAG	
*dctA*2aEBS2s	CGCTAGAAGCCTCGTTActctgAGCAGGCCAAAGATGCTG	Targeting *dctA*2a
*dctA*2aEBS1a	CGGAGTTGCTGTCCCCGTACGCTGAgttttcAGCAGCGTATCCAATCC	
*dctA*18aIBS12	AGCCAAAGCAGGTTGACTAGTAAagtaaaggcttttGTGCGACGCGAAAGCTAG	
*dctA*18aEBS2s	CGCTAGAAGCCTCGTTAttactAGCAGGCCAAAGATGCTG	Targeting *dctA*18a
*dctA*18aEBS1a	CGGAGTTGCTGTCCCCGTACGCTGAgcttttAGCAGCGTATCCAATCC	
*dctA*18sIBS12	AGCCAAAGCAGGTTGACTAGTAAaacctctctgtttGTGCGACGCGAAAGCTAG	
*dctA*18sEBS2s	CGCTAGAAGCCTCGTTAaggttAGCAGGCCAAAGATGCTG	Targeting *dctA*18s
*dctA*18sEBS1a	CGGAGTTGCTGTCCCCGTACGCTGActgtttAGCAGCGTATCCAATCC	
*dctA*19sIBS12	AGCCAAAGCAGGTTGACTAGTAAacctctctgtttaGTGCGACGCGAAAGCTAG	
*dctA*19sEBS2s	CGCTAGAAGCCTCGTTAgaggtAGCAGGCCAAAGATGCTG	Targeting *dctA*19s
*dctA*19sEBS1a	CGGAGTTGCTGTCCCCGTACGCTGAtgtttaAGCAGCGTATCCAATCC	
*dctA*146aIBS12	AGCCAAAGCAGGTTGACTAGTAAgatgacaggagcgGTGCGACGCGAAAGCTAG	
*dctA*146aEBS2s	CGCTAGAAGCCTCGTTAtcatcAGCAGGCCAAAGATGCTG	Targeting *dctA*146a
*dctA*146aEBS1a	CGGAGTTGCTGTCCCCGTACGCTGAggagcgAGCAGCGTATCCAATCC	
*dctA*216sIBS12	AGCCAAAGCAGGTTGACTAGTAAggcggtcggtcgtGTGCGACGCGAAAGCTAG	
*dctA*216sEBS2s	CGCTAGAAGCCTCGTTAccgccAGCAGGCCAAAGATGCTG	Targeting *dctA*216s
*dctA*216sEBS1a	CGGAGTTGCTGTCCCCGTACGCTGAggtcgtAGCAGCGTATCCAATCC	
*dctA*227aIBS12	AGCCAAAGCAGGTTGACTAGTAAgtaaagcagtgcgGTGCGACGCGAAAGCTAG	
*dctA*227aEBS2s	CGCTAGAAGCCTCGTTAtttacAGCAGGCCAAAGATGCTG	Targeting *dctA*227a
*dctA*227aEBS1a	CGGAGTTGCTGTCCCCGTACGCTGAagtgcgAGCAGCGTATCCAATCC	
DP-*dctA*-F	CCGCAGGTACCCCATAAC	Detecting *dctA* gene
DP-*dctA*-R	CACTCGGGGAAGGGAGT	
*glcD293a*IBS12	AGCCAAAGCAGGTTGACTAGTAAcacaccttttccGTGCGACGCGAAAGCTAG	
*glcD293a*EBS2s	CGCTAGAAGCCTCGTTAgtgtgAGCAGGCCAAAGATGCTG	Targeting *glcD293a*
*glcD293a*EBS1a	CGGAGTTGCTGTCCCCGTACGCTGAttttccAGCAGCGTATCCAATCC	
*glcD430a*IBS12	AGCCAAAGCAGGTTGACTAGTAAgggtccggtgcgtGTGCGACGCGAAAGCTAG	
*glcD430a*EBS2s	CGCTAGAAGCCTCGTTAgacccAGCAGGCCAAAGATGCTG	Targeting *glcD430a*
*glcD430a*EBS1a	CGGAGTTGCTGTCCCCGTACGCTGAgtgcgtAGCAGCGTATCCAATCC	
*glcD448a*IBS12	AGCCAAAGCAGGTTGACTAGTAAcaggcgatttgtgGTGCG ACGCGAAAGCTAG	
*glcD448a*EBS2s	CGCTAGAAGCCTCGTTAgcctgAGCAGGCCAAAGATGC TG	Targeting *glcD448a*
*glcD448a*EBS1a	CGGAGTTGCTGTCCCCGTACGCTGAtttgtgAGCAGCGTA TCCAATCC	
*glcD531a*IBS12	AGCCAAAGCAGGTTGACTAGTAAttttcagcaggttGTGCGA CGCGAAAGCTAG	Targeting glcD531a

*glcD531a*EBS2s	CGCTAGAAGCCTCGTTAgaaaaAGCAGGCCAAAGATGCTG	
*glcD531a*EBS1a	CGGAGTTGCTGTCCCCGTACGCTGAcaggttAGCAGCGTATCCAATCC	
*glcD555s*IBS12	AGCCAAAGCAGGTTGACTAGTAAaattgaagtgcaaGTGCGACGCGAAAGCTAG	
*glcD555s*EBS2s	CGCTAGAAGCCTCGTTAcaattAGCAGGCCAAAGATGCT G	Targeting *glcD555s*
*glcD555s*EBS1a	CGGAGTTGCTGTCCCCGTACGCTGAgtgcaaAGCAGCGT ATCCAATCC	
*glcD562s*IBS12	AGCCAAAGCAGGTTGACTAGTAAgtgcaaacgctggGTGCG ACGCGAAAGCTAG	
*glcD562s*EBS2s	CGCTAGAAGCCTCGTTAtgcacAGCAGGCCAAAGATGC TG	Targeting *glcD562s*
*glcD562s*EBS1a	CGGAGTTGCTGTCCCCGTACGCTGAcgctggAGCAGCGT ATCCAATCC	
*glcD596a*IBS12	AGCCAAAGCAGGTTGACTAGTAAaccaggtgaatccGTGCG ACGCGAAAGCTAG	
*glcD596a*EBS2s	CGCTAGAAGCCTCGTTActggtAGCAGGCCAAAGATGC TG	Targeting *glcD596a*
*glcD596a*EBS1a	CGGAGTTGCTGTCCCCGTACGCTGAgaatccAGCAGCGTATCCAATCC	
*glcD698a*IBS12	AGCCAAAGCAGGTTGACTAGTAAcagaacccgcgccGTGC GACGCGAAAGCTAG	
*glcD698a*EBS2s	CGCTAGAAGCCTCGTTAttctgAGCAGGCCAAAGATGCT G	Targeting *glcD698a*
*glcD698a*EBS1a	CGGAGTTGCTGTCCCCGTACGCTGAcgcgccAGCAGCGTATCCAATCC	
DP-*glcD*-F	TGGCTGTAATCACACTCGC	Detecting *glcD* gene
DP-*glcD*-R	CAACACAACAAAGCGAAGCC	

*The lowercase letters in the sequence indicate the TMT`s recognition sequence.

Table S2 Strains and plasmids in RGPP.

Strains and plasmids	Characteristics	Resource or reference
**Strains**		
NEBExpress Competent *E. coli* (High efficiency)	*fhuA2* [*Ion*] *ompT gal sulA11 R*(*mcr-73::m*ini*Tn10*--Tet^S)*2*[*dcm*] *R*(*zgb-210::Tn10*--Tet^S)*endA1*delta(*mcrC-mrr*)*114::lS10*	NEB
E. coli HMS174(DE3)	F^- *recA1 hsdR*($r_{K12}^- m_{K12}^+$) (DE3) (Rif^R)	Novogene
Δ*fliC*36s	Derived from *E. coli* HMS174(DE3), Δ*fliC*36s	This study
Δ*fliC*60s	Derived from *E. coli* HMS174(DE3), Δ*fliC*60s	This study
Δ*fliC*67a	Derived from *E. coli* HMS174(DE3), Δ*fliC*67a	This study
Δ*fliC*266s	Derived from *E. coli* HMS174(DE3), Δ*fliC*266s	This study
Δ*fliC*420s	Derived from *E. coli* HMS174(DE3), Δ*fliC*420s	This study
Δ*fliC*434s	Derived from *E. coli* HMS174(DE3), Δ*fliC*434s	This study
Δ*fliC*449s	Derived from *E. coli* HMS174(DE3), Δ*fliC*449s	This study
Δ*fliC*465s	Derived from *E. coli* HMS174(DE3), Δ*fliC*465s	This study
Δ*fliC*489a	Derived from *E. coli* HMS174(DE3), Δ*fliC*489a	This study
Δ*fliC*495s	Derived from *E. coli* HMS174(DE3), Δ*fliC*495s	This study
Δ*fliC*504a	Derived from *E. coli* HMS174(DE3), Δ*fliC*504a	This study
Δ*fliC*511s	Derived from *E. coli* HMS174(DE3), Δ*fliC*511s	This study
Δ*fliC*600s	Derived from *E. coli* HMS174(DE3), Δ*fliC*600s	This study
Δ*fliC*657a	Derived from *E. coli* HMS174(DE3), Δ*fliC*657a	This study
Δ*fliC*828s	Derived from *E. coli* HMS174(DE3), Δ*fliC*828s	This study
Δ*fliC*896a	Derived from *E. coli* HMS174(DE3), Δ*fliC*896a	This study
Δ*fliC*1017a	Derived from *E. coli* HMS174(DE3), Δ*fliC*1017a	This study
Δ*fliC*1038s	Derived from *E. coli* HMS174(DE3), Δ*fliC*1038s	This study
Δ*lacZ*426a	Derived from *E. coli* HMS174(DE3), Δ*lacZ*426a	This study
Δ*lacZ*1155s	Derived from *E. coli* HMS174(DE3), Δ*lacZ*1155s	This study
Δ*lacZ*1879a	Derived from *E. coli* HMS174(DE3), Δ*lacZ*1879a	This study
Δ*lacZ*1880a	Derived from *E. coli* HMS174(DE3), Δ*lacZ*1880a	This study
Δ*dctA*2a	Derived from *E. coli* HMS174(DE3), Δ*dctA*2a	This study
Δ*dctA*227a	Derived from *E. coli* HMS174(DE3), Δ*dctA*227a	This study
Δ*glcD*531a	Derived from *E. coli* HMS174(DE3), Δ*glcD*531a	This study
Δ*glcD*562s	Derived from *E. coli* HMS174(DE3), Δ*glcD*562s	This study
Δ*fliC22s*	Derived from *E. coli* HMS174(DE3), ΔfliC22s	This study
Δ*fliC58s*	Derived from *E. coli* HMS174(DE3), ΔfliC58s	This study
Δ*fliC159a*	Derived from *E. coli* HMS174(DE3), ΔfliC159a	This study
Δ*fliC546s*	Derived from *E. coli* HMS174(DE3), ΔfliC546s	This study
Δ*fliC433s*	Derived from *E. coli* HMS174(DE3), ΔfliC433s	This study
Δ*fliC503a*	Derived from *E. coli* HMS174(DE3), ΔfliC503a	This study
Δ*fliC1302a*	Derived from *E. coli* HMS174(DE3), ΔfliC1302a	This study
Δ*fliC1480a*	Derived from *E. coli* HMS174(DE3), ΔfliC1480a	This study
Δ*fliC1449s*	Derived from *E. coli* HMS174(DE3), ΔfliC1449s	This study
Δ*lacZ136s*	Derived from *E. coli* HMS174(DE3), ΔlacZ136s	This study
Δ*lacZ516a*	Derived from *E. coli* HMS174(DE3), ΔlacZ516a	This study
Δ*lacZ1139a*	Derived from *E. coli* HMS174(DE3), ΔlacZ1139a	This study

Δ*lacZ2586a*	Derived from *E. coli* HMS174(DE3), ΔlacZ2586a	This study
**Plasmids**		
pHK-TT1A	Targetron vector, GroEL promoter, Cm^R	[1]
pHK-TT1A-*fliC*36s	Derived from pHK-TT1A, targeting the sense strand 36 site of *fliC* in HMS174(DE3)	This study
pHK-TT1A-*fliC*60s	Derived from pHK-TT1A, targeting the sense strand 60 site of *fliC* in HMS174(DE3)	This study
pHK-TT1A-*fliC*67a	Derived from pHK-TT1A, targeting the antisense strand 67 site of *fliC* in HMS174(DE3)	This study
pHK-TT1A-*fliC*250s	Derived from pHK-TT1A, targeting the sense strand 250 site of *fliC* in HMS174(DE3)	This study
pHK-TT1A-*fliC*266s	Derived from pHK-TT1A, targeting the sense strand 266 site of *fliC* in HMS174(DE3)	This study
pHK-TT1A-*fliC*420s	Derived from pHK-TT1A, targeting the sense strand 420 site of *fliC* in HMS174(DE3)	This study
pHK-TT1A-*fliC*432s	Derived from pHK-TT1A, targeting the sense strand 432 site of *fliC* in HMS174(DE3)	This study
pHK-TT1A-*fliC*434s	Derived from pHK-TT1A, targeting the sense strand 434 site of *fliC* in HMS174(DE3)	This study
pHK-TT1A-*fliC*435s	Derived from pHK-TT1A, targeting the sense strand 435 site of *fliC* in HMS174(DE3)	This study
pHK-TT1A-*fliC*449s	Derived from pHK-TT1A, targeting the sense strand 449 site of *fliC* in HMS174(DE3)	This study
pHK-TT1A-*fliC*465s	Derived from pHK-TT1A, targeting the sense strand 465 site of *fliC* in HMS174(DE3)	This study
pHK-TT1A-*fliC*489a	Derived from pHK-TT1A, targeting the antisense strand 489 site of *fliC* in HMS174(DE3)	This study
pHK-TT1A-*fliC*495a	Derived from pHK-TT1A, targeting the antisense strand 495 site of *fliC* in HMS174(DE3)	This study
pHK-TT1A-*fliC*495s	Derived from pHK-TT1A, targeting the sense strand 495 site of *fliC* in HMS174(DE3)	This study
pHK-TT1A-*fliC*504a	Derived from pHK-TT1A, targeting the antisense strand 504 site of *fliC* in HMS174(DE3)	This study
pHK-TT1A-*fliC*510a	Derived from pHK-TT1A, targeting the antisense strand 510 site of *fliC* in HMS174(DE3)	This study
pHK-TT1A-*fliC*511s	Derived from pHK-TT1A, targeting the sense strand 511 site of *fliC* in HMS174(DE3)	This study
pHK-TT1A-*fliC*534s	Derived from pHK-TT1A, targeting the sense strand 534 site of *fliC* in HMS174(DE3)	This study
pHK-TT1A-*fliC*600a	Derived from pHK-TT1A, targeting the antisense strand 600 site of *fliC* in HMS174(DE3)	This study
pHK-TT1A-*fliC*600s	Derived from pHK-TT1A, targeting the sense strand 600 site of *fliC* in HMS174(DE3)	This study
pHK-TT1A-*fliC*609s	Derived from pHK-TT1A, targeting the sense strand 609 site of *fliC* in HMS174(DE3)	This study
pHK-TT1A-*fliC*648a	Derived from pHK-TT1A, targeting the antisense strand 648 site of *fliC* in HMS174(DE3)	This study
pHK-TT1A-*fliC*657a	Derived from pHK-TT1A, targeting the antisense strand 657 site of *fliC* in HMS174(DE3)	This study
pHK-TT1A-*fliC*658a	Derived from pHK-TT1A, targeting the antisense strand 658 site of *fliC* in HMS174(DE3)	This study
pHK-TT1A-*fliC*659s	Derived from pHK-TT1A, targeting the sense strand 659 site of *fliC* in HMS174(DE3)	This study
pHK-TT1A-*fliC*702s	Derived from pHK-TT1A, targeting the sense strand 702 site of *fliC* in HMS174(DE3)	This study
pHK-TT1A-*fliC*718s	Derived from pHK-TT1A, targeting the sense strand 718 site of *fliC* in HMS174(DE3)	This study
pHK-TT1A-*fliC*735a	Derived from pHK-TT1A, targeting the antisense strand 735 site of *fliC* in HMS174(DE3)	This study
pHK-TT1A-*fliC*744s	Derived from pHK-TT1A, targeting the sense strand 744 site of *fliC* in HMS174(DE3)	This study
pHK-TT1A-*fliC*768s	Derived from pHK-TT1A, targeting the sense strand 768 site of *fliC* in HMS174(DE3)	This study
pHK-TT1A-*fliC*785s	Derived from pHK-TT1A, targeting the sense strand 785 site of *fliC* in HMS174(DE3)	This study
pHK-TT1A-*fliC*812s	Derived from pHK-TT1A, targeting the sense strand 812 site of *fliC* in HMS174(DE3)	This study
pHK-TT1A-*fliC*828s	Derived from pHK-TT1A, targeting the sense strand 828 site of *fliC* in HMS174(DE3)	This study
pHK-TT1A-*fliC*837s	Derived from pHK-TT1A, targeting the sense strand 837 site of *fliC* in HMS174(DE3)	This study
pHK-TT1A-*fliC*849a	Derived from pHK-TT1A, targeting the antisense strand 849 site of *fliC* in HMS174(DE3)	This study
pHK-TT1A-*fliC*896a	Derived from pHK-TT1A, targeting the antisense strand 896 site of *fliC* in HMS174(DE3)	This study
pHK-TT1A-*fliC*906s	Derived from pHK-TT1A, targeting the sense strand 906 site of *fliC* in HMS174(DE3)	This study
pHK-TT1A-*fliC*930s	Derived from pHK-TT1A, targeting the sense strand 930 site of *fliC* in HMS174(DE3)	This study
pHK-TT1A-*fliC*931s	Derived from pHK-TT1A, targeting the sense strand 931 site of *fliC* in HMS174(DE3)	This study
pHK-TT1A-*fliC*966a	Derived from pHK-TT1A, targeting the antisense strand 966 site of *fliC* in HMS174(DE3)	This study
pHK-TT1A-*fliC*975s	Derived from pHK-TT1A, targeting the sense strand 975 site of *fliC* in HMS174(DE3)	This study
pHK-TT1A-*fliC*1008a	Derived from pHK-TT1A, targeting the antisense strand 1008 site of *fliC* in HMS174(DE3)	This study

pHK-TT1A-*fliC*1017a	Derived from pHK-TT1A, targeting the antisense strand 1017site of *fliC* in HMS174(DE3)	This study
pHK-TT1A-*fliC*1038s	Derived from pHK-TT1A, targeting the sense strand 1038 site of *fliC* in HMS174(DE3)	This study
pHK-TT1A-*fliC*1093s	Derived from pHK-TT1A, targeting the sense strand 1093 site of *fliC* in HMS174(DE3)	This study
pHK-TT1A-*fliC*1114s	Derived from pHK-TT1A, targeting the sense strand 1114 site of *fliC* in HMS174(DE3)	This study
pHK-TT1A-*fliC*1120s	Derived from pHK-TT1A, targeting the sense strand 1120 site of *fliC* in HMS174(DE3)	This study
pHK-TT1A-*fliC*22s	Derived from pHK-TT1A, targeting the sense strand 22 site of *fliC* in HMS174(DE3)	This study
pHK-TT1A-*fliC*58s	Derived from pHK-TT1A, targeting the sense strand 58 site of *fliC* in HMS174(DE3)	This study
pHK-TT1A-*fliC*99a	Derived from pHK-TT1A, targeting the sense strand 99 site of *fliC* in HMS174(DE3)	This study
pHK-TT1A-*fliC*168s	Derived from pHK-TT1A, targeting the sense strand 168 site of *fliC* in HMS174(DE3)	This study
pHK-TT1A-*fliC*159a	Derived from pHK-TT1A, targeting the sense strand 159 site of *fliC* in HMS174(DE3)	This study
pHK-TT1A-*fliC*183s	Derived from pHK-TT1A, targeting the sense strand 183 site of *fliC* in HMS174(DE3)	This study
pHK-TT1A-*fliC213*s	Derived from pHK-TT1A, targeting the sense strand 213 site of *fliC* in HMS174(DE3)	This study
pHK-TT1A-*fliC*364s	Derived from pHK-TT1A, targeting the sense strand 364 site of *fliC* in HMS174(DE3)	This study
pHK-TT1A-*fliC*433s	Derived from pHK-TT1A, targeting the sense strand 433 site of *fliC* in HMS174(DE3)	This study
pHK-TT1A-*fliC*471s	Derived from pHK-TT1A, targeting the sense strand 471 site of *fliC* in HMS174(DE3)	This study
pHK-TT1A-*fliC*503a	Derived from pHK-TT1A, targeting the sense strand 503 site of *fliC* in HMS174(DE3)	This study
pHK-TT1A-*fliC*546s	Derived from pHK-TT1A, targeting the sense strand 546 site of *fliC* in HMS174(DE3)	This study
pHK-TT1A-*fliC*555a	Derived from pHK-TT1A, targeting the sense strand 555 site of *fliC* in HMS174(DE3)	This study
pHK-TT1A-*fliC*594a	Derived from pHK-TT1A, targeting the sense strand 594 site of *fliC* in HMS174(DE3)	This study
pHK-TT1A-*fliC*976s	Derived from pHK-TT1A, targeting the sense strand 976 site of *fliC* in HMS174(DE3)	This study
pHK-TT1A-*fliC*1048a	Derived from pHK-TT1A, targeting the sense strand 1048 site of *fliC* in HMS174(DE3)	This study
pHK-TT1A-*fliC*1132a	Derived from pHK-TT1A, targeting the sense strand 1132 site of *fliC* in HMS174(DE3)	This study
pHK-TT1A-*fliC*1302a	Derived from pHK-TT1A, targeting the sense strand 1302 site of *fliC* in HMS174(DE3)	This study
pHK-TT1A-*fliC*1335s	Derived from pHK-TT1A, targeting the sense strand 1335 site of *fliC* in HMS174(DE3)	This study
pHK-TT1A-*fliC*1341s	Derived from pHK-TT1A, targeting the sense strand 1341 site of *fliC* in HMS174(DE3)	This study
pHK-TT1A-*fliC*1423s	Derived from pHK-TT1A, targeting the sense strand 1423 site of *fliC* in HMS174(DE3)	This study
pHK-TT1A-*fliC*1449s	Derived from pHK-TT1A, targeting the sense strand 1449 site of *fliC* in HMS174(DE3)	This study
pHK-TT1A-*fliC*1463s	Derived from pHK-TT1A, targeting the sense strand 1463 site of *fliC* in HMS174(DE3)	This study
pHK-TT1A-*fliC*1480a	Derived from pHK-TT1A, targeting the sense strand 1480 site of *fliC* in HMS174(DE3)	This study
pHK-TT1A-*lacZ*52s	Derived from pHK-TT1A, targeting the sense strand 52 site of *lacZ* in HMS174(DE3)	This study
pHK-TT1A-*lacZ*168a	Derived from pHK-TT1A, targeting the antisense strand 168 site of *lacZ* in HMS174(DE3)	This study
pHK-TT1A-*lacZ*321s	Derived from pHK-TT1A, targeting the sense strand 321site of *lacZ* in HMS174(DE3)	This study
pHK-TT1A-*lacZ*369a	Derived from pHK-TT1A, targeting the antisense strand 369 site of *lacZ* in HMS174(DE3)	This study
pHK-TT1A-*lacZ*409s	Derived from pHK-TT1A, targeting the sense strand 409 site of *lacZ* in HMS174(DE3)	This study
pHK-TT1A-*lacZ*426a	Derived from pHK-TT1A, targeting the antisense strand 426 site of *lacZ* in HMS174(DE3)	This study
pHK-TT1A-*lacZ*501a	Derived from pHK-TT1A, targeting the antisense strand 501 site of *lacZ* in HMS174(DE3)	This study
pHK-TT1A-*lacZ*515a	Derived from pHK-TT1A, targeting the antisense strand 515 site of *lacZ* in HMS174(DE3)	This study
pHK-TT1A-*lacZ*561s	Derived from pHK-TT1A, targeting the sense strand 561 site of *lacZ* in HMS174(DE3)	This study
pHK-TT1A-*lacZ*629a	Derived from pHK-TT1A, targeting the antisense strand 629 site of *lacZ* in HMS174(DE3)	This study
pHK-TT1A-*lacZ*666s	Derived from pHK-TT1A, targeting the sense strand 666 site of *lacZ* in HMS174(DE3)	This study
pHK-TT1A-*lacZ*906s	Derived from pHK-TT1A, targeting the sense strand 906 site of *lacZ* in HMS174(DE3)	This study
pHK-TT1A-*lacZ*928s	Derived from pHK-TT1A, targeting the sense strand 928 site of *lacZ* in HMS174(DE3)	This study
pHK-TT1A-*lacZ*958s	Derived from pHK-TT1A, targeting the sense strand 958 site of *lacZ* in HMS174(DE3)	This study
pHK-TT1A-*lacZ*1034a	Derived from pHK-TT1A, targeting the antisense strand 1034 site of *lacZ* in HMS174(DE3)	This study
pHK-TT1A-*lacZ*1155s	Derived from pHK-TT1A, targeting the sense strand 1155 site of *lacZ* in HMS174(DE3)	This study

pHK-TT1A-*lacZ*1477s	Derived from pHK-TT1A, targeting the sense strand 1477 site of *lacZ* in HMS174(DE3)	This study
pHK-TT1A-*lacZ*1764a	Derived from pHK-TT1A, targeting the antisense strand 1764 site of *lacZ* in HMS174(DE3)	This study
pHK-TT1A-*lacZ*1811a	Derived from pHK-TT1A, targeting the antisense strand 1811 site of *lacZ* in HMS174(DE3)	This study
pHK-TT1A-*lacZ*1879a	Derived from pHK-TT1A, targeting the antisense strand 1879 site of *lacZ* in HMS174(DE3)	This study
pHK-TT1A-*lacZ*1880a	Derived from pHK-TT1A, targeting the antisense strand 1880 site of *lacZ* in HMS174(DE3)	This study
pHK-TT1A-*lacZ*1927s	Derived from pHK-TT1A, targeting the sense strand 1927 site of *lacZ* in HMS174(DE3)	This study
pHK-TT1A-*lacZ*2068s	Derived from pHK-TT1A, targeting the sense strand 2068 site of *lacZ* in HMS174(DE3)	This study
pHK-TT1A-*lacZ*2262a	Derived from pHK-TT1A, targeting the antisense strand 2262 site of *lacZ* in HMS174(DE3)	This study
pHK-TT1A-*lacZ*2277s	Derived from pHK-TT1A, targeting the sense strand 2277 site of *lacZ* in HMS174(DE3)	This study
pHK-TT1A-*lacZ*2298a	Derived from pHK-TT1A, targeting the antisense strand 2298 site of *lacZ* in HMS174(DE3)	This study
pHK-TT1A-*lacZ*2334s	Derived from pHK-TT1A, targeting the sense strand 2334 site of *lacZ* in HMS174(DE3)	This study
pHK-TT1A-*lacZ*2388s	Derived from pHK-TT1A, targeting the sense strand 2388 site of *lacZ* in HMS174(DE3)	This study
pHK-TT1A-*lacZ*2391a	Derived from pHK-TT1A, targeting the antisense strand 2391 site of *lacZ* in HMS174(DE3)	This study
pHK-TT1A-*lacZ*2407s	Derived from pHK-TT1A, targeting the sense strand 2407 site of *lacZ* in HMS174(DE3)	This study
pHK-TT1A-*lacZ*2473s	Derived from pHK-TT1A, targeting the sense strand 2473 site of *lacZ* in HMS174(DE3)	This study
pHK-TT1A-*lacZ*2496a	Derived from pHK-TT1A, targeting the antisense strand 2496 site of *lacZ* in HMS174(DE3)	This study
pHK-TT1A-*lacZ*2556s	Derived from pHK-TT1A, targeting the sense strand 2556 site of *lacZ* in HMS174(DE3)	This study
pHK-TT1A-*lacZ*2578s	Derived from pHK-TT1A, targeting the sense strand 2578 site of *lacZ* in HMS174(DE3)	This study
pHK-TT1A-*lacZ*2687a	Derived from pHK-TT1A, targeting the antisense strand 2687 site of *lacZ* in HMS174(DE3)	This study
pHK-TT1A-*lacZ*2702s	Derived from pHK-TT1A, targeting the sense strand 2702 site of *lacZ* in HMS174(DE3)	This study
pHK-TT1A-*lacZ*2732a	Derived from pHK-TT1A, targeting the antisense strand 2732 site of *lacZ* in HMS174(DE3)	This study
pHK-TT1A-*lacZ*2733a	Derived from pHK-TT1A, targeting the antisense strand 2733 site of *lacZ* in HMS174(DE3)	This study
pHK-TT1A-*lacZ*2889s	Derived from pHK-TT1A, targeting the sense strand 2889 site of *lacZ* in HMS174(DE3)	This study
pHK-TT1A-*lacZ*2908s	Derived from pHK-TT1A, targeting the sense strand 2908 site of *lacZ* in HMS174(DE3)	This study
pHK-TT1A-*lacZ*2923s	Derived from pHK-TT1A, targeting the sense strand 2923 site of *lacZ* in HMS174(DE3)	This study
pHK-TT1A-*lacZ*2954a	Derived from pHK-TT1A, targeting the antisense strand 2954 site of *lacZ* in HMS174(DE3)	This study
pHK-TT1A-*lacZ*2956s	Derived from pHK-TT1A, targeting the sense strand 2956 site of *lacZ* in HMS174(DE3)	This study
pHK-TT1A-*lacZ*2967a	Derived from pHK-TT1A, targeting the antisense strand 2967 site of *lacZ* in HMS174(DE3)	This study
pHK-TT1A-*lacZ*18a	Derived from pHK-TT1A, targeting the antisense strand 18 site of *lacZ* in HMS174(DE3)	This study
pHK-TT1A-*lacZ*19a	Derived from pHK-TT1A, targeting the antisense strand 19 site of *lacZ* in HMS174(DE3)	This study
pHK-TT1A-*lacZ*60a	Derived from pHK-TT1A, targeting the antisense strand 60 site of *lacZ* in HMS174(DE3)	This study
pHK-TT1A-*lacZ*136s	Derived from pHK-TT1A, targeting the antisense strand 136 site of *lacZ* in HMS174(DE3)	This study
pHK-TT1A-*lacZ*271a	Derived from pHK-TT1A, targeting the antisense strand 271 site of *lacZ* in HMS174(DE3)	This study
pHK-TT1A-*lacZ*410s	Derived from pHK-TT1A, targeting the antisense strand 410 site of *lacZ* in HMS174(DE3)	This study
pHK-TT1A-*lacZ*516a	Derived from pHK-TT1A, targeting the antisense strand 516 site of *lacZ* in HMS174(DE3)	This study
pHK-TT1A-*lacZ*574a	Derived from pHK-TT1A, targeting the antisense strand 574 site of *lacZ* in HMS174(DE3)	This study
pHK-TT1A-*lacZ*679s	Derived from pHK-TT1A, targeting the antisense strand 679 site of *lacZ* in HMS174(DE3)	This study
pHK-TT1A-*lacZ*681a	Derived from pHK-TT1A, targeting the antisense strand 681 site of *lacZ* in HMS174(DE3)	This study
pHK-TT1A-*lacZ*822a	Derived from pHK-TT1A, targeting the antisense strand 822 site of *lacZ* in HMS174(DE3)	This study
pHK-TT1A-*lacZ*897s	Derived from pHK-TT1A, targeting the antisense strand 897 site of *lacZ* in HMS174(DE3)	This study
pHK-TT1A-*lacZ*913s	Derived from pHK-TT1A, targeting the antisense strand 913 site of *lacZ* in HMS174(DE3)	This study
pHK-TT1A-*lacZ*988s	Derived from pHK-TT1A, targeting the antisense strand 988 site of *lacZ* in HMS174(DE3)	This study
pHK-TT1A-*lacZ*1139a	Derived from pHK-TT1A, targeting the antisense strand 1139 site of *lacZ* in HMS174(DE3)	This study
pHK-TT1A-*lacZ*1362s	Derived from pHK-TT1A, targeting the antisense strand Z site of *lacZ* in HMS174(DE3)	This study
pHK-TT1A-*lacZ*1566s	Derived from pHK-TT1A, targeting the antisense strand 1566 site of *lacZ* in HMS174(DE3)	This study
pHK-TT1A-*lacZ*1567s	Derived from pHK-TT1A, targeting the antisense strand 1567 site of *lacZ* in HMS174(DE3)	This study

pHK-TT1A-*lacZ*1580s	Derived from pHK-TT1A, targeting the antisense strand 1580 site of *lacZ* in HMS174(DE3)	This study
pHK-TT1A-*lacZ*1873s	Derived from pHK-TT1A, targeting the antisense strand 1873 site of *lacZ* in HMS174(DE3)	This study
pHK-TT1A-*lacZ*2586a	Derived from pHK-TT1A, targeting the antisense strand 2586 site of *lacZ* in HMS174(DE3)	This study
pHK-TT1A-*lacZ*2725s	Derived from pHK-TT1A, targeting the antisense strand 2725 site of *lacZ* in HMS174(DE3)	This study
pHK-TT1A-*dctA*2a	Derived from pHK-TT1A, targeting the antisense strand 2 site of *dctA* in HMS174(DE3)	This study
pHK-TT1A-*dctA*18a	Derived from pHK-TT1A, targeting the antisense strand 18 site of *dctA* in HMS174(DE3)	This study
pHK-TT1A-*dctA*18s	Derived from pHK-TT1A, targeting the sense strand 18 site of *dctA* in HMS174(DE3)	This study
pHK-TT1A-*dctA*19s	Derived from pHK-TT1A, targeting the sense strand 19 site of *dctA* in HMS174(DE3)	This study
pHK-TT1A-*dctA*146a	Derived from pHK-TT1A, targeting the antisense strand 146 site of *dctA* in HMS174(DE3)	This study
pHK-TT1A-*dctA*216s	Derived from pHK-TT1A, targeting the sense strand 216 site of *dctA* in HMS174(DE3)	This study
pHK-TT1A-*dctA*227a	Derived from pHK-TT1A, targeting the antisense strand 227 site of *dctA* in HMS174(DE3)	This study
pHK-TT1A-*glcD*293a	Derived from pHK-TT1A, targeting the antisense strand 293 site of *glcD* in HMS174(DE3)	This study
pHK-TT1A-*glcD*430a	Derived from pHK-TT1A, targeting the antisense strand 430 site of *glcD* in HMS174(DE3)	This study
pHK-TT1A-*glcD*448a	Derived from pHK-TT1A, targeting the antisense strand 448 site of *glcD* in HMS174(DE3)	This study
pHK-TT1A-*glcD*531a	Derived from pHK-TT1A, targeting the antisense strand 531 site of *glcD* in HMS174(DE3)	This study
pHK-TT1A-*glcD*555s	Derived from pHK-TT1A, targeting the sense strand 555 site of *glcD* in HMS174(DE3)	This study
pHK-TT1A- *glcD*562s	Derived from pHK-TT1A, targeting the sense strand 562 site of *glcD* in HMS174(DE3)	This study
pHK-TT1A-*glcD*596a	Derived from pHK-TT1A, targeting the antisense strand 596 site of *glcD* in HMS174(DE3)	This study
pHK-TT1A-*glcD*698a	Derived from pHK-TT1A, targeting the antisense strand 698 site of *glcD* in HMS174(DE3)	This study

Table S3 Gene-targeting efficiencies of 152 plasmids in the RGPP.

No.*	Targeting Sites	Recognition Sequences (-13 to -1)	Targeting Efficiency
1	*fliC*36s	CAGCCTCTCGCTG	5%
2	*fliC*60s	TAATATCAACAAG	29%
3	*fliC*250s	GGCGCGCTGTCCG	0%
4	*fliC*266s	TCAACAACAACTT	57%
5	*fliC*420s	CGTGCTGGCAAAA	86%
6	*fliC432s*	AAATGGCTCCATG	0%
7	*fliC*434s	ATGGCTCCATGAA	18%
8	*fliC*435s	TGGCTCCATGAAA	0%
9	*fliC*449s	TCCAGGTTGGCGC	18%
10	*fliC*465s	TGATAACCAGACT	40%
11	*fliC*495s	GCAGATTGATGCT	50%
12	*fliC*511s	ACTCTTGGCCTTG	100%
13	*fliC*543s	TAACGATACAGTT	0%
14	*fliC*600s	CAATATTAAACTT	81%
15	*fliC*609s	ACTTACTGGAATT	0%
16	*fliC*659s	CTAACCCAGCTTC	0%
17	*fliC*702s	TGATTACTATGCG	0%
18	*fliC*718s	ATCACCGGTGGTG	0%
19	*fliC*744s	GTATTACGCAGTA	0%
20	*fliC*768s	TGATGGTACAGTG	0%
21	*fliC*785s	TGGCGACTGGAGC	0%
22	*fliC*812s	CTGTAACTGATGC	0%
23	*fliC*828s	TACTACTAAAGCT	100%
24	*fliC*837s	AGCTACAACTATC	0%
25	*fliC*906s	CCTTGGTGCTGTT	0%
26	*fliC*930s	ACTGCAGGATTCC	0%
27	*fliC*931s	CTGCAGGATTCCA	0%
28	*fliC*975s	AGATACAAATGGC	0%
29	*fliC*1038s	AACTATTACCTAT	100%
30	*fliC*1093s	CTGGGCGGAGATG	0%
31	*fliC*1114s	ACAGAAGTGGTCG	0%
32	*fliC*1120s	GTGGTCGATATTG	0%
33	*fliC*67a	CTCGACAGCGCAG	9%
34	*fliC*489a	GAGTTTTAGCATC	100%
35	*fliC*495a	GGCCAAGAGTTTT	0%
36	*fliC*504a	AACCATCAAGGCC	95%
37	*fliC*510a	CGCTAAAACCATC	0%
38	*fliC*600a	GGGTAATTCCAGT	0%
39	*fliC*648a	TTGAAGCTGGGTT	0%
40	*fliC*657a	CACCCTCAATTGA	5%
41	*fliC*658a	ACACCCTCAATTG	0%
42	*fliC*735a	CTGTTACTGCGTA	0%

43	*fliC*849a	TCTGAACAGGTGT	0%
44	*fliC*896a	GCTAACAGCACCA	73%
45	*fliC*966a	GATTGCCATTTGT	0%
46	*fliC*1008a	CAGAAACAGCACC	0%
47	*fliC*1017a	TAGTTTTAACAGA	100%
48	*fliC*22s	GTCATTAATACCA	17%
49	*fliC*58s	AATAATATCAACA	33%
50	*fliC*99a	TACGCAAGCCAGA	0%
51	*fliC*168s	CCGTTTCACCTCT	0%
52	*fliC*159a	TGTTAGAGGTGAA	100%
53	*fliC*183s	CATTAAAGGCCTG	0%
54	*fliC*213s	CGCCAACGACGGT	0%
55	*fliC*364s	TCCCGTCTGGATG	0%
56	*fliC*433s	AATGGCTCCATGA	8%
57	*fliC*471s	CCAGACTATCACT	0%
58	*fliC*503a	ACCATCAAGGCCA	38%
59	*fliC*546s	CGATACAGTTACC	67%
60	*fliC*555a	AAGCAGTTACTGG	0%
61	*fliC*594a	TTCCAGTAAGTTT	0%
62	*fliC*976s	GATACAAATGGCA	0%
63	*fliC*1048a	CTGGCGGCACCGG	0%
64	*fliC*1132a	TCGGCAGAATCGT	0%
65	*fliC*1302a	CCGCGGAATCCAG	25%
66	*fliC*1335s	CCTGAACAACACC	0%
67	*fliC*1341s	CAACACCACTACC	0%
68	*fliC*1423s	GCGCAGATCATCC	0%
69	*fliC*1449s	CTCCGTGTTGGCA	75%
70	*fliC*1463s	AAGCTAACCAGGT	0%
71	*fliC*1480a	CCCTGCAGCAGAG	25%
72	*lacZ*52s	CGTCGTGACTGGG	0%
73	*lacZ*321s	CGTGACCTATCCC	0%
74	*lacZ*409s	AGCTGGCTACAGG	0%
75	*lacZ*561s	CCGCCTCGCGGTG	0%
76	*lacZ*666s	ACCGACTACACAA	0%
77	*lacZ*906s	CCCGAAACTGTGG	0%
78	*lacZ*928s	ATCCCGAATCTCT	0%
79	*lacZ*958s	CTGCACACCGCCG	0%
80	*lacZ*1155s	GCAGAACAACTTT	75%
81	*lacZ*1477s	GGCGGCGGAGCCG	0%
82	*lacZ*1927s	GTGACCAGCGAAT	0%
83	*lacZ*2068s	CTACCGCAGCCGG	0%
84	*lacZ*2277s	GCGTTGGCAATTT	0%
85	*lacZ*2334s	AAAACAACTGCTG	0%
86	*lacZ*2388s	CGACATTGGCGTA	0%
87	*lacZ*2407s	GCGACCCGCATTG	0%
88	*lacZ*2473s	GCAGCGTTGTTGC	0%

89	*lacZ*2556s	AACCTTATTTATC	0%
90	*lacZ*2578s	ACCTACCGGATTG	0%
91	*lacZ*2702s	ACTGGCTCGGATT	0%
92	*lacZ*2889s	CATCAGCCGCTAC	0%
93	*lacZ*2908s	CAGCAACTGATGG	0%
94	*lacZ*2923s	ACCAGCCATCGCC	0%
95	*lacZ*2956s	GGCACATGGCTGA	0%
96	*lacZ*168a	AGCGCCATTCGCC	0%
97	*lacZ*369a	ATGTGAGCGAGTA	0%
98	*lacZ*426a	CGCCATCAAAAAT	60%
99	*lacZ*501a	ATTCAGACGGCAA	0%
100	*lacZ*515a	TGCGCTCAGGTCA	0%
101	*lacZ*629a	CGAGACGTCACGG	0%
102	*lacZ*1034a	CGGCTTGCCGTTC	0%
103	*lacZ*1764a	AATCACCGCCGTA	0%
104	*lacZ*1811a	GACCAGACCGTTC	0%
105	*lacZ*1879a	CGGAACTGGAAAA	75%
106	*lacZ*1880a	ACGGAACTGGAAA	60%
107	*lacZ*2262a	ATTGCCAACGCTT	0%
108	*lacZ*2298a	TCCACATCTGTGA	0%
109	*lacZ*2391a	TGCGGGTCGCTTC	0%
110	*lacZ*2496a	TCAGCACCGCATC	0%
111	*lacZ*2687a	TCCGAGCCAGTTT	0%
112	*lacZ*2732a	ACAGGCGGCAGTA	0%
113	*lacZ*2733a	AACAGGCGGCAGT	0%
114	*lacZ*2954a	ACCGTCGATATTC	0%
115	*lacZ*2967a	TCCCCATATGGAA	0%
116	*lacZ*18a	CGACGGCCAGTGA	0%
117	*lacZ*19a	ACGACGGCCAGTG	0%
118	*lacZ*60a	GTTGGGTAACGCC	0%
119	*lacZ*136s	GAGGCCCGCACCG	8%
120	*lacZ*271a	CCGTGCATCTGCC	0%
121	*lacZ*410s	GCTGGCTACAGGA	0%
122	*lacZ*516a	ATGCGCTCAGGTC	83%
123	*lacZ*574a	CTGCCGTCACTCC	0%
124	*lacZ*679s	ATCAGCGATTTCC	0%
125	*lacZ*681a	AGCGAGTGGCAAC	0%
126	*lacZ*822a	TTTCACCGCCGAA	0%
127	*lacZ*897s	CGTCGAAAACCCG	0%
128	*lacZ*913s	CTGTGGAGCGCCG	0%
129	*lacZ*988s	GCAGAAGCCTGCG	0%
130	*lacZ*1139a	GTTGTTCTGCTTC	8%
131	*lacZ*1362s	TCACCCGAGTGTG	0%
132	*lacZ*1566s	ATGGTCCATCAAA	0%
133	*lacZ*1567s	TGGTCCATCAAAA	0%
134	*lacZ*1580s	AATGGCTTTCGCT	0%

135	*lacZ*1873s	GCAAAACACCAGC	0%
136	*lacZ*2586a	TCGCCATTTGACC	80%
137	*lacZ*2725s	GAAAACTATCCCG	0%
138	*dctA*18s	AACCTCTCTGTTT	0%
139	*dctA*19s	ACCTCTCTGTTTA	0%
140	*dctA*216s	GGCGGTCGGTCGT	0%
141	*dctA*2a	CAGAGAGGTTTTC	100%
142	*dctA*18a	AGTAAAGGCTTTT	0%
143	*dctA*146a	GATGACAGGAGCG	0%
144	*dctA*227a	GTAAAGCAGTGCG	5%
145	*glcD*555s	AATTGAAGTGCAA	0%
146	*glcD*562s	GTGCAAACGCTGG	100%
147	*glcD*293a	CACACCTTTTTCC	0%
148	*glcD*430a	GGGTCCGGTGCGT	0%
149	*glcD*448a	CAGGCGATTTGTG	0%
150	*glcD*531a	TTTTCAGCAGGTT	100
151	*glcD*596a	ACCAGGTGAATCC	0%
152	*glcD*698a	CAGAACCCGCGCC	0%

*A total of 152 targeting sites were randomly selected. 's': sense strand; 'a': antisense strand. The 13 bases (from -13 to -1) are the key bases for targets recognition. Twenty-four colonies were randomly picked for PCR validation, and the targeting efficiency is calculated as follows: gene-targeting efficiency = [(gene-inactivated colonies) / (total detected colonies)] × 100%.

Table S4 Primers used in the redesigned targets (including 13 plasmids with $T_{(-8)}$ and 18 plasmids with $A/C/G_{(-8)}$ at EBS2b site).

**Primers**	**Sequences (5′ -3′) ***	**Note**
TeI3c-UNV	TAACGAGGCTTCTAGCG	Universal primer
DP-*ptsG*-F	CCCATACTCAGGAGCACTCT	Detecting *ptsG* gene
DP-*ptsG*-R	CCTTAGTCTCCCCAACGTCT	
DP-*qseC*-F	CGTACCGTGCATGGTATTGG	Detecting *qseC* gene
DP-*qseC*-R	GAGTCGTTTTAACGGCTCTCA	
IBS2b site is T base		
*qseC*38aIBS12	AGCCAAAGCAGGTTGACTAGTAAaaagattagcgtcGTGCGACGCGAAAGCTAG	
*qseC*38aEBS2s	CGCTAGAAGCCTCGTTAtctttAGCAGGCCAAAGATGCTG	Targeting *qseC*38a
*qseC*38aEBS1a	CGGAGTTGCTGTCCCCGTACGCTGAagcgtcAGCAGCGTATCCAATCC	
*qseC*244sIBS12	AGCCAAAGCAGGTTGACTAGTAAcacggtcatgttgGTGCGACGCGAAAGCTAG	
*qseC*244sEBS2s	CGCTAGAAGCCTCGTTAccgtgAGCAGGCCAAAGATGCTG	Targeting *qseC*244s
*qseC*244sEBS1a	CGGAGTTGCTGTCCCCGTACGCTGAatgttgAGCAGCGTATCCAATCC	
*qseC*309aIBS12	AGCCAAAGCAGGTTGACTAGTAAtatcttctccgttGTGCGACGCGAAAGCTAG	
*qseC*309aEBS2s	CGCTAGAAGCCTCGTTAagataAGCAGGCCAAAGATGCTG	Targeting *qseC*309a
*qseC*309aEBS1a	CGGAGTTGCTGTCCCCGTACGCTGAtccgttAGCAGCGTATCCAATCC	
*qseC*627sIBS12	AGCCAAAGCAGGTTGACTAGTAAaccactaaacgcgGTGCGACGCGAAAGCTAG	
*qseC*627sEBS2s	CGCTAGAAGCCTCGTTAgtggtAGCAGGCCAAAGATGCTG	Targeting *qseC*627s
*qseC*627sEBS1a	CGGAGTTGCTGTCCCCGTACGCTGAaacgcgAGCAGCGTATCCAATCC	
*qseC*1052aIBS12	AGCCAAAGCAGGTTGACTAGTAAcaaacttagcaatGTGCGACGCGAAAGCTAG	
*qseC*1052aEBS2s	CGCTAGAAGCCTCGTTAgtttgAGCAGGCCAAAGATGCTG	Targeting *qseC*1052a
*qseC*1052aEBS1a	CGGAGTTGCTGTCCCCGTACGCTGAagcaatAGCAGCGTATCCAATCC	
*qseC*1073sIBS12	AGCCAAAGCAGGTTGACTAGTAAgtttgttggtgcgGTGCGACGCGAAAGCTAG	
*qseC*1073sEBS2s	CGCTAGAAGCCTCGTTAcaaacAGCAGGCCAAAGATGCTG	Targeting *qseC*1073s
*qseC*1073sEBS1a	CGGAGTTGCTGTCCCCGTACGCTGAggtgcgAGCAGCGTATCCAATCC	
*qseC*1294sIBS12	AGCCAAAGCAGGTTGACTAGTAAttgcatggcatgaGTGCGACGCGAAAGCTAG	
*qseC*1294sEBS2s	CGCTAGAAGCCTCGTTAtgcaaAGCAGGCCAAAGATGCTG	Targeting *qseC*1294s
*qseC*1294sEBS1a	CGGAGTTGCTGTCCCCGTACGCTGAgcatgaAGCAGCGTATCCAATCC	
*qseC*1330sIBS12	AGCCAAAGCAGGTTGACTAGTAAcaaggtggatttgGTGCGACGCGAAAGCTAG	
*qseC*1330sEBS2s	CGCTAGAAGCCTCGTTAccttgAGCAGGCCAAAGATGCTG	Targeting *qseC*1330s
*qseC*1330sEBS1a	CGGAGTTGCTGTCCCCGTACGCTGAgatttgAGCAGCGTATCCAATCC	
*ptsG*345sIBS12	AGCCAAAGCAGGTTGACTAGTAAacacctggcggatGTGCGACGCGAAAGCTAG	
*ptsG*345sEBS2s	CGCTAGAAGCCTCGTTAggtgtAGCAGGCCAAAGATGCTG	Targeting *ptsG*345s
*ptsG*345sEBS1a	CGGAGTTGCTGTCCCCGTACGCTGAgcggatAGCAGCGTATCCAATCC	
*ptsG*1083aIBS12	AGCCAAAGCAGGTTGACTAGTAAtcgcataaccgatGTGCGACGCGAAAGCTAG	
*ptsG*1083aEBS2s	CGCTAGAAGCCTCGTTAtgcgaAGCAGGCCAAAGATGCTG	Targeting *ptsG*1083a
*ptsG*1083aEBS1a	CGGAGTTGCTGTCCCCGTACGCTGAaccgatAGCAGCGTATCCAATCC	
*ptsG*1230aIBS12	AGCCAAAGCAGGTTGACTAGTAAtgttttctttaccGTGCGACGCGAAAGCTAG	
*ptsG*1230aEBS2s	CGCTAGAAGCCTCGTTAaaacaAGCAGGCCAAAGATGCTG	Targeting *ptsG*1230a
*ptsG*1230aEBS1a	CGGAGTTGCTGTCCCCGTACGCTGAtttaccAGCAGCGTATCCAATCC	
*ptsG*1359aIBS12	AGCCAAAGCAGGTTGACTAGTAAtcgcctgaacaccGTGCGACGCGAAAGCTAG	Targeting *ptsG*1359a
*ptsG*1359aEBS2s	CGCTAGAAGCCTCGTTAggcgaAGCAGGCCAAAGATGCTG	

*ptsG*1359aEBS1a	CGGAGTTGCTGTCCCCGTACGCTGAaacaccAGCAGCGTATCCAATCC	
*ptsG*1399sIBS12	AGCCAAAGCAGGTTGACTAGTAAtccgataacctgaGTGCGACGCGAAAGCTAG	
*ptsG*1399sEBS2s	CGCTAGAAGCCTCGTTAtcggaAGCAGGCCAAAGATGCTG	Targeting *ptsG*1399s
*ptsG*1399sEBS1a	CGGAGTTGCTGTCCCCGTACGCTGAacctgaAGCAGCGTATCCAATCC	
IBS2b site is A/G/C bases		
*ptsG*498aIBS12	AGCCAAAGCAGGTTGACTAGTAAtgaaggacagcacGTGCGACGCGAAAGCTAG	
*ptsG*498aEBS2s	CGCTAGAAGCCTCGTTActtcaAGCAGGCCAAAGATGCTG	Targeting *ptsG*498a
*ptsG*498aEBS1a	CGGAGTTGCTGTCCCCGTACGCTGAcagcacAGCAGCGtATCCAATCC	
*ptsG*785sIBS12	AGCCAAAGCAGGTTGACTAGTAAtgtacggtctgccGTGCGACGCGAAAGCTAG	
*ptsG*785sEBS2s	CGCTAGAAGCCTCGTTAgtacaAGCAGGCCAAAGATGCTG	Targeting *ptsG*785s
*ptsG*785sEBS1a	CGGAGTTGCTGTCCCCGTACGCTGAtctgccAGCAGCGTATCCAATCC	
*ptsG*849sIBS12	AGCCAAAGCAGGTTGACTAGTAAagtgggcggtattGTGCGACGCGAAAGCTAG	
*ptsG*849sEBS2s	CGCTAGAAGCCTCGTTAccactAGCAGGCCAAAGATGCTG	Targeting *ptsG*849s
*ptsG*849sEBS1a	CGGAGTTGCTGTCCCCGTACGCTGAggtattAGCAGCGTATCCAATCC	
*qseC*568sIBS12	AGCCAAAGCAGGTTGACTAGTAActcgcgccgctgaGTGCGACGCGAAAGCTAG	
*qseC*568sEBS2s	CGCTAGAAGCCTCGTTAgcgagAGCAGGCCAAAGATGCTG	Targeting *qseC*568
*qseC*568sEBS1a	CGGAGTTGCTGTCCCCGTACGCTGAcgctgaAGCAGCGTATCCAATCC	
*qseC*704aIBS12	AGCCAAAGCAGGTTGACTAGTAAgcgtcgttcacgaGTGCGACGCGAAAGCTAG	
*qseC*704aEBS2s	CGCTAGAAGCCTCGTTAgacgcAGCAGGCCAAAGATGCTG	Targeting *qseC*704a
*qseC*704aEBS1a	CGGAGTTGCTGTCCCCGTACGCTGAtcacgaAGCAGCGtATCCAATCC	
*qseC*967sIBS12	AGCCAAAGCAGGTTGACTAGTAAtcgtcggtgatggGTGCGACGCGAAAGCTAG	
*qseC*967sEBS2s	CGCTAGAAGCCTCGTTAgacgaAGCAGGCCAAAGATGCTG	Targeting *qseC*967s
*qseC*967sEBS1a	CGGAGTTGCTGTCCCCGTACGCTGAtgatggAGCAGCGTATCCAATCC	
*ptsG*198aIBS12	AGCCAAAGCAGGTTGACTAGTAAagccgagggcgacGTGCGACGCGAAAGCTAG	
*ptsG*198aEBS2s	CGCTAGAAGCCTCGTTAcggctAGCAGGCCAAAGATGCTG	Targeting *ptsG*198a
*ptsG*198aEBS1a	CGGAGTTGCTGTCCCCGTACGCTGAggcgacAGCAGCGtATCCAATCC	
*ptsG*388aIBS12	AGCCAAAGCAGGTTGACTAGTAAcggttaaacatgtGTGCGACGCGAAAGCTAG	
*ptsG*388aEBS2s	CGCTAGAAGCCTCGTTAaaccgAGCAGGCCAAAGATGCTG	Targeting *ptsG*388a
*ptsG*388aEBS1a	CGGAGTTGCTGTCCCCGTACGCTGAacatgtAGCAGCGtATCCAATCC	
*ptsG*584aIBS12	AGCCAAAGCAGGTTGACTAGTAAaccgtaaatgccaGTGCGACGCGAAAGCTAG	
*ptsG*584aEBS2s	CGCTAGAAGCCTCGTTAacggtAGCAGGCCAAAGATGCTG	Targeting *ptsG*584a
*ptsG*584aEBS1a	CGGAGTTGCTGTCCCCGTACGCTGAatgccaAGCAGCGtATCCAATCC	
*qseC*115sIBS12	AGCCAAAGCAGGTTGACTAGTAAcaaacaacggataGTGCGACGCGAAAGCTAG	
*qseC*115sEBS2s	CGCTAGAAGCCTCGTTAgtttgAGCAGGCCAAAGATGCTG	Targeting *qseC*115s
*qseC*115sEBS1a	CGGAGTTGCTGTCCCCGTACGCTGAcggataAGCAGCGTATCCAATCC	
*qseC*457sIBS12	AGCCAAAGCAGGTTGACTAGTAAtgggaataccgtgGTGCGACGCGAAAGCTAG	
*qseC*457sEBS2s	CGCTAGAAGCCTCGTTAtcccaAGCAGGCCAAAGATGCTG	Targeting *qseC*457s
*qseC*457sEBS1a	CGGAGTTGCTGTCCCCGTACGCTGAaccgtgAGCAGCGTATCCAATCC	
*qseC*539sIBS12	AGCCAAAGCAGGTTGACTAGTAAtcatcatgatggtGTGCGACGCGAAAGCTAG	
*qseC*539sEBS2s	CGCTAGAAGCCTCGTTAgatgaAGCAGGCCAAAGATGCTG	Targeting *qseC*539a
*qseC*539sEBS1a	CGGAGTTGCTGTCCCCGTACGCTGAgatggtAGCAGCGTATCCAATCC	
*ptsG*21sIBS12	AGCCAAAGCAGGTTGACTAGTAAgaatgcatttgctGTGCGACGCGAAAGCTAG	
*ptsG*21sEBS2s	CGCTAGAAGCCTCGTTAcattcAGCAGGCCAAAGATGCTG	Targeting *ptsG*21s
*ptsG*21sEBS1a	CGGAGTTGCTGTCCCCGTACGCTGAtttgctAGCAGCGTATCCAATCC	
*ptsG*95aIBS12	AGCCAAAGCAGGTTGACTAGTAAattcgcggaaccgGTGCGACGCGAAAGCTAG	
*ptsG*95aEBS2s	CGCTAGAAGCCTCGTTAcgaatAGCAGGCCAAAGATGCTG	Targeting *ptsG*95a

*ptsG*95aEBS1a	CGGAGTTGCTGTCCCCGTACGCTGAgaaccgAGCAGCGtATCCAATCC	
*ptsG*514aIBS12	AGCCAAAGCAGGTTGACTAGTAAccaatcggcggccGTGCGACGCGAAAGCTAG	
*ptsG*514aEBS2s	CGCTAGAAGCCTCGTTAattggAGCAGGCCAAAGATGCTG	Targeting *ptsG*514a
*ptsG*514aEBS1a	CGGAGTTGCTGTCCCCGTACGCTGAgcggccAGCAGCGtATCCAATCC	
*qseC*139sIBS12	AGCCAAAGCAGGTTGACTAGTAAttgttcgacacccGTGCGACGCGAAAGCTAG	
*qseC*139sEBS2s	CGCTAGAAGCCTCGTTAaacaaAGCAGGCCAAAGATGCTG	Targeting *qseC*139s
*qseC*139sEBS1a	CGGAGTTGCTGTCCCCGTACGCTGAacacccAGCAGCGTATCCAATCC	
*qseC*257aIBS12	AGCCAAAGCAGGTTGACTAGTAAgatggcaaaggtcGTGCGACGCGAAAGCTAG	
*qseC*257aEBS2s	CGCTAGAAGCCTCGTTAccatcAGCAGGCCAAAGATGCTG	Targeting *qseC*257a
*qseC*257aEBS1a	CGGAGTTGCTGTCCCCGTACGCTGAaaggtcAGCAGCGtATCCAATCC	
*qseC*476aIBS12	AGCCAAAGCAGGTTGACTAGTAAttgcccggcaacaGTGCGACGCGAAAGCTAG	
*qseC*476aEBS2s	CGCTAGAAGCCTCGTTAggcaaAGCAGGCCAAAGATGCTG	Targeting *qseC*476a
*qseC*476aEBS1a	CGGAGTTGCTGTCCCCGTACGCTGAgcaacaAGCAGCGtATCCAATCC	

*The lowercase letters in the sequence indicate the recognition sequences.

Table S5 Strains and plasmids used in the redesigned targets (including 13 plasmids with $T_{(-8)}$ and 18 plasmids with $A/C/G_{(-8)}$ at EBS2b site).

**Strains and plasmids**	**Characteristics**	**Resource or reference**
**Strains**		
NEBExpress Competent *E. coli* (High efficiency)	*fhuA2* [*Ion*] *ompT gal sulA11 R*(*mcr-73::m*ini*Tn10*--Tet^{S})*2*[*dcm*] *R*(*zgb-210::Tn10*--Tet^{S})*endA1*delta(*mcrC-mrr*)*114::IS10*	NEB
E. coli HMS174(DE3)	F^{-} *recA1 hsdR*($r_{K12}^{-}m_{K12}^{+}$) (DE3) (Rif^{R})	Novogene
IBS2b site is T base		
Δ*qseC*38a	Derived from *E. coli* HMS174(DE3), Δ*qseC*38a	This study
Δ*qseC*244s	Derived from *E. coli* HMS174(DE3), Δ*qseC*244s	This study
Δ*qseC*309a	Derived from *E. coli* HMS174(DE3), Δ*qseC*309a	This study
Δ*qseC*627s	Derived from *E. coli* HMS174(DE3), Δ*qseC*627s	This study
Δ*qseC*1052a	Derived from *E. coli* HMS174(DE3), Δ*qseC*1052a	This study
Δ*qseC*1073s	Derived from *E. coli* HMS174(DE3), Δ*qseC*1073s	This study
Δ*qseC*1294s	Derived from *E. coli* HMS174(DE3), Δ*qseC*1294s	This study
Δ*qseC*1330s	Derived from *E. coli* HMS174(DE3), Δ*qseC*1330s	This study
Δ*ptsG*1230a	Derived from *E. coli* HMS174(DE3), Δ*ptsG* 1230a	This study
Δ*ptsG*1359a	Derived from *E. coli* HMS174(DE3), Δ*ptsG* 1359a	This study
Δ*ptsG*1399s	Derived from *E. coli* HMS174(DE3), Δ*ptsG*1399s	This study
IBS2b site is not T base		
Δ*ptsG*849s	Derived from *E. coli* HMS174(DE3), Δ*ptsG*849s	This study
Δ*ptsG*584a	Derived from *E. coli* HMS174(DE3), Δ*ptsG*584a	This study
Δ*ptsG*21s	Derived from *E. coli* HMS174(DE3), Δ*ptsG*21s	This study
**Plasmids**		
pHK-TT1A	Targetron vector, GroEL promoter, Cm^{R}	[1]
IBS2b site is T base		
pHK-TT1A-*qseC*38a	Derived from pHK-TT1A, targeting the antisense strand 38 site of *qseC* in HMS174(DE3)	This study
pHK-TT1A-*qseC*244s	Derived from pHK-TT1A, targeting the sense strand 244 site of *qseC* in HMS174(DE3)	This study
pHK-TT1A-*qseC*309a	Derived from pHK-TT1A, targeting the antisense strand 309 site of *qseC* in HMS174(DE3)	This study
pHK-TT1A-*qseC*627s	Derived from pHK-TT1A, targeting the sense strand 627 site of *qseC* in HMS174(DE3)	This study
pHK-TT1A-*qseC*1052a	Derived from pHK-TT1A, targeting the antisense strand 1052 site of *qseC* in HMS174(DE3)	This study
pHK-TT1A-*qseC*1073s	Derived from pHK-TT1A, targeting the sense strand 1073 site of *qseC* in HMS174(DE3)	This study
pHK-TT1A-*qseC*1294s	Derived from pHK-TT1A, targeting the sense strand 1294 site of *qseC* in HMS174(DE3)	This study
pHK-TT1A-*qseC*1330s	Derived from pHK-TT1A, targeting the sense strand 1330 site of *qseC* in HMS174(DE3)	This study
pHK-TT1A-*ptsG*345s	Derived from pHK-TT1A, targeting the sense strand 345 site of *ptsG* in HMS174(DE3)	This study
pHK-TT1A-*ptsG*1083a	Derived from pHK-TT1A, targeting the antisense strand 1083 site of *ptsG* in HMS174(DE3)	This study
pHK-TT1A-*ptsG*1230a	Derived from pHK-TT1A, targeting the antisense strand 1230 site of *ptsG* in HMS174(DE3)	This study
pHK-TT1A-*ptsG*1359a	Derived from pHK-TT1A, targeting the antisense strand 1359 site of *ptsG* in HMS174(DE3)	This study
pHK-TT1A-*ptsG*1399s	Derived from pHK-TT1A, targeting the sense strand 1399 site of *ptsG* in HMS174(DE3)	This study
IBS2b site are A/C/G bases		
pHK-TT1A-*ptsG*498a	Derived from pHK-TT1A, targeting the antisense strand 498 site of *qseC* in HMS174(DE3)	This study
pHK-TT1A-	Derived from pHK-TT1A, targeting the antisense strand 785 site of *qseC* in HMS174(DE3)	This study

*ptsG*785s		
pHK-TT1A-*ptsG*849s	Derived from pHK-TT1A, targeting the antisense strand 849 site of *qseC* in HMS174(DE3)	This study
pHK-TT1A-*qseC*568s	Derived from pHK-TT1A, targeting the sense strand 568 site of *qseC* in HMS174(DE3)	This study
pHK-TT1A-*qseC*704a	Derived from pHK-TT1A, targeting the sense strand 704 site of *qseC* in HMS174(DE3)	This study
pHK-TT1A-*qseC*967s	Derived from pHK-TT1A, targeting the sense strand 967 site of *qseC* in HMS174(DE3)	This study
pHK-TT1A-*ptsG*198a	Derived from pHK-TT1A, targeting the antisense strand 198 site of *ptsG* in HMS174(DE3)	This study
pHK-TT1A-*ptsG*388a	Derived from pHK-TT1A, targeting the antisense strand 388 site of *ptsG* in HMS174(DE3)	This study
pHK-TT1A-*ptsG*584a	Derived from pHK-TT1A, targeting the antisense strand 584 site of *ptsG* in HMS174(DE3)	This study
pHK-TT1A-*qseC*115s	Derived from pHK-TT1A, targeting the antisense strand 115 site of *ptsG* in HMS174(DE3)	This study
pHK-TT1A-*qseC*457s	Derived from pHK-TT1A, targeting the sense strand 457site of *ptsG* in HMS174(DE3)	This study
pHK-TT1A-*qseC*539a	Derived from pHK-TT1A, targeting the sense strand 539 site of *ptsG* in HMS174(DE3)	This study
pHK-TT1A-*ptsG*21s	Derived from pHK-TT1A, targeting the sense strand 21 site of *ptsG* in HMS174(DE3)	This study
pHK-TT1A-*ptsG*95a	Derived from pHK-TT1A, targeting the sense strand 95 site of *ptsG* in HMS174(DE3)	This study
pHK-TT1A-*ptsG*514a	Derived from pHK-TT1A, targeting the sense strand 514 site of *ptsG* in HMS174(DE3)	This study
pHK-TT1A-*qseC*139s	Derived from pHK-TT1A, targeting the sense strand 139 site of *ptsG* in HMS174(DE3)	This study
pHK-TT1A-*qseC*257a	Derived from pHK-TT1A, targeting the sense strand 257 site of *ptsG* in HMS174(DE3)	This study
pHK-TT1A-*qseC*476a	Derived from pHK-TT1A, targeting the sense strand 476 site of *ptsG* in HMS174(DE3)	This study

Table S6 Targeting efficiency of 13 plasmids with $T_{(-8)}$ and 18 plasmids with $A/C/G_{(-8)}$ at EBS2b site of redesigned targets.

**No.***	**Targeting Sites**	**Recognition Sequences (-13 to -1)**	**Targeting Efficiency**
IBS2b site is 'T' base			
1	*qseC*38a	AAAGATTAGCGTC	83.0±13.0%
2	*qseC*244s	CACGGTCATGTTG	73.5±5.0%
3	*qseC*309a	TATCTTCTCCGTT	20.8±4.0%
4	*qseC*627s	ACCACTAAACGCG	83.3±8.0%
5	*qseC*1052a	CAAACTTAGCAAT	38.9±9.0%
6	*qseC*1073s	GTTTGTTGGTGCG	29.2±7.0%
7	*qseC*1294s	TTGCATGGCATGA	83.3±8.0%
8	*qseC*1330s	CAAGGTGGATTTG	83.0±13.0%
9	*ptsG*345s	ACACCTGGCGGAT	0%
10	*ptsG*1083a	TCGCATAACCGAT	0%
11	*ptsG*1230a	TGTTTTCTTTACC	38.9±13.0%
12	*ptsG*1359a	TCGCCTGAACACC	33.3±8.0%
13	*ptsG*1399s	TCCGATAACCTGA	79.2±4.0%
IBS2b site are 'A/C/G' bases			
1	*ptsG*498a	TGAAGGACAGCAC	0%
2	*ptsG*785s	TGTACGGTCTGCC	0%
3	*ptsG*849s	AGTGGGCGGTATT	26.4±6.4%
4	*qseC*568s	CTCGCGCCGCTGA	0%
5	*qseC*704a	GCGTCGTTCACGA	0%
6	*qseC*967s	TCGTCGGTGATGG	0%
7	*ptsG*198a	AGCCGAGGGCGAC	0%
8	*ptsG*388a	CGGTTAAACATGT	0%
9	*ptsG*584a	ACCGTAAATGCCA	58.3±8.3%
10	*qseC*115s	CAAACAACGGATA	0%
11	*qseC*457s	TGGGAATACCGTG	0%
12	*qseC*539a	TCATCATGATGGT	0%
13	*ptsG*21s	GAATGCATTTGCT	9.7±2.4%
14	*ptsG*95a	ATTCGCGGAACCG	0%
15	*ptsG*514a	CCAATCGGCGGCC	0%
16	*qseC*139s	TTGTTCGACACCC	0%
17	*qseC*257a	GATGGCAAAGGTC	0%
18	*qseC*476a	TTGCCCGGCAACA	0%

*Thirteen targeting sites were randomly selected in *qseC* and *ptsG*. ‘s’: sense strand; ‘a’: antisense strand. The bases (from -13 to -1) listed here are the key bases for gene-targeting recognition. Twenty-four colonies were randomly selected for PCR validation, and the gene-targeting efficiency was calculated as follows: gene-targeting efficiency = [(gene-inactivated colonies) / (total detected colonies)] × 100%.

Table S7 Primers used in three types of unpaired conditions (T/T, G/T, C/T).

**Primers**	**Sequences (5′ -3′) ***	**Note**
*qseC*38aIBS12	AGCCAAAGCAGGTTGACTAGTAAaaagattagcgtcGTGCGACGCGAAAGCTAG	EBS2b-IBS2b unpaired conditions ($T_{(-8)}$-$T_{(-8)}$, $G_{(-8)}$-$T_{(-8)}$, $C_{(-8)}$-$T_{(-8)}$), targeting *qseC*38a
*qseC*38aEBS1a	CGGAGTTGCTGTCCCCGTACGCTGAagcgtcAGCAGCGtATCCAATCC	
*qseC*38aEBS2s(T)	CGCTAGAAGCCTCGTTTtctttAGCAGGCCAAAGATGCTG	
*qseC*38aEBS2s(C)	CGCTAGAAGCCTCGTTCtctttAGCAGGCCAAAGATGCTG	
*qseC*38aEBS2s(G)	CGCTAGAAGCCTCGTTGtctttAGCAGGCCAAAGATGCTG	
*qseC*627sIBS12	AGCCAAAGCAGGTTGACTAGTAAaccactaaacgcgGTGCGACGCGAAAGCTAG	EBS2b-IBS2b unpaired conditions ($T_{(-8)}$-$T_{(-8)}$, $G_{(-8)}$-$T_{(-8)}$, $C_{(-8)}$-$T_{(-8)}$), targeting *qseC*627s
*qseC*627sEBS1a	CGGAGTTGCTGTCCCCGTACGCTGAaacgcgAGCAGCGTATCCAATCC	
*qseC*627sEBS2s(T)	CGCTAGAAGCCTCGTTTgtggtAGCAGGCCAAAGATGCTG	
*qseC*627sEBS2s(C)	CGCTAGAAGCCTCGTTCgtggtAGCAGGCCAAAGATGCTG	
*qseC*627sEBS2s(G)	CGCTAGAAGCCTCGTTGgtggtAGCAGGCCAAAGATGCTG	
*qseC*1294sIBS12	AGCCAAAGCAGGTTGACTAGTAAttgcatggcatgaGTGCGACGCGAAAGCTAG	EBS2b-IBS2b unpaired conditions ($T_{(-8)}$-$T_{(-8)}$, $G_{(-8)}$-$T_{(-8)}$, $C_{(-8)}$-$T_{(-8)}$), targeting *qseC*1294s
*qseC*1294sEBS1a	CGGAGTTGCTGTCCCCGTACGCTGAgcatgaAGCAGCGTATCCAATCC	
*qseC*1294sEBS2s(T)	CGCTAGAAGCCTCGTTTgcaaAGCAGGCCAAAGATGCTG	
*qseC*1294sEBS2s(C)	CGCTAGAAGCCTCGTTCtgcaaAGCAGGCCAAAGATGCTG	
*qseC*1294sEBS2s(G)	CGCTAGAAGCCTCGTTGtgcaaAGCAGGCCAAAGATGCTG	
*ptsG*1230aIBS12	AGCCAAAGCAGGTTGACTAGTAAtgttttctttaccGTGCGACGCGAAAGCTAG	EBS2b-IBS2b unpaired conditions ($T_{(-8)}$-$T_{(-8)}$, $G_{(-8)}$-$T_{(-8)}$, $C_{(-8)}$-$T_{(-8)}$), targeting *ptsG*1230a
*ptsG*1230aEBS1a	CGGAGTTGCTGTCCCCGTACGCTGAtttaccAGCAGCGtATCCAATCC	
*ptsG*1230aEBS2s(T)	AGCCAAAGCAGGTTGATTAGTAAtgttttctttaccGTGCGACGCGAAAGCTAG	
*ptsG*1230aEBS2s(C)	CGCTAGAAGCCTCGTTCaaacaAGCAGGCCAAAGATGCTG	
*ptsG*1230aEBS2s(G)	CGCTAGAAGCCTCGTTGaaacaAGCAGGCCAAAGATGCTG	
*ptsG*1359aIBS12	AGCCAAAGCAGGTTGACTAGTAAtcgcctgaacaccGTGCGACGCGAAAGCTAG	EBS2b-IBS2b unpaired conditions ($T_{(-8)}$-$T_{(-8)}$, $G_{(-8)}$-$T_{(-8)}$, $C_{(-8)}$-$T_{(-8)}$), targeting *ptsG*1359a
*ptsG*1359aEBS1a	CGGAGTTGCTGTCCCCGTACGCTGAaacaccAGCAGCGtATCCAATCC	
*ptsG*1359aEBS2s(T)	CGCTAGAAGCCTCGTTTggcgaAGCAGGCCAAAGATGCTG	
*ptsG*1359aEBS2s(C)	CGCTAGAAGCCTCGTTCggcgaAGCAGGCCAAAGATGCTG	
*ptsG*1359aEBS2s(G)	CGCTAGAAGCCTCGTTGggcgaAGCAGGCCAAAGATGCTG	
*ptsG*1399sIBS12	AGCCAAAGCAGGTTGACTAGTAAtccgataacctgaGTGCGACGCGAAAGCTAG	EBS2b-IBS2b unpaired conditions ($T_{(-8)}$-$T_{(-8)}$, $G_{(-8)}$-$T_{(-8)}$, $C_{(-8)}$-$T_{(-8)}$), targeting *ptsG*1399s
*ptsG*1399sEBS1a	CGGAGTTGCTGTCCCCGTACGCTGAacctgaAGCAGCGTATCCAATCC	
*ptsG*1399sEBS2s(T)	CGCTAGAAGCCTCGTTTtcggaAGCAGGCCAAAGATGCTG	
*ptsG*1399sEBS2s(C)	CGCTAGAAGCCTCGTTCtcggaAGCAGGCCAAAGATGCTG	
*ptsG*1399sEBS2s(G)	CGCTAGAAGCCTCGTTGtcggaAGCAGGCCAAAGATGCTG	

Table S8 Strains and plasmids used in three types of unpaired conditions (T/T, G/T, C/T).

**Strains and plasmids**	**Characteristics**	**Resource or reference**
**Strains**		
NEBExpress Competent *E. coli* (High efficiency)	*fhuA2* [*Ion*] *ompT gal sulA11 R*(*mcr-73::*mini*Tn10*--Tet^S)*2*[*dcm*] *R*(*zgb-210::Tn10*--Tet^S)*endA1*delta(*mcrC-mrr*)*114::lS10*	NEB
E. coli HMS174(DE3)	F^- *recA1 hsdR*($r_{K12}^- m_{K12}^+$) (DE3) (Rif^R)	Novogene
Δ*ptsG*1359a(G)	Derived from *E. coli* HMS174(DE3), Δ*ptsG* 1359a(G)	This study
Δ*ptsG*1399s(C)	Derived from *E. coli* HMS174(DE3), Δ*ptsG*1399s(C)	This study
**Plasmids**		
pHK-TT1A	Targetron vector, GroEL promoter, Cm^R	[1]
pHK-TT1A-*qseC*38a(T)	Derived from pHK-TT1A(T), targeting the antisense strand 38 site of *qseC* in HMS174(DE3)	This study
pHK-TT1A-*qseC*38a(C)	Derived from pHK-TT1A(C), targeting the antisense strand 38 site of *qseC* in HMS174(DE3)	This study
pHK-TT1A-*qseC*38a(G)	Derived from pHK-TT1A(G), targeting the antisense strand 38 site of *qseC* in HMS174(DE3)	This study
pHK-TT1A-*qseC*627s(T)	Derived from pHK-TT1A(T), targeting the sense strand 627 site of *qseC* in HMS174(DE3)	This study
pHK-TT1A-*qseC*627s(C)	Derived from pHK-TT1A(C), targeting the sense strand 627 site of *qseC* in HMS174(DE3)	This study
pHK-TT1A-*qseC*627s(G)	Derived from pHK-TT1A(G), targeting the sense strand 627 site of *qseC* in HMS174(DE3)	This study
pHK-TT1A-*qseC*1294s(T)	Derived from pHK-TT1A(T), targeting the sense strand 1294 site of *qseC* in HMS174(DE3)	This study
pHK-TT1A-*qseC*1294s(C)	Derived from pHK-TT1A(C), targeting the sense strand 1294 site of *qseC* in HMS174(DE3)	This study
pHK-TT1A-*qseC*1294s(G)	Derived from pHK-TT1A(G), targeting the sense strand 1294 site of *qseC* in HMS174(DE3)	This study
pHK-TT1A-*ptsG*1230a(T)	Derived from pHK-TT1A(T), targeting the antisense strand 1230 site of *ptsG* in HMS174(DE3)	This study
pHK-TT1A-*ptsG*1230a(C)	Derived from pHK-TT1A(C), targeting the antisense strand 1230 site of *ptsG* in HMS174(DE3)	This study
pHK-TT1A-*ptsG*1230a(G)	Derived from pHK-TT1A(G), targeting the antisense strand 1230 site of *ptsG* in HMS174(DE3)	This study
pHK-TT1A-*ptsG*1359a(T)	Derived from pHK-TT1A(T), targeting the antisense strand 1359 site of *ptsG* in HMS174(DE3)	This study
pHK-TT1A-*ptsG*1359a(C)	Derived from pHK-TT1A(C), targeting the antisense strand 1359 site of *ptsG* in HMS174(DE3)	This study
pHK-TT1A-*ptsG*1359a(G)	Derived from pHK-TT1A(G), targeting the antisense strand 1359 site of *ptsG* in HMS174(DE3)	This study
pHK-TT1A-*ptsG*1399s(T)	Derived from pHK-TT1A(T), targeting the sense strand 1399 site of *ptsG* in HMS174(DE3)	This study
pHK-TT1A-*ptsG*1399s(C)	Derived from pHK-TT1A(C), targeting the sense strand 1399 site of *ptsG* in HMS174(DE3)	This study

pHK-TT1A-*ptsG*1399s(G)	Derived from pHK-TT1A(G), targeting the sense strand 1399 site of *ptsG* in HMS174(DE3)	This study

Table S9 Gene-targeting efficiency of 18 plasmids in three types of unpaired conditions (T/T, G/T, C/T).

**No.***	**Targeting Sites**	**Recognition Sequences (-13 to -1)**	**Targeting Efficiency**
1	*qseC*38a(T)		0%
2	*qseC*38a(C)	AAAGATTAGCGTC	0%
3	*qseC*38a(G)		0%
4	*qseC*627s(T)		0%
5	*qseC*627s(C)	ACCACTAAACGCG	0%
6	*qseC*627s(G)		0%
7	*qseC*1294s(T)		0%
8	*qseC*1294s(C)	TTGCATGGCATGA	0%
9	*qseC*1294s(G)		0%
10	*ptsG*1230a(T)		0%
11	*ptsG*1230a(C)	TGTTTTCTTTACC	0%
12	*ptsG*1230a(G)		0%
13	*ptsG*1359a(T)		0%
14	*ptsG*1359a(C)	TCGCCTGAACACC	0%
15	*ptsG*1359a(G)		64.0±5.2%
16	*ptsG*1399s(T)		0%
17	*ptsG*1399s(C)	TCCGATAACCTGA	84.2±5.5%
18	*ptsG*1399s(G)		0%

Table S10 Primers used in non-A/T (T/A, G/C, and C/G) base paring conditions.

**Primers**	**Sequences (5′-3′) ***	**Note**
*ptsG*498aIBS12	AGCCAAAGCAGGTTGACTAGTAAtgaaggacagcacGTGCGACGCGAAAGCTAG	
*ptsG*498aEBS2s(C)	CGCTAGAAGCCTCGTTCcttcaAGCAGGCCAAAGATGCTG	EBS2s(C), targeting *ptsG*498a
*ptsG*498aEBS1a	CGGAGTTGCTGTCCCCGTACGCTGAcagcacAGCAGCGtATCCAATCC	
*ptsG*785sIBS12	AGCCAAAGCAGGTTGACTAGTAAtgtacggtctgccGTGCGACGCGAAAGCTAG	
*ptsG*785sEBS2s(C)	CGCTAGAAGCCTCGTTCgtacaAGCAGGCCAAAGATGCTG	EBS2s(C), targeting *ptsG*785s
*ptsG*785sEBS1a	CGGAGTTGCTGTCCCCGTACGCTGAtctgccAGCAGCGTATCCAATCC	
*ptsG*849sIBS12	AGCCAAAGCAGGTTGACTAGTAAagtgggcggtattGTGCGACGCGAAAGCTAG	
*ptsG*849sEBS2s(C)	CGCTAGAAGCCTCGTTCccactAGCAGGCCAAAGATGCTG	EBS2s(C), targeting *ptsG*849s
*ptsG*849sEBS1a	CGGAGTTGCTGTCCCCGTACGCTGAggtattAGCAGCGTATCCAATCC	
*qseC*568sIBS12	AGCCAAAGCAGGTTGACTAGTAActcgcgccgctgaGTGCGACGCGAAAGCTAG	
*qseC*568sEBS2s(C)	CGCTAGAAGCCTCGTTCgcgagAGCAGGCCAAAGATGCTG	EBS2s(C), targeting *qseC*568
*qseC*568sEBS1a	CGGAGTTGCTGTCCCCGTACGCTGAcgctgaAGCAGCGTATCCAATCC	
*qseC*704aIBS12	AGCCAAAGCAGGTTGACTAGTAAgcgtcgttcacgaGTGCGACGCGAAAGCTAG	
*qseC*704aEBS2s(C)	CGCTAGAAGCCTCGTTCgacgcAGCAGGCCAAAGATGCTG	EBS2s(C), targeting *qseC*704a
*qseC*704aEBS1a	CGGAGTTGCTGTCCCCGTACGCTGAtcacgaAGCAGCGtATCCAATCC	
*qseC*967sIBS12	AGCCAAAGCAGGTTGACTAGTAAtcgtcggtgatggGTGCGACGCGAAAGCTAG	
*qseC*967sEBS2s(C)	CGCTAGAAGCCTCGTTCgacgaAGCAGGCCAAAGATGCTG	EBS2s(C), targeting *qseC*967s
*qseC*967sEBS1a	CGGAGTTGCTGTCCCCGTACGCTGAtgatggAGCAGCGTATCCAATCC	
*lacZ*168aIBS12	AGCCAAAGCAGGTTGACTAGTAAagcgccattcgccGTGCGACGCGAAAGCTAG	
*lacZ*168aEBS2s(C)	CGCTAGAAGCCTCGTTGgcgctAGCAGGCCAAAGATGCTG	EBS2s(C), targeting *lacZ*168a
*lacZ*168aEBS1a	CGGAGTTGCTGTCCCCGTACGCTGAttcgccAGCAGCGtATCCAATCC	
*lacZ*321sIBS12	AGCCAAAGCAGGTTGACTAGTAAcgtgacctatcccGTGCGACGCGAAAGCTAG	
*lacZ*321sEBS2s(G)	CGCTAGAAGCCTCGTTGtcacgAGCAGGCCAAAGATGCTG	EBS2s(G), targeting *lacZ*321s
*lacZ*321sEBS1a	CGGAGTTGCTGTCCCCGTACGCTGAtatcccAGCAGCGTATCCAATCC	
*dctA*146aIBS12	AGCCAAAGCAGGTTGACTAGTAAgatgacaggagcgGTGCGACGCGAAAGCTAG	
*dctA*146aEBS2s(G)	CGCTAGAAGCCTCGTTGtcatcAGCAGGCCAAAGATGCTG	EBS2s(G), targeting *dctA*146a
*dctA*146aEBS1a	CGGAGTTGCTGTCCCCGTACGCTGAggagcgAGCAGCGTATCCAATCC	
*dctA*18aIBS12	AGCCAAAGCAGGTTGACTAGTAAagtaaaggcttttGTGCGACGCGAAAGCTAG	
*dctA*18aEBS2s(T)	CGCTAGAAGCCTCGTTTttactAGCAGGCCAAAGATGCTG	EBS2s(T), targeting *dctA*18a
*dctA*18aEBS1a	CGGAGTTGCTGTCCCCGTACGCTGAgcttttAGCAGCGTATCCAATCC	
*glcD*698aIBS12	AGCCAAAGCAGGTTGACTAGTAAcagaacccgcgccGTGCGACGCGAAAGCTAG	
*glcD*698aEBS2s(G)	CGCTAGAAGCCTCGTTGttctgAGCAGGCCAAAGATGCTG	EBS2s(G), targeting *glcD*698a
*glcD*698aEBS1a	CGGAGTTGCTGTCCCCGTACGCTGAcgcgccAGCAGCGTATCCAATCC	
*ptsG*198aIBS12	AGCCAAAGCAGGTTGACTAGTAAagccgagggcgacGTGCGACGCGAAAGCTAG	
*ptsG*198aEBS2s(T)	CGCTAGAAGCCTCGTTTcggctAGCAGGCCAAAGATGCTG	EBS2s(T), targeting *ptsG*198a
*ptsG*198aEBS1a	CGGAGTTGCTGTCCCCGTACGCTGAggcgacAGCAGCGtATCCAATCC	
*ptsG*388aIBS12	AGCCAAAGCAGGTTGACTAGTAAcggttaaacatgtGTGCGACGCGAAAGCTAG	
*ptsG*388aEBS2s(T)	CGCTAGAAGCCTCGTTTaaccgAGCAGGCCAAAGATGCTG	EBS2s(T), targeting *ptsG*388a
*ptsG*388aEBS1a	CGGAGTTGCTGTCCCCGTACGCTGAacatgtAGCAGCGtATCCAATCC	
*ptsG*584aIBS12	AGCCAAAGCAGGTTGACTAGTAAaccgtaaatgccaGTGCGACGCGAAAGCTAG	
*ptsG*584aEBS2s(T)	CGCTAGAAGCCTCGTTTacggtAGCAGGCCAAAGATGCTG	EBS2s(T), targeting *ptsG*584a
*ptsG*584aEBS1a	CGGAGTTGCTGTCCCCGTACGCTGAatgccaAGCAGCGtATCCAATCC	
*qseC*115sIBS12	AGCCAAAGCAGGTTGACTAGTAAcaaacaacggataGTGCGACGCGAAAGCTAG	EBS2s(T), targeting *qseC*115s
*qseC*115sEBS2s(T)	CGCTAGAAGCCTCGTTTgtttgAGCAGGCCAAAGATGCTG	

*qseC*115sEBS1a	CGGAGTTGCTGTCCCCGTACGCTGAcggataAGCAGCGTATCCAATCC	
*qseC*457sIBS12	AGCCAAAGCAGGTTGACTAGTAAtgggaataccgtgGTGCGACGCGAAAGCTAG	
*qseC*457sEBS2s(T)	CGCTAGAAGCCTCGTTTtcccaAGCAGGCCAAAGATGCTG	EBS2s(T), targeting *qseC*457s
*qseC*457sEBS1a	CGGAGTTGCTGTCCCCGTACGCTGAaccgtgAGCAGCGTATCCAATCC	
*fliC*510aIBS12	AGCCAAAGCAGGTTGACTAGTAAcgctaaaaccatcGTGCGACGCGAAAGCTAG	
*fliC*510aEBS2s(T)	CGCTAGAAGCCTCGTTTtagcgAGCAGGCCAAAGATGCTG	EBS2s(T), targeting *fliC*510a
*fliC*510aEBS1a	CGGAGTTGCTGTCCCCGTACGCTGAaccatcAGCAGCGtATCCAATCC	
*fliC*735aIBS12	AGCCAAAGCAGGTTGACTAGTAActgttactgcgtaGTGCGACGCGAAAGCTAG	
*fliC*735aEBS2s(T)	CGCTAGAAGCCTCGTTTaacagAGCAGGCCAAAGATGCTG	EBS2s(T), targeting *fliC*735a
*fliC*735aEBS1a	CGGAGTTGCTGTCCCCGTACGCTGAtgcgtaAGCAGCGtATCCAATCC	
*lacZ*906sIBS12	AGCCAAAGCAGGTTGACTAGTAAcccgaaactgtggGTGCGACGCGAAAGCTAG	
*lacZ*906sEBS2s(T)	CGCTAGAAGCCTCGTTTtcgggAGCAGGCCAAAGATGCTG	EBS2s(T), targeting *lacZ*906s
*lacZ*906sEBS1a	CGGAGTTGCTGTCCCCGTACGCTGActgtggAGCAGCGTATCCAATCC	
*glcD*555sIBS12	AGCCAAAGCAGGTTGACTAGTAAaattgaagtgcaaGTGCGACGCGAAAGCTAG	
*glcD*555sEBS2s(T)	CGCTAGAAGCCTCGTTTcaattAGCAGGCCAAAGATGCT G	EBS2s(T), targeting *glcD*555s
*glcD*555sEBS1a	CGGAGTTGCTGTCCCCGTACGCTGAgtgcaaAGCAGCGT ATCCAATCC	
*ptsG*21sIBS12	AGCCAAAGCAGGTTGACTAGTAAgaatgcatttgctGTGCGACGCGAAAGCTAG	
*ptsG*21sEBS2s(T)	CGCTAGAAGCCTCGTTGcattcAGCAGGCCAAAGATGCTG	EBS2s(T), targeting *ptsG*21s
*ptsG*21sEBS1a	CGGAGTTGCTGTCCCCGTACGCTGAtttgctAGCAGCGTATCCAATCC	
*ptsG*95aIBS12	AGCCAAAGCAGGTTGACTAGTAAattcgcggaaccgGTGCGACGCGAAAGCTAG	
*ptsG*95aEBS2s(G)	CGCTAGAAGCCTCGTTGcgaatAGCAGGCCAAAGATGCTG	EBS2s(G), targeting *ptsG*95a
*ptsG*95aEBS1a	CGGAGTTGCTGTCCCCGTACGCTGAgaaccgAGCAGCGtATCCAATCC	
*ptsG*514aIBS12	AGCCAAAGCAGGTTGACTAGTAAccaatcggcggccGTGCGACGCGAAAGCTAG	
*ptsG*514aEBS2s(G)	CGCTAGAAGCCTCGTTGattggAGCAGGCCAAAGATGCTG	EBS2s(G), targeting *ptsG*514a
*ptsG*514aEBS1a	CGGAGTTGCTGTCCCCGTACGCTGAgcggccAGCAGCGtATCCAATCC	
*qseC*139sIBS12	AGCCAAAGCAGGTTGACTAGTAAttgttcgacacccGTGCGACGCGAAAGCTAG	
*qseC*139sEBS2s(G)	CGCTAGAAGCCTCGTTGaacaaAGCAGGCCAAAGATGCTG	EBS2s(G), targeting *qseC*139s
*qseC*139sEBS1a	CGGAGTTGCTGTCCCCGTACGCTGAacacccAGCAGCGTATCCAATCC	
*qseC*257aIBS12	AGCCAAAGCAGGTTGACTAGTAAgatggcaaaggtcGTGCGACGCGAAAGCTAG	
*qseC*257aEBS2s(G)	CGCTAGAAGCCTCGTTGccatcAGCAGGCCAAAGATGCTG	EBS2s(G), targeting *qseC*257a
*qseC*257aEBS1a	CGGAGTTGCTGTCCCCGTACGCTGAaaggtcAGCAGCGtATCCAATCC	
*qseC*476aIBS12	AGCCAAAGCAGGTTGACTAGTAAttgcccggcaacaGTGCGACGCGAAAGCTAG	
*qseC*476aEBS2s(G)	CGCTAGAAGCCTCGTTGggcaaAGCAGGCCAAAGATGCTG	EBS2s(G), targeting *qseC*476a
*qseC*476aEBS1a	CGGAGTTGCTGTCCCCGTACGCTGAgcaacaAGCAGCGtATCCAATCC	
*fliC*250sIBS12	AGCCAAAGCAGGTTGACTAGTAAggcgcgctgtccgGTGCGACGCGAAAGCTAG	
*fliC*250sEBS2s(G)	CGCTAGAAGCCTCGTTCgcgccAGCAGGCCAAAGATGCTG	EBS2s(G), targeting *fliC*250s
*fliC*250sEBS1a	CGGAGTTGCTGTCCCCGTACGCTGAtgtccgAGCAGCGTATCCAATCC	
*dctA*227aIBS12	AGCCAAAGCAGGTTGACTAGTAAgtaaagcagtgcgGTGCGACGCGAAAGCTAG	
*dctA*227aEBS2s(C)	CGCTAGAAGCCTCGTTCtttacAGCAGGCCAAAGATGCTG	EBS2s(G), targeting *dctA*227a
*dctA*227aEBS1a	CGGAGTTGCTGTCCCCGTACGCTGAagtgcgAGCAGCGTATCCAATCC	
*glcD*448aIBS12	AGCCAAAGCAGGTTGACTAGTAAcaggcgatttgtgGTGCG ACGCGAAAGCTAG	
*glcD*448aEBS2s(C)	CGCTAGAAGCCTCGTTCgcctgAGCAGGCCAAAGATGC TG	EBS2s(G), targeting *glcD*448a
*glcD*448aEBS1a	CGGAGTTGCTGTCCCCGTACGCTGAtttgtgAGCAGCGTA TCCAATCC	

Table S11 Strains and plasmids used in non-A/T paired (T/A, G/C, and C/G) conditions.

Strains and plasmids	Characteristics	Resource or reference
**Strains**		
NEBExpress Competent *E. coli* (High efficiency)	*fhuA2* [*Ion*] *ompT gal sulA11 R*(*mcr-73::*mini*Tn10*--Tet^S)*2*[*dcm*] *R*(*zgb-210::Tn10*--Tet^S)*endA1*delta(*mcrC-mrr*)*114::lS10*	NEB
E. coli HMS174(DE3)	F^- *recA1 hsdR*($r_{K12}^- m_{K12}^+$) (DE3) (Rif^R)	Novogene
Δ*ptsG*849s(C)	Derived from *E. coli* HMS174(DE3), Δ*ptsG*849s(C)	This study
Δ*ptsG*584a(T)	Derived from *E. coli* HMS174(DE3), Δ*ptsG*584a(T)	This study
Δ*ptsG*21s(G)	Derived from *E. coli* HMS174(DE3), Δ*ptsG*21s(G)	This study
**Plasmids**		
pHK-TT1A	Targetron vector, GroEL promoter, Cm^R	[1]
pHK-TT1A-*ptsG*498a(C)	Derived from pHK-TT1A(C), targeting the antisense strand 498 site of *qseC* in HMS174(DE3)	This study
pHK-TT1A-*ptsG*785s(C)	Derived from pHK-TT1A(C), targeting the sense strand 785 site of *qseC* in HMS174(DE3)	This study
pHK-TT1A-*ptsG*849s(C)	Derived from pHK-TT1A(C), targeting the sense strand 849 site of *qseC* in HMS174(DE3)	This study
pHK-TT1A-*qseC*568s(C)	Derived from pHK-TT1A(C), targeting the sense strand 568 site of *qseC* in HMS174(DE3)	This study
pHK-TT1A-*qseC*704a(C)	Derived from pHK-TT1A(C), targeting the antisense strand 704 site of *qseC* in HMS174(DE3)	This study
pHK-TT1A-*qseC*967s(C)	Derived from pHK-TT1A(C), targeting the sense strand 967 site of *qseC* in HMS174(DE3)	This study
pHK-TT1A-*lacZ*168a(G)	Derived from pHK-TT1A(G), targeting the antisense strand 168 site of *qseC* in HMS174(DE3)	This study
pHK-TT1A-*lacZ*321s(G)	Derived from pHK-TT1A(G), targeting the sense strand 321 site of *qseC* in HMS174(DE3)	This study
pHK-TT1A-*dctA*146a(G)	Derived from pHK-TT1A(G), targeting the antisense strand 146 site of *qseC* in HMS174(DE3)	This study
pHK-TT1A-*dctA*18a(G)	Derived from pHK-TT1A(G), targeting the antisense strand 18 site of *ptsG* in HMS174(DE3)	This study
pHK-TT1A-*glcD*698a(G)	Derived from pHK-TT1A(G), targeting the antisense strand 698 site of *ptsG* in HMS174(DE3)	This study
pHK-TT1A-*ptsG*198a(T)	Derived from pHK-TT1A(T), targeting the antisense strand 198 site of *ptsG* in HMS174(DE3)	This study
pHK-TT1A-*ptsG*388a(T)	Derived from pHK-TT1A(T), targeting the antisense strand 388 site of *ptsG* in HMS174(DE3)	This study
pHK-TT1A-*ptsG*584a(T)	Derived from pHK-TT1A(T), targeting the antisense strand 584 site of *ptsG* in HMS174(DE3)	This study
pHK-TT1A-*qseC*115s(T)	Derived from pHK-TT1A(T), targeting the sense strand 115 site of *ptsG* in HMS174(DE3)	This study
pHK-TT1A-*qseC*457s(T)	Derived from pHK-TT1A(T), targeting the sense strand 457site of *ptsG* in HMS174(DE3)	This study
pHK-TT1A-*fliC*510a(T)	Derived from pHK-TT1A(T), targeting the sense antistrand 510 site of *ptsG* in HMS174(DE3)	This study
pHK-TT1A-*fliC*735a(T)	Derived from pHK-TT1A(T), targeting the antisense strand 735 site of *ptsG* in HMS174(DE3)	This study
pHK-TT1A-*lacZ*906s(T)	Derived from pHK-TT1A(T), targeting the sense strand 906 site of *ptsG* in HMS174(DE3)	This study
pHK-TT1A-	Derived from pHK-TT1A(T), targeting the sense strand 555 site of *ptsG* in HMS174(DE3)	This study

*glcD*555s(T)		
pHK-TT1A-*ptsG*21s(G)	Derived from pHK-TT1A(G), targeting the sense strand 21 site of *ptsG* in HMS174(DE3)	This study
pHK-TT1A-*ptsG*95a(G)	Derived from pHK-TT1A(G), targeting the antisense strand 95 site of *ptsG* in HMS174(DE3)	This study
pHK-TT1A-*ptsG*514a(G)	Derived from pHK-TT1A(G), targeting the antisense strand 514 site of *ptsG* in HMS174(DE3)	This study
pHK-TT1A-*qseC*139s(G)	Derived from pHK-TT1A(G), targeting the sense strand 139 site of *ptsG* in HMS174(DE3)	This study
pHK-TT1A-*qseC*257a(G)	Derived from pHK-TT1A(G), targeting the antisense strand 257 site of *ptsG* in HMS174(DE3)	This study
pHK-TT1A-*qseC*476a(G)	Derived from pHK-TT1A(G), targeting the antisense strand 476 site of *ptsG* in HMS174(DE3)	This study
pHK-TT1A-*fliC*250s(G)	Derived from pHK-TT1A(G), targeting the sense strand 250 site of *ptsG* in HMS174(DE3)	This study
pHK-TT1A-*dctA*227a(G)	Derived from pHK-TT1A(G), targeting the antisense strand 227 site of *ptsG* in HMS174(DE3)	This study
pHK-TT1A-*glcD*448a(G)	Derived from pHK-TT1A(G), targeting the sense antistrand 448 site of *ptsG* in HMS174(DE3)	This study

Table S12 Gene-targeting efficiency of non-A/T paired (T/A, G/C, and C/G) conditions.

No.*	Targeting Sites	Recognition Sequences (-13 to -1)	Targeting Efficiency
1	*ptsG*498a(C)	TGAAGGACAGCAC	0%
2	*ptsG*785s(C)	TGTACGGTCTGCC	0%
3	*ptsG*849s(C)	AGTGGGCGGTATT	0%
4	*qseC*568s(C)	CTCGCGCCGCTGA	0%
5	*qseC*704a(C)	GCGTCGTTCACGA	0%
6	*qseC*967s(C)	TCGTCGGTGATGG	0%
7	*lacZ*168a(G)	AGCGCCATTCGCC	0%
8	*lacZ*321s(G)	CGTGACCTATCCC	0%
9	*dctA*146a(G)	GATGACAGGAGCG	6.9±2.4%
10	*dctA*18a(G)	AACCTCTCTGTTT	0%
11	*glcD*698a(G)	CAGAACCCGCGCC	0%
12	*ptsG*198a(T)	AGCCGAGGGCGAC	0%
13	*ptsG*388a(T)	CGGTTAAACATGT	0%
14	*ptsG*584a(T)	ACCGTAAATGCCA	0%
15	*qseC*115s(T)	CAAACAACGGATA	0%
16	*qseC*457s(T)	TGGGAATACCGTG	0%
17	*fliC*510a(T)	CGCTAAAACCATC	0%
18	*fliC*735a(T)	CTGTTACTGCGTA	50.9±16.2%
19	*lacZ*906s(T)	CCCGAAACTGTGG	0%
20	*glcD*555s(T)	AATTGAAGTGCAA	0%
21	*ptsG*21s(G)	GAATGCATTTGCT	5.6±4.8%
22	*ptsG*95a(G)	ATTCGCGGAACCG	0%
23	*ptsG*514a(G)	CCAATCGGCGGCC	0%
24	*qseC*139s(G)	TTGTTCGACACCC	0%
25	*qseC*257a(G)	GATGGCAAAGGTC	0%
26	*qseC*476a(G)	TTGCCCGGCAACA	0%
27	*fliC*250s(C)	GGCGCGCTGTCCG	0%
28	*dctA*227a(C)	GTAAAGCAGTGCG	0%
29	*glcD*448a(C)	CAGGCGATTTGTG	0%

Table S13 The distribution of targeting sites on the DNA double-strand.

DNA double strand	Failed	Successful	Total
Sense(s)	76	28	104
Antisense(a)	52	25	77
Total	128	53	181

*(Chi-square test, two-sided, *P*=0.4177, not statistically significant)

[1] Mohr G, Hong W, Zhang J, Cui G, Yang Y, Cui Q, et al. A Targetron System for Gene Targeting in Thermophiles and Its Application in *Clostridium thermocellum*. PLoS ONE. 2013;8:e69032.

Supplementary file 1 (TMT 1.0.py**)**

```
ID=raw_input('Input the name of your target GENE:')
seqs=raw_input('Input the gene sequence from ATG/GTG:')
index=0
for i in range(0,len(seqs)-15):
 if seqs[index].lower()=='a' and seqs[index+1].lower()=='a' and seqs[index+15].lower()=='a' and
seqs[index+7].lower()=='t':
    x=seqs[index:index+16].lower()
    y=x.lower().count('c')
    z=x.lower().count('g')
    formate1='%-15s%-20s%-20s%-s'
    formate2='%-15s%-17s%-25s%-s'
    #formate3='%*s'
    #Targetron reverse complementary sequence, export EBS2=o[9:14]
    f=x.replace('a','2')
    g=f.replace('t','1')
    h=g.replace('g','4')
    j=h.replace('c','3')
    k=j.replace('1','a')
    l=k.replace('2','t')
    m=l.replace('3','g')
    n=m.replace('4','c')
    o=n[16::-1]
    p=i+15
    IBS12='%s%s%s%s%s-AAAACTAGTAA%sGTGCGACGCGAAAGCTAG'
    EBS2='%s%s%s%s%s-CGCTAGAAGCCTCGTTA%sAGCAGGCCAAAGATGCTG'
    EBS1a='%s%s%s%s%s-CCCCGTACGCTGA%sAGCAGCGTATCCAATCC'
    print '='*65
    print formate1 % ('Strand','Sites','Targetron','(G+C)%')
    print formate2 % ('S',i+15,x,(y+z)*100/13)
    #print formate3 % (48,'△')
    #print 'Thermo-targetron generating primers are ready!'
    print '-'*65
    print IBS12 % (ID.upper(),p,'s','-','IBS12',x[2:15].lower())
    print EBS2 % (ID.upper(),p,'s','-','IBS2s',o[9:14].lower())
    print EBS1a % (ID.upper(),p,'s','-','IBS1a',x[9:15].lower())
    print '='*65
    print ' '*65

 if seqs[index].lower()=='t' and seqs[index+14].lower()=='t' and seqs[index+15].lower()=='t' and
seqs[index+8].lower()=='a':
    x=seqs[index:index+16].lower()
    y=x.lower().count('c')
    z=x.lower().count('g')
    formate1='%-15s%-20s%-20s%-s'
```

```
     formate2='%-15s%-17s%-25s%-s'
     #formate3='%*s'
     #x reverse complementary sequence to obtain targete sequence, which is e=d[16::-1]
     u=x.replace('a','2')
     t=u.replace('t','1')
     s=t.replace('g','4')
     r=s.replace('c','3')
     a=r.replace('1','a')
     b=a.replace('2','t')
     c=b.replace('3','g')
     d=c.replace('4','c')
     e=d[16::-1]
     #Targetron reverse complementary sequence, output EBS2=o[9:14].
     f=e.replace('a','2')
     g=f.replace('t','1')
     h=g.replace('g','4')
     j=h.replace('c','3')
     k=j.replace('1','a')
     l=k.replace('2','t')
     m=l.replace('3','g')
     n=m.replace('4','c')
     o=n[16::-1]
     p=i+1
     IBS12='%s%s%s%s%s-AAAACTAGTAA%sGTGCGACGCGAAAGCTAG'
     EBS2='%s%s%s%s%s-CGCTAGAAGCCTCGTTA%sAGCAGGCCAAAGATGCTG'
     EBS1a='%s%s%s%s%s-CCCCGTACGCTGA%sAGCAGCGtATCCAATCC'
     print '='*65
     print formate1 % ('Strand','Sites','Targetron','(G+C)%')
     print formate2 % ('A',i+1,e,(y+z)*100/13)
     #print formate3 % (48,'△')
     #print 'Thermotargetron primers are ready!'
     print '-'*65
     print IBS12 % (ID.upper(),p,'a','-','IBS12',e[2:15].lower())
     print EBS2 % (ID.upper(),p,'a','-','IBS2s',o[9:14].lower())
     print EBS1a % (ID.upper(),p,'a','-','IBS1a',e[9:15].lower())
     print '='*65
     print ' '*65
 index+=1

else:
   print '*'*65
   print 'NO MORE TARGETES SITES!'
   #print '△ Indicate insertion sites!'
   print 'All rights reserved, Wei Hong 07-21-2022'
   print '*'*65
```
